# Diametric calix[6]arene-based phosphine gold(I) cavitands

**DOI:** 10.3762/bjoc.18.21

**Published:** 2022-02-10

**Authors:** Gabriele Giovanardi, Andrea Secchi, Arturo Arduini, Gianpiero Cera

**Affiliations:** 1 Dipartimento di Scienze Chimiche, della Vita e della Sostenibilità Ambientale, Università di Parma, Parco Area delle Scienze 17/A, 43124 Parma, Italy

**Keywords:** 1,2,3-alternate conformation, calix[6]arenes, gold(I) catalysis, phosphines

## Abstract

We report the synthesis and characterization, in low polarity solvents, of a novel class of diametric phosphine gold(I) cavitands characterized by a *1,2,3-alternate* geometry. Preliminary catalytic studies were performed on a model cycloisomerization of 1,6-enynes as a function of the relative orientation of the bonded gold(I) nuclei with respect to the macrocyclic cavity.

## Introduction

**Figure 1:**
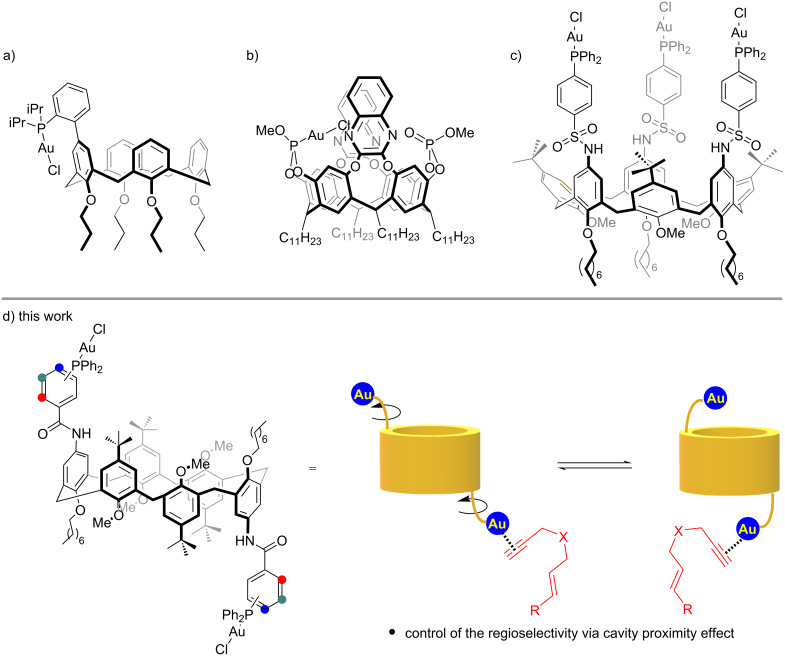
Selected examples of: a) calix[4]arene-; b) resorcin[4]arene-; c) calix[6]arene-gold(I) macrocyclic catalysts. d) Working hypothesis for novel calix[6]arene phosphine cavitands.

One of the latest challenges in supramolecular chemistry is the design and development of novel macrocyclic-based entities able to influence the catalytic activities of the metal center [1–3]. In this context, phosphines represent the most exploited class of ligands in homogeneous catalysis [4]. Noteworthy, reason of their wide applicability is the possibility of controlling the steric and electronic properties by proper functionalizations, hence tuning the catalytic properties of the bonded metal. This crucial aspect prompted their application in supramolecular chemistry as well. Thus, a recent evolution of their chemistry concerns the development of novel architectures in which P(III) compounds are incorporated in cavity-shaped macrocycles [5–8]. In this scenario, calix[4]- [9–13] and resorcin[4]arene [14–17] are the most exploited cavitands due to their inherent limited flexibility and already proved their ability to control the catalytic activity of late-transition metals and particularly gold(I) catalysts [18–25]. This occurs via strong steric interactions, often outside the macrocycle (Figure 1a) [11], that affect the first coordination sphere of the metal or by creating a spatial confinement around the metal that is thus directed towards the inner cavity (Figure 1b) [26–27]. Contrarily, calix[6]arene macrocycles are less exploited in catalysis [28]. The larger macrocycle size, its conformational adaptability, and the possibility to selectively functionalize the macrocycle offered several opportunities to design synthetic receptors and prototypes of nanodevices, instead [29]. In this context, we recently devised a new family of triphosphine calix[6]arene gold(I) complexes (Figure 1c) [30]. These cavitands are able to form (pseudo)rotaxane species, by threading viologen-based guests, with a conformational control operated by the sulfonamido hydrogen-bonding donor domain [31–32]. Furthermore, their catalytic activity was demonstrated in promoting gold(I)-catalyzed cycloisomerization of 1,6-enynes, with ample scope and high regioselectivity. However, preliminary studies suggested that the catalytic event occurs outside the macrocyclic cavity. In order to get more insights on the role of the cavity to dictate the position of the metal centers, we reasoned on the possibility to design a novel generation of diametric phosphine gold(I) cavitands exploiting a calix[6]arene scaffold characterized by a *1,2,3-alternate* conformation. As working hypothesis, this geometry would segregate two catalytically active gold(I) nuclei to the opposite sides of the macrocycle, offering them the possibility to approach the cavity, thus exerting any control over the catalytic manifold (Figure 1d).

## Results and Discussion

### Synthesis and characterization

**Scheme 1:**
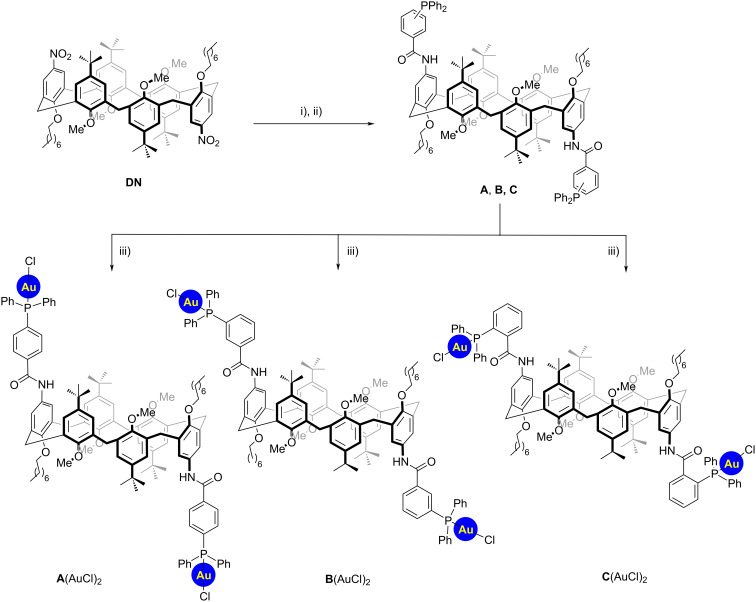
i) NH_2_NH_2_∙H_2_O, Pd/C in EtOH, 80 °C (quant.); ii) diphenylphosphinobenzoic acid, EDC∙HCl, DMAP (cat.), CH_2_Cl_2_, 0 °C to rt [**A**, 60%; **B**, 55%, **C**, 53%); iii) (Me_2_S)AuCl in CH_2_Cl_2_, 0 °C to rt (**A**(AuCl)_2_, 93%; **B**(AuCl)_2_, 74%; **C**(AuCl)_2_, 69%).

The synthesis of novel macrocyclic calix[6]arene ligands was first attempted starting from the known dioctyloxydinitro derivative **DN** (Scheme 1) [33]. Reduction of the nitro groups with hydrazine, in the presence of catalytic amounts of Pd/C (10 mol %) led to the corresponding diamino intermediate. This latter could be subsequently reacted with the desired phosphino benzoic acid derivative through a user-friendly amide coupling in the presence of EDC·HCl and catalytic amounts of DMAP in CH_2_Cl_2_. Under these conditions, the corresponding diphosphine intermediates **A** (*para*), **B** (*meta*), and **C** (*ortho*) were isolated in moderate yields. Finally, gold(I) catalysts could be obtained via conventional protocols using (Me_2_S)AuCl. Notably, the organometallic macrocycles **A**,**B**,**C**(AuCl)_2_ could be isolated via column chromatography separation.

**Figure 2:**
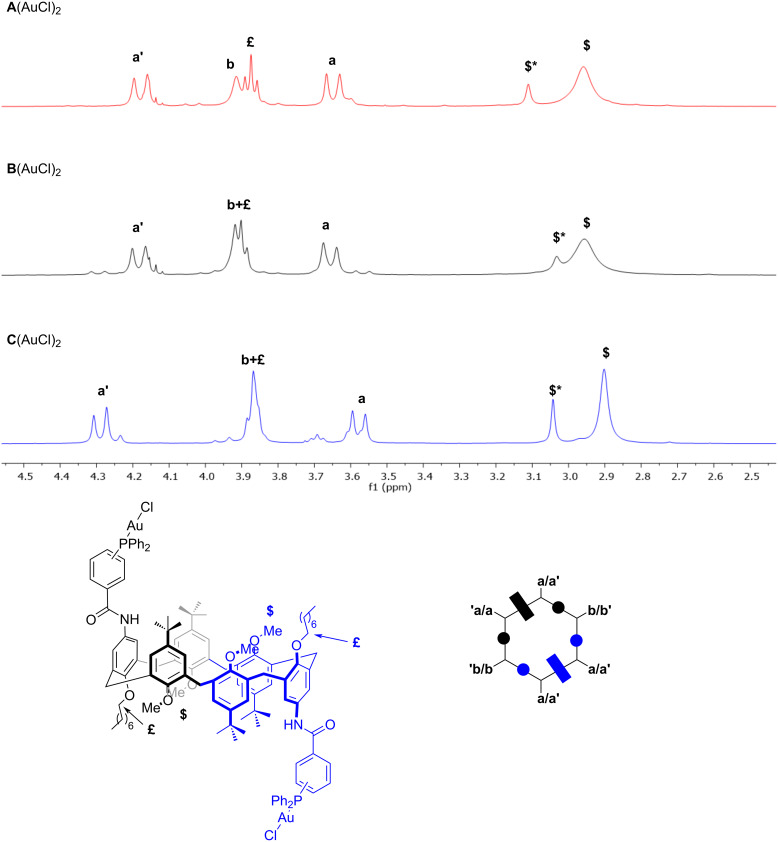
Stacked-plot, mid-field expanded region of the ^1^H NMR spectrum (400 MHz, 298 K) of **A**(AuCl)_2_, **B**(AuCl)_2_, and **C**(AuCl)_2_ in CDCl_3_. At the bottom are schematic representations of calix[6]arene macrocycles. The rectangle identifies the phenolic ring substituted with the octyloxy chains, while the circle identifies those with methoxy groups.

Gold(I) catalysts were subsequently fully characterized by NMR analysis and high-resolution mass spectrometry. The conformation of the catalysts, in low polarity solvents, is dominated by the *1,2,3-alternate* conformation assumed by the **DN** intermediate, as previously demonstrated in our recent contributions [33–34]. Hence, the most notable features of ^1^H NMR for **A**(AuCl)_2_ are represented by a pattern for the methylene bridging protons in a 1:1:1 integration ratio (Figure 2). These include: i) two doublets at 4.2 and 3.6 ppm with a geminal coupling of ^2^*J* = 14.2 Hz for the a/a' couple and ii) a singlet at 3.92 ppm for the b/b' couple, typical of an *anti*-orientation [35]. This situation suggests a single inversion point which confers to the macrocycle a high symmetrical geometry. Finally, a single broad peak for the four methoxy groups ($) appears at 2.96 ppm. In analogy with parental diureido and dithioureido calix[6]arenes, we were able to observe the presence of a second minor *cone* conformer, in a ≈4:1 ratio, highlighted by the presence of a second, single resonance for the methoxy groups ($*) at 3.11 ppm. An analogous situation was observed for **B**(AuCl)_2_ and **C**(AuCl)_2_ as well. However, here the singlets for the b/b’ couple overlap with the signals of the octyloxy chains (£) at 3.91 and 3.87 ppm, respectively.

**Figure 3:**
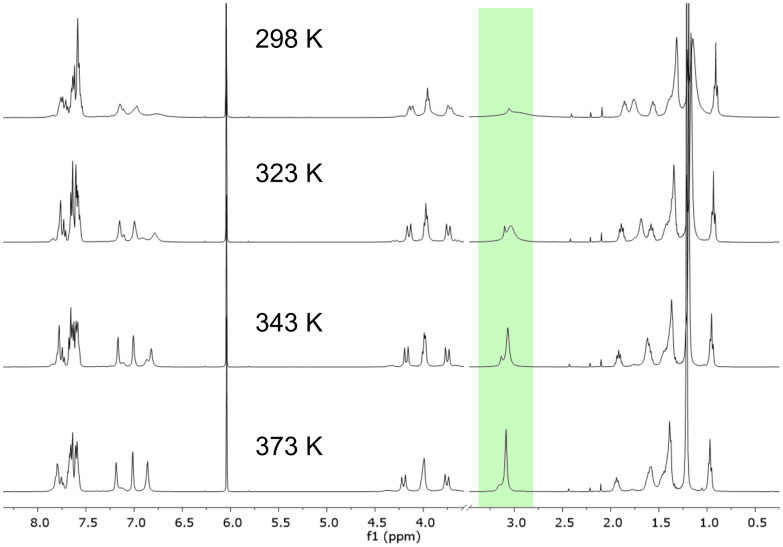
Stacked plot ^1^H NMR (tetrachloroethane-*d*_2_) of **A**(AuCl)_2_ at variable temperature.

The presence of these two major conformers, in slow exchange on the NMR timescale, was finally confirmed by variable temperature NMR analysis performed for **A**(AuCl)_2_ using tetrachloroethane-*d*_2_ as the solvent (Figure 3).

### Catalytic studies

**Scheme 2:**
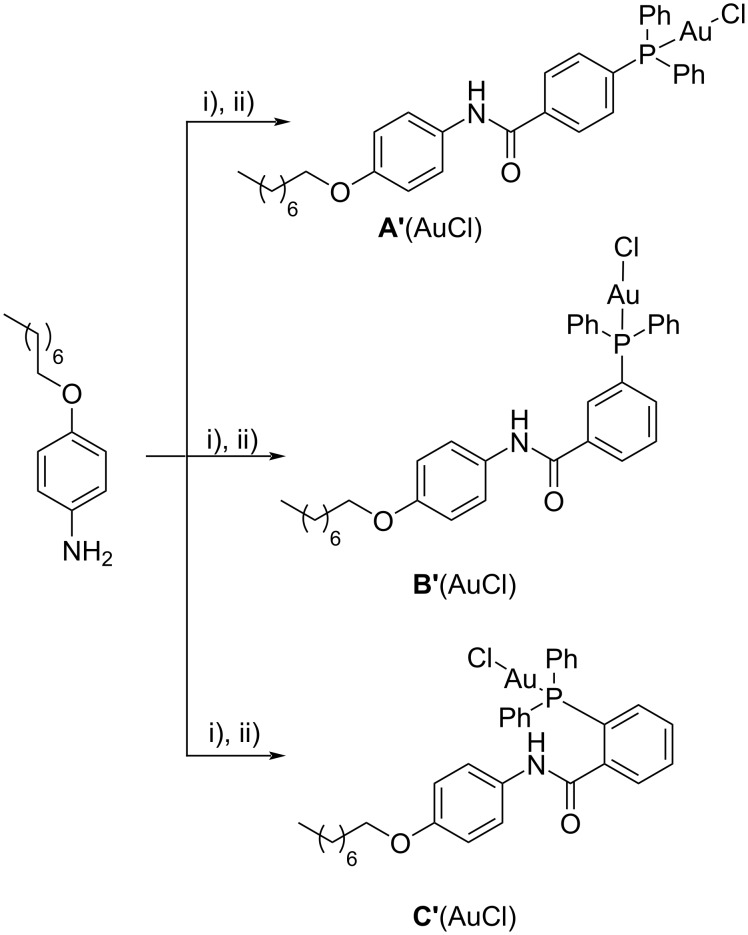
Synthesis of the monomeric gold catalyst analogues **A’**,**B’**,**C’**(AuCl). Conditions: i) diphenylphosphinobenzoic acid, EDC∙HCl, DMAP (cat.), CH_2_Cl_2_, 0 °C to rt (**A’**, 71%; **B’**, 76%; **C’**, 69%); ii) (Me_2_S)AuCl in DCM, 0 °C to rt (**A’**(AuCl), 94%; **B’**(AuCl), 92%; **C’**(AuCl), 96%).

To probe the role of the cavity and the influence of the position of the gold(I) nuclei implanted on the calix[6]arene scaffold, we carried out the synthesis of three monomeric gold catalyst analogues **A’**,**B’**,**C’**(AuCl). The synthesis of these compounds was performed using the previously optimized protocol, starting from a 4-(octyloxy)aniline intermediate (Scheme 2).

Subsequently, due to the general interest in controlling the reactivity of gold(I)-catalyzed transformations by means of supramolecular macrocycles, we choose a cycloisomerization of 1,6-enynes as a model reaction [36]. Substrate **1a** was reacted in the presence of monomeric gold(I) catalyst **A’**(AuCl) (2 mol %), using AgSbF_6_ as the chloride scavenger [37]. After 4 h, NMR analysis of the crude reaction mixture revealed high conversion of the starting material with the formation of the 6-*endo*-*dig* rearranged diene **2a** and the parental regioisomer **2b** in a 1:1 ratio. Noteworthy, this latter is formed by an initial 5-*exo*-*dig* cyclization step (entry 1, Table 1) [38–39]. This result was compared with the one obtained using the macrocyclic analogue **A**(AuCl)_2_ (1 mol %). Hence, we did not observe a significant variation in the product distribution (entry 2, Table 1).

**Table 1:** Ligand effect in gold(I)-catalyzed cycloisomerization of **1a**.

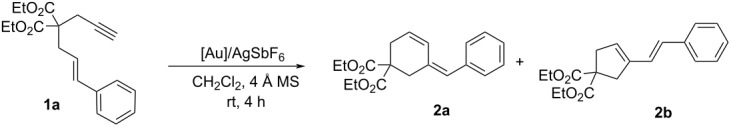			

Entry^a^	[Au]	conv. [%]	**2a**/**2b**

1	**A’**(AuCl) (2 mol %)	89	1.0:1.0
2	**A**(AuCl)_2_ (1 mol %)	88	1.1:1.0
3	**B’**(AuCl) (2 mol %)	86	1.2:1.0
4	**B**(AuCl)_2_ (1 mol %)	91	1.1:1.0
5	**C’**(AuCl) (2 mol %)	86	1.5:1.0
6	**C**(AuCl)_2_ (1 mol %)	89	1.8:1.0

^a^Reaction conditions: **1a** (0.2 mmol), AgSbF_6_ (2.0 mol %), CH_2_Cl_2_ (0.1 M), 4 h.

Analogously, the reactivity in the presence of *meta*-substituted catalysts **B’**(AuCl) and **B**(AuCl)_2_ was investigated. Also in this case, the reactivity and selectivity of the parental catalysts were comparable (entries 3 and 4, Table 1). Taken together, these outcomes suggest that the role of the macrocycle for catalysts **A/B**(AuCl)_2_ is not determining in changing the product distribution and that the catalytically active gold(I) nuclei are too far to be influenced by the cavity or by the macrocycle itself.

Finally, the catalytic reaction was attempted using **C’**(AuCl). We thus observed a selectivity towards product **2a** (1.5:1) which might be caused by the different orientation of the phosphine ligand implanted on the aromatic ring (entry 5, Table 1). Interestingly, this effect was substantially improved with the use of the calix[6]arene-based complex **C**(AuCl)_2_ (entry 6, Table 1). Overall, the *ortho*-substituted macrocycle **C**(AuCl)_2_ displayed an enhanced selectivity, with respect to the parental macrocycles **A**,**B**(AuCl)_2_, that arise from the proximity of the two gold(I) nuclei to the calix[6]arene scaffold. Although just preliminary, these results indicate that the conformational properties of this class of macrocycles can influence the selectivity in gold(I)-catalyzed cycloisomerization of 1,6-enynes.

## Conclusion

We reported the synthesis of a novel family of diametric diphosphine gold(I) complexes whose geometry in low-polarity solvents is controlled by the *1,2,3-alternate* conformation of the calix[6]arene precursor. These catalysts are able to tune the selectivity of catalytic cycloisomerization of 1,6-enynes as a function of the relative orientation of the bonded gold(I) nuclei with respect to the macrocycle. Further studies are currently under progress to outline the role of the macrocycle and to verify if a possible re-orientation of the gold(I) nuclei towards the center of the aromatic cavity can play any role in dictating the selectivity of the catalytic transformation.

## Experimental

General procedure for catalysis: In a 10 mL two-necked round-bottomed flask containing **A**(AuCl)_2_ (1.0 mol %, 4.4 mg) or **A’**(AuCl) (2.0 mol %, 3.0 mg), dry CH_2_Cl_2_ (2.0 mL) was added under nitrogen atmosphere. Subsequently, a tip of spatula (micro spatula, Heyman type 16 cm) of AgSbF_6_ (≈2.0 mol %, ≈2 mg) was added along with 20 mg of 4 Å molecular sieves. The flask was covered with an aluminum foil and the mixture stirred for 5 minutes. Subsequently, **1a** (0.2 mmol, 63.0 mg) was added and the reaction mixture was stirred for 4 hours. After completion, the mixture was diluted with 20 mL of CH_2_Cl_2_, filtered through a pad of celite, and transferred in a 100 mL flask where it was concentrated under reduced atmosphere. Conversions and selectivities were determined by ^1^H NMR analysis (data confirmed by performing the reaction twice). ^1^H NMR (**2a**) (400 MHz, CDCl_3_) δ 7.48–7.19 (m, 5H), 6.63 (dtd, *J* = 10.1, 2.1, 1.0 Hz, 1H), 6.37 (s, 1H), 5.90 (dtd, *J* = 10.1, 4.0, 1.6 Hz, 1H), 4.30–4.21 (m, 4H), 2.98 (d, *J* = 1.6 Hz, 2H), 2.78 (ddd, *J* = 4.0, 2.1, 0.9 Hz, 2H), 1.32–1.24 (m, 6H). ^1^H NMR (**2b**) (400 MHz, CDCl_3_) δ 7.48–7.20 (m, 5H), 6.93 (d, *J* = 16.2 Hz, 1H), 6.48 (d, *J* = 16.2 Hz, 1H), 5.73 (ddd, *J* = 2.7, 1.9, 0.9 Hz, 1H), 4.23–4.14 (m, 4H), 3.28 (dd, *J* = 1.9, 0.9 Hz, 2H), 3.18 (dd, *J* = 1.9, 0.9 Hz, 2H), 1.32–1.21 (m, 6H).

## Supporting Information

File 1 Experimental procedures, characterization data of compounds and copies of NMR spectra.
