# Supplementary material for: Diametric calix[6]arene-based phosphine gold(I) cavitands
Source: Beilstein J Org Chem. 2022 Feb 10;18:190–6. doi: 10.3762/bjoc.18.21 (PMC8848346; doi:10.3762/bjoc.18.21)
Supplement: File 1 — Experimental procedures, characterization data of compounds and copies of NMR spectra. [file Beilstein_J_Org_Chem-18-190-s001.pdf]

BEILSTEIN JOURNAL OF ORGANIC CHEMISTRY

Supporting Information

for

Diametric calix[6]arene-based phosphine gold(I) cavitands

Gabriele Giovanardi, Andrea Secchi, Arturo Arduini and Gianpiero Cera

Beilstein J. Org. Chem. **2022,** *18,* 190–196. doi:10.3762/bjoc.18.21

Experimental procedures, characterization data of compounds and copies of NMR spectra

License and Terms: This is a supporting information file under the terms of the Creative Commons Attribution License (https://creativecommons.org/licenses/by/4.0). Please note that the reuse, redistribution and reproduction in particular requires that the author(s) and source are credited and that individual graphics may be subject to special legal provisions.
The license is subject to the *Beilstein Journal of Organic Chemistry* terms and conditions: (https://www.beilstein-journals.org/bjoc/terms)

Table of contents

General remarks and materials........S1

Synthesis and characterization of calixphos ligands **A**, **B**, **C**........S2

Synthesis and characterization of monomeric phosphine ligands **A'**, **B'**, **C'**........S5

Synthesis and characterization of calixphos catalysts **A**, **B**, **C**$(AuCl)_2$........S7

Synthesis and characterization of monomeric catalysts **A'**, **B'**, **C'**(AuCl)........S9

NMR spectra........S12

References........S40

• General remarks and materials

All chemicals those syntheses are not reported hereafter were purchased from commercial sources and used as received. Solvents were dried and stored over molecular sieves previously activated in an oven (450 °C over night). Anhydrous CH_2Cl_2 for catalytic reactions was supplied by Fluka in Sureseal® bottles and used without any further purification. Column chromatography was performed on silica gel 60 (70–230 mesh). Melting points were measured with an electrothermal apparatus and are uncorrected. NMR spectra were recorded on a Bruker 400 MHz using solvents as internal standards (7.26 ppm for ^{1}H NMR and 77.00 ppm for ^{13}C NMR for $CDCl_3$). The terms m, s, d, t, q, and quint represent multiplet, singlet, doublet, triplet, quadruplet, and quintuplet respectively, and the term br means a broad signal. ^{13}C APT NMR spectra are reported for compounds. Exact masses were recorded on a LTQ ORBITRAP XL Thermo Mass Spectrometer (ESI source). Materials: **DN** derivative [1], (diphenylphosphino)benzoic acids [2], and enyne **1a** [3] were synthesized according to known procedures.

- **Synthesis and characterization of calixphos ligands A, B, and C**

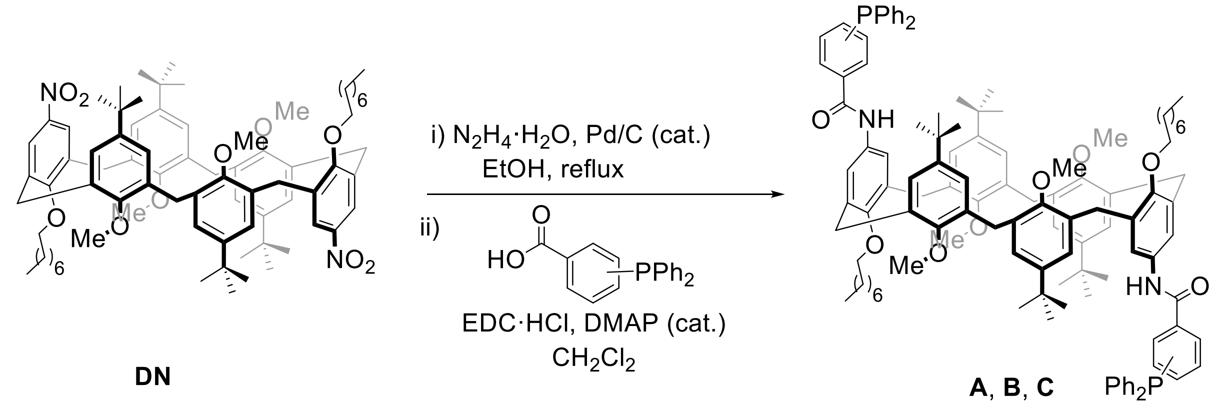

To a two-necked round-bottomed flask, under N_2 atmosphere, Pd/C (10 mol %) was added to a suspension of **DN** (234 mg, 0.27 mmol) in EtOH (100 mL). Subsequently, NH_2NH_2•H_2O (5 mmol, 20 equiv) was added dropwise, and after the addition was completed, the reaction mixture was refluxed at 80 °C for 24 h (oil bath). After completion of the reaction as determined by TLC analysis, the reaction mixture was cooled to room temperature, and then filtered through a Celite pad to remove the solids. The mixture was concentrated at reduced pressure and water (30 mL) was added. After extraction with CH_2Cl_2 (3 × 30 mL), organic phases were dried over Na_2SO_4 and concentrated at reduced pressure to afford a pale yellow solid. The crude was dissolved in dry CH_2Cl_2 (15 mL) under N_2 atmosphere and DMAP (10 mol %) and EDC•HCl (0.8 mmol, 3.0 equiv) were added. Subsequently, the mixture was cooled to 0 °C and the corresponding (diphenylphosphino)benzoic acid (0.7 mmol, 2.5 equiv) was added and the reaction mixture was stirred for 16 h. After completion, H_2O (20 mL) was added, and the mixture extracted with CH_2Cl_2 (3 × 30 mL). The organic layers were dried over Na_2SO_4, concentrated at reduced pressure, and the crude purified by column chromatography on silica gel (*n*-Hex/AcOEt 80:20).

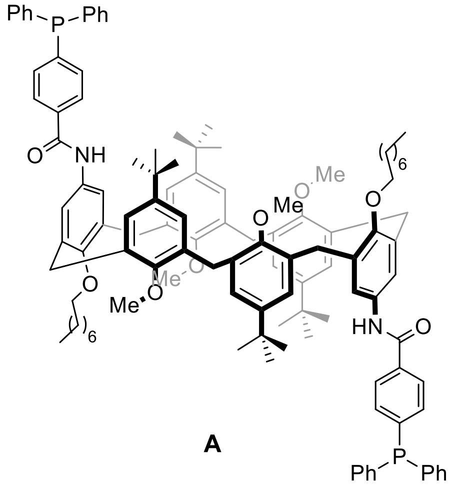

A

General procedure was followed using 4-(diphenylphosphino)benzoic acid. Purification by column chromatography on silica gel (*n*-Hex/EtOAc 80:20) yielded **A** (285 mg, 60%) as a white solid. **M. p.** = 130-133 °C. **^{1}H NMR** *1,2,3-alternate conformer* (400 MHz, $CDCl_3$) δ = 7.79 – 7.64 (m, 4H), 7.45 – 7.23 (m, 28H), 7.23 (d, *J* = 2.5 Hz, 4H), 6.94 (br s, 4H), 6.89 (br s, 2H), 4.21 (d, *J* = 14.2 Hz, 4H), 3.93 (s, 4H), 3.88 (t, *J* = 6.6 Hz, 4H), 3.66 (d, *J* = 14.2 Hz, 4H), 2.94 (br s, 12H), 1.90 – 1.79 (m, 4H), 1.61 –1.51 (m, 4H), 1.44 – 1.27 (m, 16H), 1.17 (s, 36H), 0.91 (s, 6H). **^{13}C NMR** *1,2,3-alternate conformer* (101 MHz, $CDCl_3$) δ = 165.0 (C_q), 154.0 (C_q), 151.6 (C_q), 146.3 (2 x C_q), 142.0 (d, J_{C-P} = 13.8 Hz, C_q), 136.5 (d, J_{C-P} = 10.8 Hz, C_q), 135.3 (C_q), 135.1 (C_q), 132.9 (C_q), 133.8 (d, J_{C-P} = 19.8 Hz, CH), 133.7 (d, J_{C-P} = 17.2 Hz, CH), 132.8 (C_q), 129.1 (CH), 128.7 (d, J_{C-P} = 7.3 Hz, CH), 127.1 (d, J_{C-P} = 6.6 Hz, CH), 126.8 (CH), 126.6 (CH), 121.2 (CH), 73.3 (CH_2), 60.0 (CH_3), 34.1 (C_q), 31.9 (CH_2), 31.4 (CH_3), 30.8 (2 x CH_2), 30.5 (CH_2), 29.6 (CH_2), 29.3 (CH_2), 26.3 (CH_2), 22.7 (CH_2), 14.2 (CH_3). **^{31}P NMR** *1,2,3-alternated conformer* (162 MHz, $CDCl_3$) δ = -5.36. **ESI-MS**: *m/z* $[M+Na]^+$ calcd. for $C_{116}H_{136}N_2NaO_8P_2$: 1769.97; found: 1769.52. **HR-MS** (ESI) *m/z*: $[M+H]^+$ calcd. for $C_{116}H_{137}N_2O_8P_2$: 1747.9884; found 1747.9886.

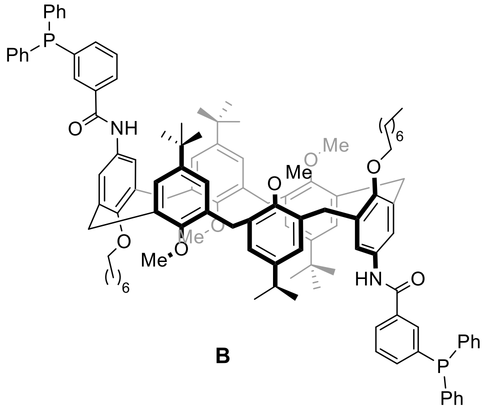

B

General procedure was followed using 3-(diphenylphosphino)benzoic acid. Purification by column chromatography on silica gel (*n*-Hex/EtOAc 80:20) yielded **B** (261 mg, 55%) as a white solid. **M. p.** = 171-173 °C. **^{1}H NMR** *1,2,3-alternated conformer* (400 MHz, $CDCl_3$) δ = 7.80 – 7.73 (m, 2H), 7.72 – 7.75 (m, 6H), 7.54 – 7.41 (m, 6H), 7.40 – 7.19 (m, 20H), 6.98 (br s, 4H), 6.86 (br s, 4H), 4.23 (d, *J* = 14.2 Hz, 4H), 3.92 (bs, 4H), 3.88 (t, *J* = 7.9 Hz, 4H), 3.62 (d, *J* = 14.2 Hz, 4H), 2.86 (br s, 12H), 1.97 – 1.83 (m, 4H), 1.65 – 1.53 (m, 4H), 1.45 – 1.29 (m, 16H), 1.15 (s, 36H), 0.94 – 0.82 (m, 6H). **^{13}C NMR** *1,2,3-alternated conformer* (101 MHz, $CDCl_3$) δ = 164.8 (C_q), 156.3 (C_q), 151.7 (C_q), 146.2 (C_q), 138.4 (d, J_{C-P} = 12.1 Hz, C_q), 136.4 (d, J_{C-P} = 15.8 Hz, CH), 136.3 (d, J_{C-P} = 9.6 Hz, C_q), 135.5 (C_q), 135.3 (d, J_{C-P} = 7.2 Hz, C_q), 133.8 (C_q), 133.7 (d, J_{C-P} = 19.2 Hz, CH), 133.0 (C_q), 132.9 (C_q), 132.1 (d, J_{C-P} = 9.4 Hz, CH), 129.1 (CH), 128.8 (d, J_{C-P} = 7.0 Hz, CH), 128.7 (d, J_{C-P} = 7.0 Hz, CH), 127.4 (CH), 126.8 (CH), 126.4 (CH), 120.7 (CH), 73.2 (CH_2), 59.9 (CH_3), 34.1 (C_q), 31.9 (CH_2), 31.3 (CH_3), 30.7 (CH_2), 30.6 (2 x CH_2), 29.6 (CH_2), 29.3 (CH_2), 26.4 (CH_2), 22.7 (CH_2), 14.1 (CH_3). **^{31}P NMR**

1,2,3-alternate conformer (162 MHz, $CDCl_3$) δ = -3.22. **ESI-MS**: *m/z* $[M+Na]^+$ calcd. for $C_{116}H_{136}N_2NaO_8P_2$: 1769.97; found: 1769.13. **HR-MS** (ESI) *m/z*: $[M+H]^+$ calcd. for $C_{116}H_{137}N_2O_8P_2$: 1747.9884; found 1747.9894.

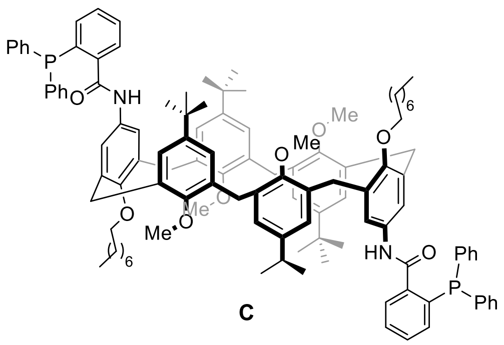

C

General procedure was followed using 2-(diphenylphosphino)benzoic acid. Purification by column chromatography on silica gel (*n*-Hex/EtOAc 80:20) yielded **C** (250 mg, 53%) as a white solid. **M. p.** = 175-172 °C. **^{1}H NMR** *1,2,3-alternate conformer* (400 MHz, $CDCl_3$) δ = 7.63–7.58 (m, 2H), 7.47 – 7.42 (m, 2H), 7.39 – 7.16 (m, 26H), 7.09 – 7.25 (m, 6H), 6.91 – 6.87 (m, 6H), 4.27 (d, *J* = 14.3 Hz, 4H), 3.90 (s, 4H), 3.88 (t, *J* = 7.9 Hz, 4H), 3.57 (d, *J* = 14.3 Hz, 4H), 2.85 (br s, 12H), 1.95 – 1.84 (m, 4H), 1.64 – 1.52 (m, 4H), 1.47 – 1.28 (m, 16H), 1.18 (s, 36H), 0.94 – 0.88 (m, 6H). **^{13}C NMR** *1,2,3-alternate conformer* (101 MHz, $CDCl_3$) δ = 166.5 (C_q), 154.2 (C_q), 151.8 (C_q), 146.0 (C_q), 142.0 (d, J_{C-P} = 25.0 Hz, C_q), 136.9 (d, J_{C-P} = 6.4 Hz, C_q), 135.1 (C_q), 135.0 (d, J_{C-P} = 15.0 Hz, C_q), 134.2 (CH), 133.9 (d, J_{C-P} = 18.8 Hz, CH), 133.7 (C_q), 133.2 (C_q), 132.7 (C_q), 130.2 (CH), 128.9 (CH), 128.8 (CH), 128.5 (d, J_{C-P} = 7.8 Hz, CH), 128.0 (d, J_{C-P} = 5.3 Hz, CH), 126.9 (CH), 126.3 (CH), 120.7 (CH), 73.5 (CH_2), 59.9 (CH_3), 34.1 (C_q), 31.9 (CH_2), 31.4 (CH_3), 30.9 (CH_2), 30.6 (2 x CH_2), 29.6 (CH_2), 29.3 (CH_2), 26.3 (CH_2), 22.7 (CH_2), 14.1 (CH_3). **^{31}P NMR** *1,2,3-alternate conformer* (162 MHz, $CDCl_3$) δ = -8.67. **ESI-MS**: *m/z* $[M+Na]^+$ calcd. for $C_{116}H_{136}N_2NaO_8P_2$: 1769.97; found: 1769.32. **HR-MS** (ESI) *m/z*: $[M+H]^+$ calcd. for $C_{116}H_{137}N_2O_8P_2$: 1747.9884; found 1747.9896.

- **Synthesis and characterization of monomeric phosphine ligands**

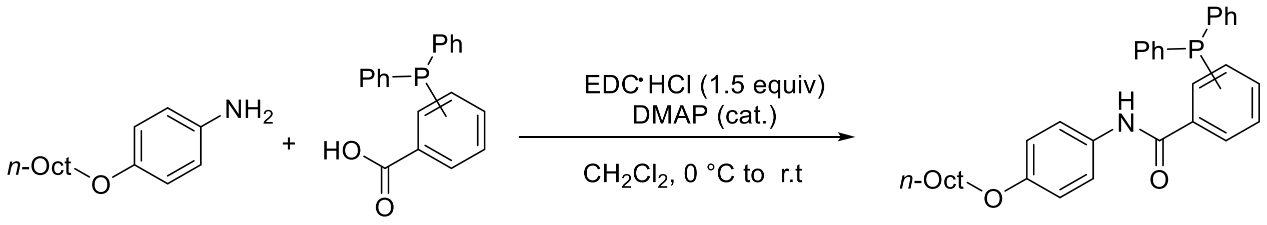

4-(Octyloxy)aniline (0.7 mmol, 1.0 equiv) was dissolved in dry CH_2Cl_2 (15 mL) under N_2 atmosphere and DMAP (10 mol %) and EDC•HCl (0.8 mmol, 1.1 equiv) were added. Subsequently, the mixture was cooled to 0 °C and the desired (diphenylphosphino)benzoic acid (0.8 mmol, 1.1 equiv) was added. The reaction mixture was stirred for 16 h. After completion, H_2O (20 mL) was added, and the mixture extracted with CH_2Cl_2 (3 × 30 mL). The organic layers were dried over Na_2SO_4, concentrated at reduced pressure, and the crude purified by column chromatography on silica gel (*n*-Hex/AcOEt 80:20).

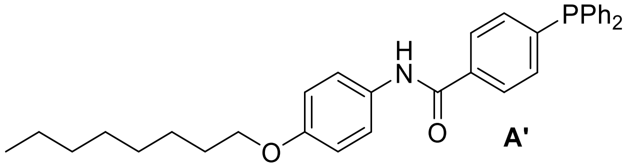

A'

General procedure was followed using 4-(diphenylphosphino)benzoic acid. Purification by column chromatography on silica gel (*n*-Hex/EtOAc 80:20) yielded **A’** (255 mg, 71%) as a white solid. **M. p.** = 138-140 °C. **^{1}H NMR** (400 MHz, $CDCl_3$) δ = 7.85 (s, 1H), 7.84 – 7.78 (d, *J* = 8.2 Hz, 2H), 7.56 – 7.50 (d, *J* = 8.2 Hz, 2H), 7.41 – 7.32 (m, 12H), 6.91 (d, *J* = 9.0 Hz, 2H), 3.97 (t, *J* = 6.6 Hz, 2H), 1.85 – 1.73 (m, 2H), 1.54 – 1.43 (m, 2H), 1.38 – 1.27 (m, 8H), 0.96 – 0.83 (m, 3H). **^{13}C NMR** (101 MHz, $CDCl_3$) δ = 165.3 (C_q), 156.3 (C_q), 142.1 (d, $J_{C\text{-}P}$ = 12.9 Hz, C_q), 136.0 (d, $J_{C\text{-}P}$ = 9.3 Hz, C_q), 135.1 (C_q), 133.9 (d, $J_{C\text{-}P}$ = 19.6 Hz, CH), 133.7 (d, $J_{C\text{-}P}$ = 19.0 Hz, CH), 130.7 (C_q), 129.2 (CH), 128.7 (d, $J_{C\text{-}P}$ = 7.3 Hz, CH), 126.9 (d, $J_{C\text{-}P}$ = 6.6 Hz, CH), 122.1 (CH), 114.9 (CH), 68.3 (CH_2), 31.9 (CH_2), 29.4 (CH_2), 29.3 (CH_2), 29.3 (CH_2), 26.1 (CH_2), 22.7 (CH_2), 14.1 (CH_3). **^{31}P NMR** (162 MHz, $CDCl_3$) δ = -3.18. **ESI-MS**: *m/z* $[M+Na]^+$ calcd. for $C_{33}H_{36}NNaO_2P$: 532.24; found: 532.27. **HR-MS** (ESI) *m/z*: $[M+H]^+$ calcd. for $C_{33}H_{37}NO_2P$: 510.2562; found 510.2556.

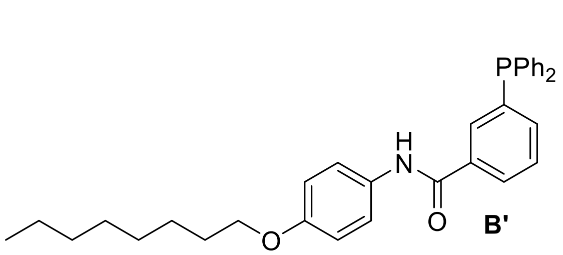

B'

General procedure was followed using 3-(diphenylphosphino)benzoic acid. Purification by column chromatography on silica gel (*n*-Hex/EtOAc 80:20) yielded **B'** (271 mg, 76%) as a white solid. **M. p.** = 137-138 °C. **^{1}H NMR** (400 MHz, $CDCl_3$) δ = 7.92 – 7.82 (m, 2H), 7.78 (s, 1H), 7.49 (d, *J* = 8.6 Hz, 2H), 7.45 (dd, *J* = 6.1, 2.6 Hz, 2H), 7.40 – 7.32 (m, 10H), 6.99 – 6.78 (m, 2H), 3.96 (t, *J* = 6.6 Hz, 2H), 1.85 – 1.73 (m, 2H), 1.47 (q, *J* = 7.0 Hz, 2H), 1.39 – 1.26 (m, 8H), 0.96 – 0.85 (m, 3H). **^{13}C NMR** (101 MHz, $CDCl_3$) δ = 165.2 (C_q), 156.2 (C_q), 138.4 (d, J_{C-P} = 12.1 Hz, C_q), 136.6 (d J_{C-P} = 15.8 Hz, CH), 136.1 (d, J_{C-P} = 9.6 Hz, C_q), 135.3 (d, J_{C-P} = 7.2 Hz, C_q), 133.8 (d, J_{C-P} = 19.9 Hz, CH), 131.9 (d, J_{C-P} = 23.6 Hz, CH), 130.7 (C_q), 129.2 (CH), 129.0 (d J_{C-P} = 5.6 Hz, CH), 128.7 (d, J_{C-P} = 7.0 Hz, CH), 127.8 (CH), 122.0 (CH), 114.9 (CH), 68.3 (CH_2), 31.8 (CH_2), 29.4 (CH_2), 29.3 (CH_2), 29.3 (CH_2), 26.1 (CH_2), 22.7 (CH_2), 14.1 (CH_3). **^{31}P NMR** (162 MHz, $CDCl_3$) δ = -2.99. **ESI-MS**: *m/z* $[M+Na]^+$ calcd. for $C_{33}H_{36}NNaO_2P$: 532.24; found: 532.26. **HR-MS** (ESI) *m/z*: $[M+H]^+$ calcd. for $C_{33}H_{37}NO_2P$: 510.2562; found 510.2568.

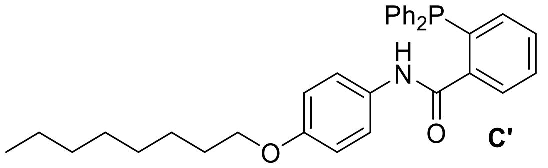

C'

General procedure was followed using 2-(diphenylphosphino)benzoic acid. Purification by column chromatography on silica gel (*n*-Hex/EtOAc 80:20) yielded **C'** (247 mg, 69%) as a white solid. **M. p.** = 117-118 °C. **^{1}H NMR** (400 MHz, $CDCl_3$) δ = 7.82 (dd, *J* = 8.0, 3.8 Hz, 1H), 7.75 (s, 1H), 7.46 (td, *J* = 7.5, 1.3 Hz, 1H), 7.42 – 7.31 (m, 11H), 7.24 – 7.19 (m, 2H), 7.05 (ddd, *J* = 7.7, 4.5, 1.3 Hz, 1H), 6.87 – 6.74 (m, 2H), 3.94 (t, *J* = 6.6 Hz, 2H), 1.88 – 1.70 (m, 2H), 1.52 – 1.41 (m, 2H), 1.40 – 1.25 (m, 8H), 0.97 – 0.82 (m, 3H). **^{13}C NMR** (101 MHz, $CDCl_3$) δ = 166.8 (C_q), 156.1 (C_q), 141.6 (d, J_{C-P} = 24.7 Hz, C_q), 135.9 (d, J_{C-P} = 6.4 Hz, C_q), 135.1 (d, J_{C-P} = 15.4 Hz, C_q), 134.1 (d, J_{C-P} = 5.3 Hz, CH), 134.0 (d, J_{C-P} = 20.0 Hz, CH), 130.5 (C_q), 130.4 (CH), 129.3 (2x CH), 128.8 (d, J_{C-P} = 7.3 Hz, CH), 128.6 (d, J_{C-P} = 5.1 Hz, CH), 121.7 (CH), 114.6 (CH), 68.3 (CH_2), 31.8 (CH_2), 29.4 (CH_2), 29.3 (CH_2), 29.3 (CH_2), 26.1 (CH_2), 22.7 (CH_2), 14.1 (CH_3). **^{31}P NMR** (162 MHz, $CDCl_3$) δ = -7.70. **ESI-MS**: *m/z* $[M+Na]^+$ calcd. for $C_{33}H_{36}NNaO_2P$: 532.24; found: 532.22. **HR-MS** (ESI) *m/z*: $[M+H]^+$ calcd. for $C_{33}H_{37}NO_2P$: 510.2562; found 510.2572.

- **Synthesis and characterization of calixphos catalysts A,B,C(AuCl)$_2$**

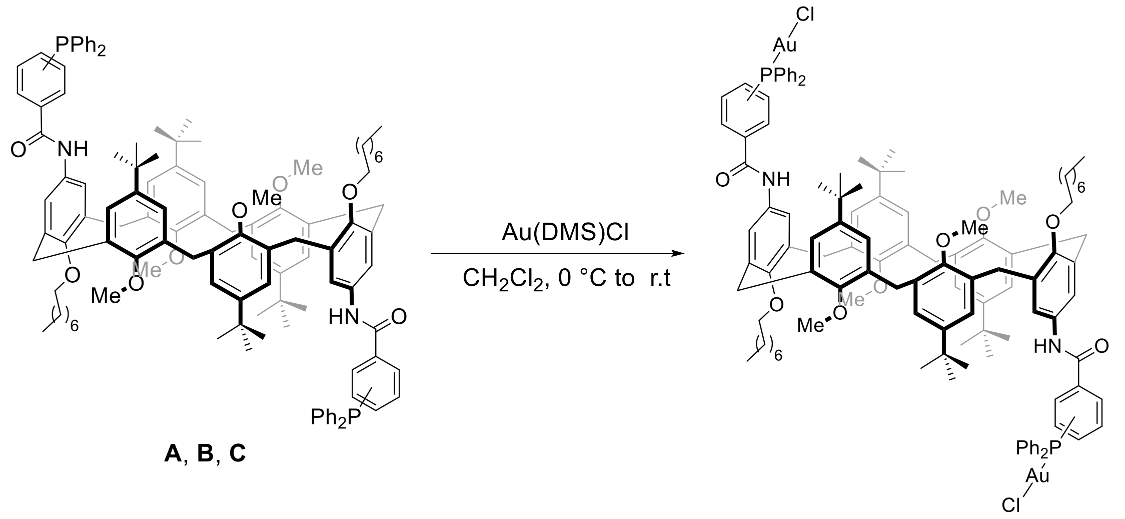

In a two-necked Schlenk flask, under N_2 atmosphere, Au(DMS)Cl (26.5 mg, 0.09 mmol, 2.0 equiv) was added to a solution of the corresponding phosphine (80 mg, 0.045 mmol, 1.0 equiv) in CH_2Cl_2 (4.0 mL) at 0 °C. The reaction mixture was stirred at the same temperature for 30 min and then allowed to reach room temperature. After 1 h, the mixture was filtered through celite, washed with CH_2Cl_2 (20 mL), and the volatiles were removed under vacuum. The crude was purified by column chromatography on silica gel (*n*-Hex/AcOEt 80:20→70:30).

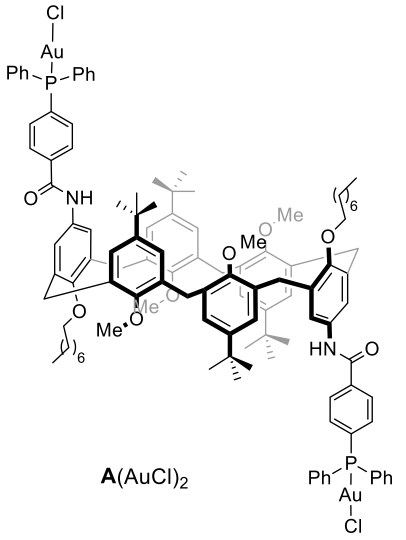

General procedure was followed using phosphine **A**. Purification by column chromatography on silica gel (*n*-Hex/EtOAc 80:20→70:30) yielded **A**(AuCl)$_2$ (92.1 mg, 93%) as a white solid. **M. p.** = 187-188 °C. **^{1}H NMR** *1,2,3-alternate conformer* (400 MHz, $CDCl_3$) δ = 7.83 – 7.76 (m, 4H), 7.71 – 7.62 (m, 4H), 7.61 – 7.47 (m, 22H), 7.18 (br s, 4H), 6.94 (br s, 4H), 6.86 (br s, 4H), 4.18 (d, *J* = 14.2 Hz, 4H), 3.91 (s, 4H), 3.88 (t, *J* = 6.7 Hz, 4H), 3.65 (d, *J* = 14.2Hz, 4H), 2.96 (br s, 12H), 1.90 – 1.79 (m, 4H), 1.58 – 1.47 (m, 4H), 1.37 – 1.23 (m, 16H), 1.15 (s, 36H), 0.93 – 0.80 (m, 6H). **^{13}C NMR** *1,2,3-alternate conformer* (101 MHz,

CDCl$_3$) δ = 163.9 (C$_q$), 154.0 (C$_q$), 151.8 (C$_q$), 146.3 (2 x C$_q$), 138.4 (d, J_{C-P} = 2.5 Hz, C$_q$), 135.3 (C$_q$), 134.4 (d, J_{C-P} = 13.8 Hz, CH), 134.2 (d, J_{C-P} = 13.8 Hz, CH); 134.0 (C$_q$), 133.4 (C$_q$), 132.6 (C$_q$), 132.3 (d, J_{C-P} = 2.2 Hz, CH), 129.4 (d, J_{C-P} = 12.2 Hz, CH), 128.1 (d, J_{C-P} = 61.9 Hz, C$_q$), 127.8 (d, J_{C-P} = 9.8 Hz, CH), 126.7 (CH), 126.6 (CH), 121.3 (CH), 73.4 (CH$_2$), 60.1 (CH$_3$), 34.1 (C$_q$), 31.9 (CH$_2$), 31.4 (CH$_3$), 30.8 (CH$_2$), 30.4 (2 x CH$_2$), 29.5 (CH$_2$), 29.4 (CH$_2$), 26.3 (CH$_2$), 22.7 (CH$_2$), 14.2 (CH$_3$). **^{31}P NMR** *1,2,3-alternate conformer* (162 MHz, CDCl$_3$) δ = 32.9. **HR-MS**: *m/z* [M-(AuCl$_2$)]$^+$ calcd. for $C_{116}H_{136}AuN_2O_8P_2$: 1944.9471; found: 1994.9465.

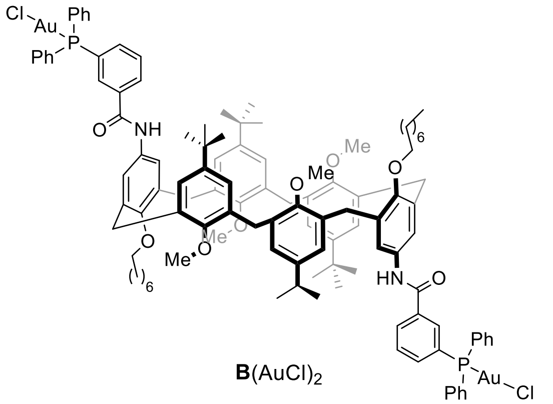

B(AuCl)$_2$

General procedure was followed using phosphine **B**. Purification by column chromatography on silica gel (*n*-Hex/EtOAc 80:20→70:30) yielded **B**(AuCl)$_2$ (73.5 mg, 74%) as a white solid. **M. p.** = 193-195 °C. **^{1}H NMR** *1,2,3-alternate conformer* (400 MHz, CDCl$_3$) δ = 7.90 – 7.80 (m, 4H), 7.77 – 7.64 (m, 4H), 7.61 – 7.39 (m, 21H), 7.17 (br s, 4H), 6.93 (br s, 3H), 6.84 (br s, 6H), 4.18 (d, *J* = 14.2 Hz, 4H), 3.97 – 3.86 (m, 8H), 3.67 (d, *J* = 14.2 Hz, 4H), 2.96 (br s, 12H), 2.00 – 1.86 (m, 4H), 1.67 – 1.53 (m, 4H), 1.42 – 1.27 (m, 16H), 1.13 (s, 36H), 0.95 – 0.86 (m, 6H). **^{13}C NMR** *1,2,3-alternate conformer* (101 MHz, CDCl$_3$) δ = 163.6 (C$_q$), 154.0 (C$_q$), 151.8 (C$_q$), 146.2 (C$_q$), 137.3 (d, J_{C-P} = 13.8 Hz, CH), 136.1 (d, J_{C-P} = 10.2 Hz, C$_q$), 135.3 (C$_q$), 134.2 (d, J_{C-P} = 13.8 Hz, CH), 133.9 (C$_q$), 132.8 (d, J_{C-P} = 12.8 Hz, CH), 132.6 (C$_q$), 132.3 (d, J_{C-P} =2.5 Hz, CH), 130.5 (C$_q$), 129.9 (C$_q$), 129.8 (CH), 129.6 (d, J_{C-P} = 12.2 Hz, CH), 129.4 (d, J_{C-P} = 12.2 Hz, CH), 128.1 (d, J_{C-P} = 62.5 Hz, C$_q$), 126.7 (CH), 126.3 (CH), 121.3 (CH), 73.3 (CH$_2$), 60.1 (CH$_3$), 34.1 (C$_q$), 31.9 (CH$_2$), 31.4 (CH$_3$), 30.8 (CH$_2$), 30.6 (2 x CH$_2$), 29.6 (CH$_2$), 29.3 (CH$_2$), 26.3 (CH$_2$), 22.7 (CH$_2$), 14.1 (CH$_3$). **^{31}P NMR** *1,2,3-alternate conformer* (162 MHz, CDCl$_3$) δ = 33.3. **HR-MS**: *m/z* [M-Cl]$^+$ calcd. for $C_{116}H_{136}Au_2ClN_2O_8P_2$: 2176.8825; found: 2176.8828.

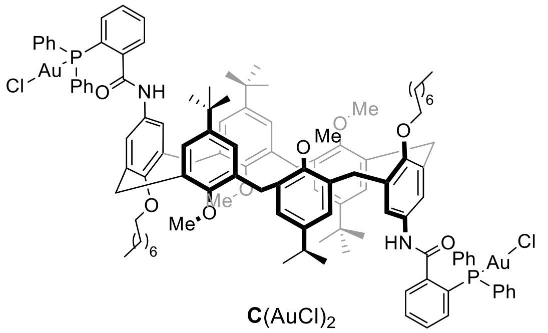

C(AuCl)$_2$

General procedure was followed using phosphine **C**. Purification by column chromatography on silica gel (*n*-Hex/EtOAc 80:20→70:30) yielded **C**(AuCl)$_2$ (68.7 mg, 69%) as a white solid. **M. p.** = 196-197 °C. **^{1}H NMR** *1,2,3-alternate conformer* (400 MHz, $CDCl_3$) δ = 7.62 – 7.58 (m, 4H), 7.58 – 7.50 (m, 9H), 7.48 – 7.41 (m, 6H), 7.40 – 7.34 (m, 9H), 7.33 – 7.30 (m, 4H), 7.25 – 7.27 (m, 2H), 7.15 (br s, 2H), 6.93 – 6.85 (m, 6H), 4.30 (d, *J* = 14.2 Hz, 4H), 3.92 – 3.84 (m, 8H), 3.58 (d, *J* = 14.2 Hz, 4H), 2.91 (br s, 12H), 1.97 – 1.84 (m, 4H), 1.64 – 1.52 (m, 4H), 1.44 – 1.28 (m, 16H), 1.21 (s, 36H), 0.95 – 0.87 (m, 6H). **^{13}C NMR** *1,2,3-alternate conformer* (101 MHz, $CDCl_3$) δ = 165.2 (C_q), 154.2 (C_q), 152.3 (C_q), 145.8 (C_q), 142.0 (d, $J_{C\text{-}P}$ = 10.8 Hz, C_q), 135.3 (d, $J_{C\text{-}P}$ = 9.2 Hz, CH), 135.1 (C_q), 134.2 (d, $J_{C\text{-}P}$ = 13.9 Hz, CH), 133.6 (d, $J_{C\text{-}P}$ = 5.1 Hz, C_q), 132.0 (C_q), 131.7 (CH), 131.6 (d, $J_{C\text{-}P}$ = 2.6 Hz, CH), 130.3 (d, $J_{C\text{-}P}$ = 10.4 Hz, CH), 129.7 (C_q), 129.0 (d, $J_{C\text{-}P}$ = 13.0 Hz, CH), 128.4 (d, $J_{C\text{-}P}$ = 8.9 Hz, C_q), 128.0 (d, $J_{C\text{-}P}$ = 58.3 Hz, C_q), 127.1 (CH), 126.2 (CH), 122.5 (CH), 121.2 (CH), 73.7 (CH_2), 59.9 (CH_3), 34.1 (C_q), 31.9 (CH_2), 31.5 (CH_3), 30.9 (CH_2), 30.6 (2 x CH_2), 29.6 (CH_2), 29.3 (CH_2), 26.3 (CH_2), 22.7 (CH_2), 14.1 (CH_3). **^{31}P NMR** *1,2,3-alternate conformer* (162 MHz, $CDCl_3$) δ = 34.6. **HR-MS**: *m/z* [M-Cl]$^+$ calcd. for $C_{116}H_{136}Au_2ClN_2O_8P_2$: 2176.8825; found: 2176.8819.

- **Synthesis and Characterization of monomeric Au(I) catalysts A’,B’,C’(AuCl)**

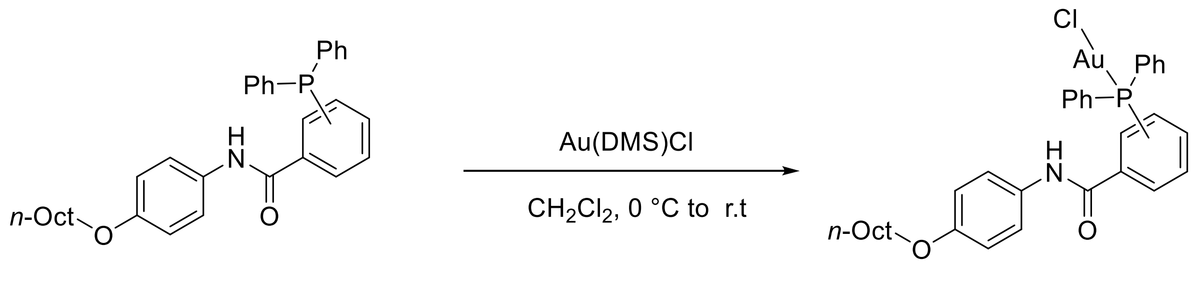

In a two-necked Schlenk flask, under N_2 atmosphere, Au(DMS)Cl (29.4 mg, 0.1 mmol, 1.0 equiv) was added to a solution of the corresponding phosphine (50.9 mg, 0.1 mmol, 1.0 equiv) in CH_2Cl_2 (4.0 mL) at 0 °C. The reaction mixture was stirred at the same temperature for 30 min and then allowed to reach room temperature. After 1 h, the mixture was filtered through celite, washed with CH_2Cl_2 (20 mL), and the volatiles were removed under vacuum. The crude was purified by column chromatography on silica gel (*n*-Hex/AcOEt: 80:20→70:30).

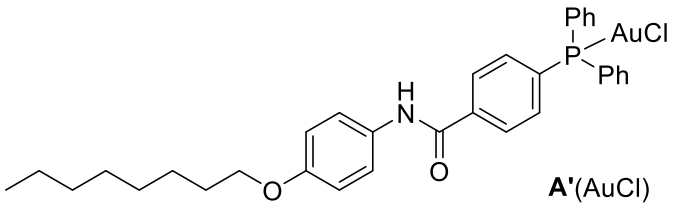

A'(AuCl)

General procedure was followed using **A'**. Purification by column chromatography on silica gel (*n*-Hex/EtOAc 80:20→70:30) yielded **A'**(AuCl) (69.8 mg, 94%) as a white solid. **M. p.** = 93-94 °C. **^{1}H NMR** (400 MHz, $CDCl_3$) δ = 8.08 (s, 1H), 7.95 (dd, J = 8.3, 2.2 Hz, 2H), 7.66 – 7.44 (m, 14H), 6.97 – 6.84 (m, 2H), 3.96 (t, J = 6.6 Hz, 2H), 1.89 – 1.71 (m, 2H), 1.46 (q, J = 7.1 Hz, 2H), 1.42 – 1.24 (m, 8H), 1.00 – 0.84 (m, 3H). **^{13}C NMR** (101 MHz, $CDCl_3$) δ = 164.4 (C_q), 156.5 (C_q), 138.3 (C_q), 134.3 (d, J_{C-P} = 13.7 Hz, CH), 134.2 (d, J_{C-P} = 14.0 Hz, CH), 133.0 (C_q), 132.3 (d, J_{C-P} = 2.6 Hz, CH), 130.4 (C_q), 129.5 (d, J_{C-P} = 11.6 Hz, CH), 127.9 (d, J_{C-P} = 61.9 Hz, C_q), 127.7 (d, J_{C-P} = 12.2 Hz, CH), 122.2 (CH), 114.9 (CH), 68.4 (CH_2), 31.8 (CH_2), 29.4 (CH_2), 29.3 (CH_2), 29.3 (CH_2), 26.0 (CH_2), 22.7 (CH_2), 14.1 (CH_3). **^{31}P NMR** (162 MHz, $CDCl_3$) δ = 34.3. **ESI-MS**: *m/z* $[M-Cl]^+$ calcd. for $C_{33}H_{36}AuNO_2P$: 706.21; found: 706.18.

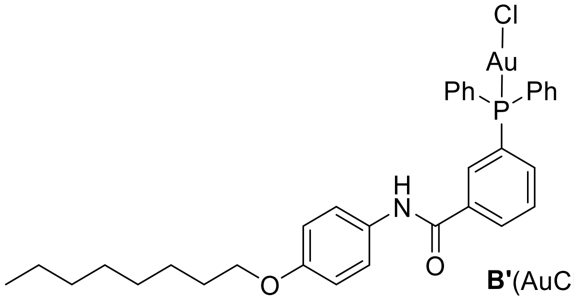

B'(AuCl)

General procedure was followed using **B'**. Purification by column chromatography on silica gel (*n*-Hex/EtOAc 80:20→70:30) yielded **B'**(AuCl) (68.5 mg, 92%) as a white solid. **M. p.** = 91-92 °C. **^{1}H NMR** (400 MHz, $CDCl_3$) δ = 8.06 – 7.96 (m, 3H), 7.72 – 7.38 (m, 14H), 6.96 – 6.79 (m, 2H), 3.95 (t, J = 6.6 Hz, 2H), 1.89 – 1.72 (m, 2H), 1.46 (q, J = 7.3 Hz, 2H), 1.41 – 1.22 (m, 8H), 0.99 – 0.82 (m, 3H). **^{13}C NMR** (101 MHz, $CDCl_3$) δ = 164.3 (C_q), 156.5 (C_q), 136.7 (d, J_{C-P} = 13.7 Hz, CH), 136.3 (d, J_{C-P} = 10.9 Hz, C_q), 134.3 (d, J_{C-P} = 13.9 Hz, CH), 132.4 (d, J_{C-P} = 2.6 Hz, CH), 132.2 (d, J_{C-P} = 14.0 Hz, CH), 130.7 (CH), 130.3 (C_q), 129.9 (C_q), 129.7 (d, J_{C-P} = 11.8 Hz, CH), 129.5 (d, J_{C-P} = 12.0 Hz, CH), 128.0 (d, J_{C-P} = 62.0 Hz, C_q), 122.3 (CH), 114.9 (CH), 68.3 (CH_2), 31.8 (CH_2), 29.4 (CH_2), 29.3 (CH_2), 29.3 (CH_2), 26.1 (CH_2), 22.7 (CH_2), 14.1 (CH_3). **^{31}P NMR** (162 MHz, $CDCl_3$) δ = 43.2. **ESI-MS**: *m/z* $[M-Cl]^+$ calcd. for $C_{33}H_{36}AuNO_2P$: 706.21; found: 706.17.

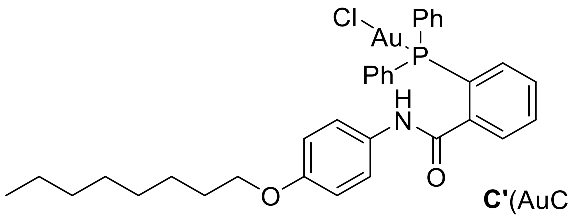

C'(AuCl)

General procedure was followed using **C'**. Purification by column chromatography on silica gel (*n*-Hex/EtOAc 80:20→70:30) yielded **C'**(AuCl) (71.0 mg, 96%) as a white solid. **M. p.** = 97-98 °C. **^{1}H NMR** (400 MHz, $CDCl_3$) δ = 7.89 (s, 1H), 7.86 (dd, *J* = 7.7, 4.2 Hz, 1H), 7.65 (t, *J* = 7.6 Hz, 1H), 7.59 – 7.40 (m, 12H), 7.34 (d, *J* = 8.5 Hz, 2H), 7.08 (dd, *J* = 12.5, 7.8 Hz, 1H), 6.75 (d, *J* = 8.9 Hz, 2H), 3.88 (t, *J* = 6.6 Hz, 2H), 1.74 (q, *J* = 6.9 Hz, 2H), 1.48 – 1.40 (m, 2H), 1.39 – 1.24 (m, 8H), 1.02 – 0.84 (m, 3H). **^{13}C NMR** (101 MHz, $CDCl_3$) δ = 165.6 (C_q), 156.7 (C_q), 141.1 (d, J_{C-P} = 11.6 Hz, C_q), 134.7 (d, J_{C-P} = 8.0 Hz, CH), 134.3 (d, J_{C-P} = 14.2 Hz, CH), 131.9 (CH), 131.7 (d, J_{C-P} = 2.5 Hz, CH), 130.6 (d, J_{C-P} = 9.4 Hz, CH), 129.7 (C_q), 129.4 (C_q), 129.1 (d, J_{C-P} = 12.2 Hz, CH), 128.9 (d, J_{C-P} = 8.1 Hz, CH), 128.3 (d, J_{C-P} = 57.9 Hz, C_q) 123.5 (CH), 114.6 (CH), 68.2 (CH_2), 31.8 (CH_2), 29.4 (CH_2), 29.3 (CH_2), 29.3 (CH_2), 26.1 (CH_2), 22.7 (CH_2), 14.1 (CH_3). **^{31}P NMR** (162 MHz, $CDCl_3$) δ = 34.5. **ESI-MS**: *m/z* $[M-Cl]^+$ calcd. for $C_{33}H_{36}$ $AuNO_2P$: 706.21; found: 706.23.

- **NMR Spectra**

Several NMR spectra are presented for each novel compound in the following order: ^{1}H, ^{13}C-APT; ^{31}P and, if present, edited HSQC and g-COSY

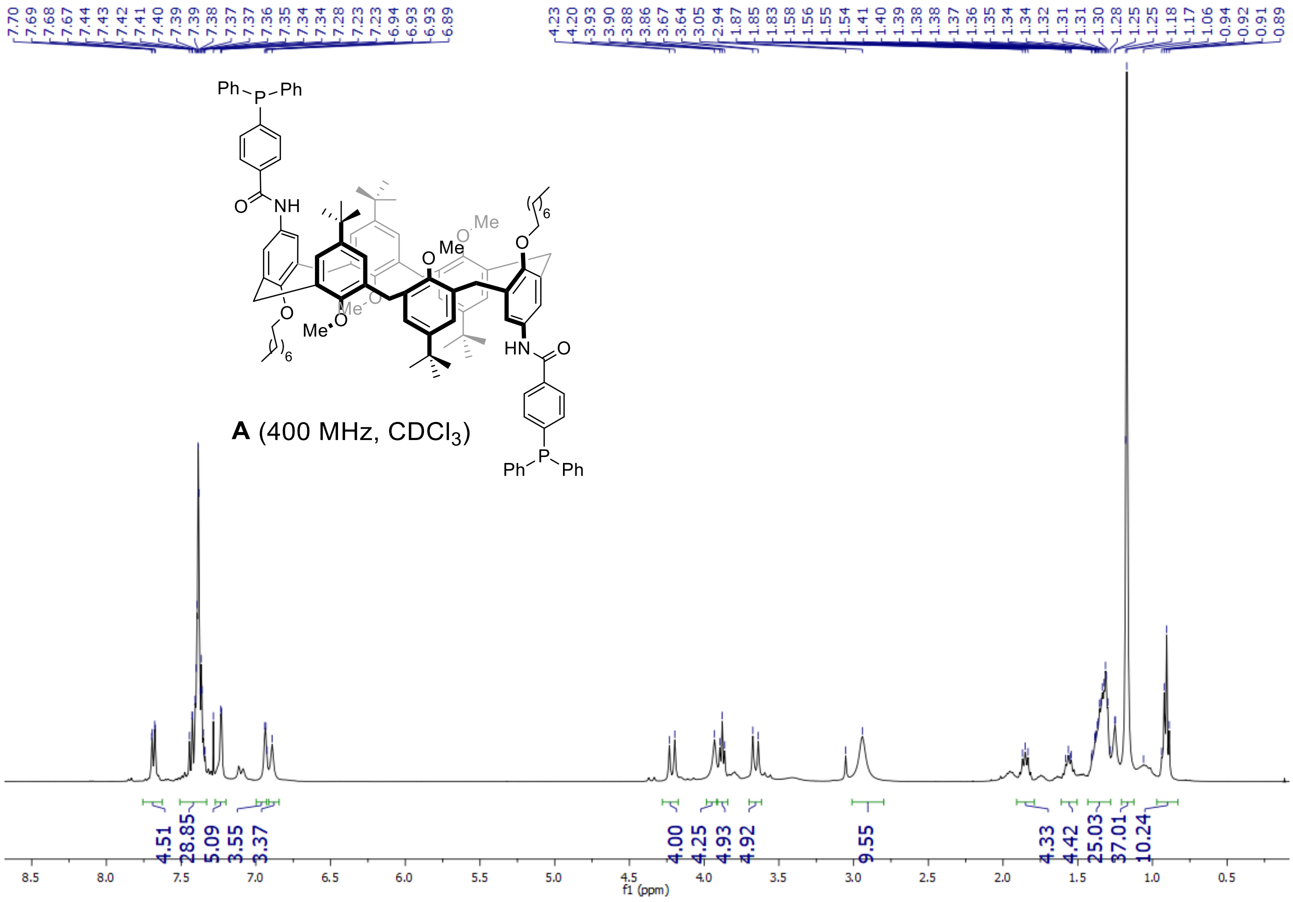

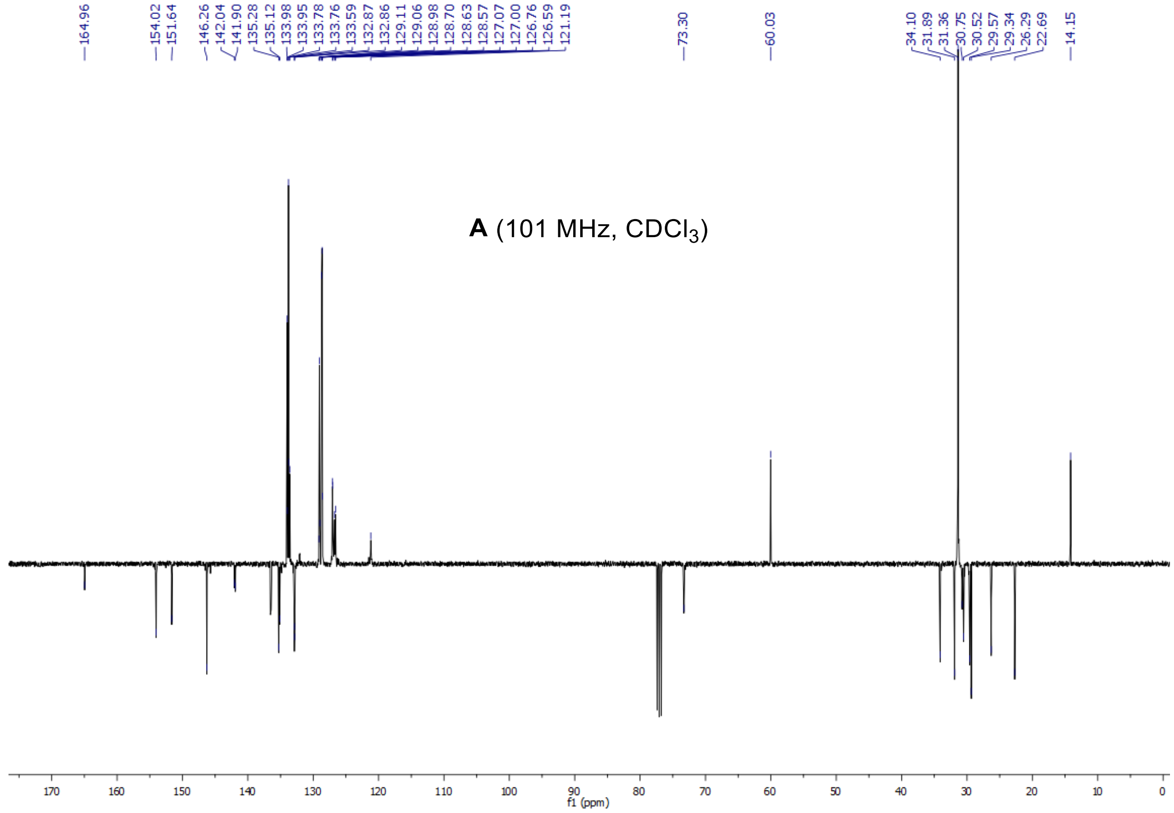
A (101 MHz, CDCl3)
f1 (ppm)

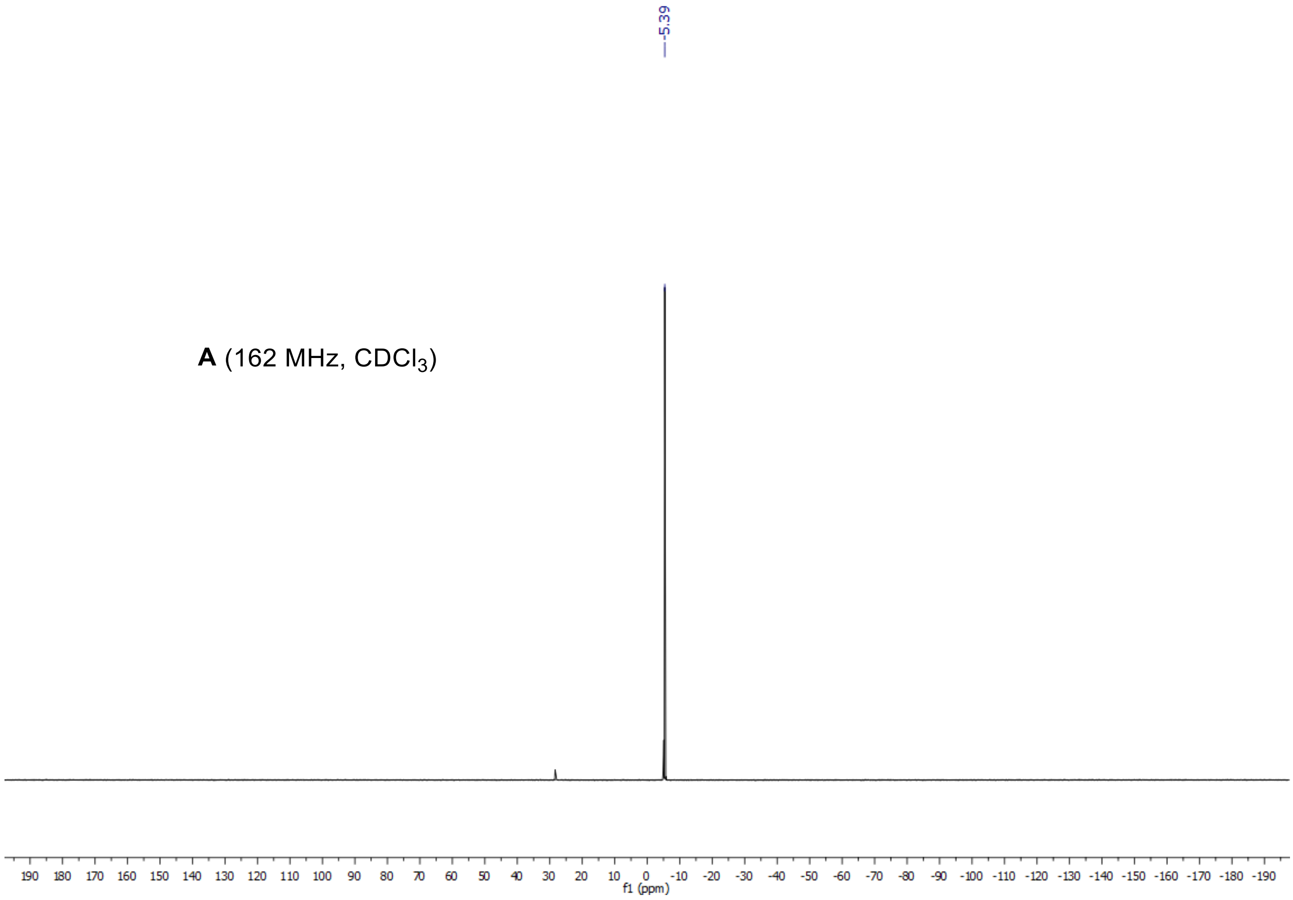
A (162 MHz, CDCl3)
f1 (ppm)

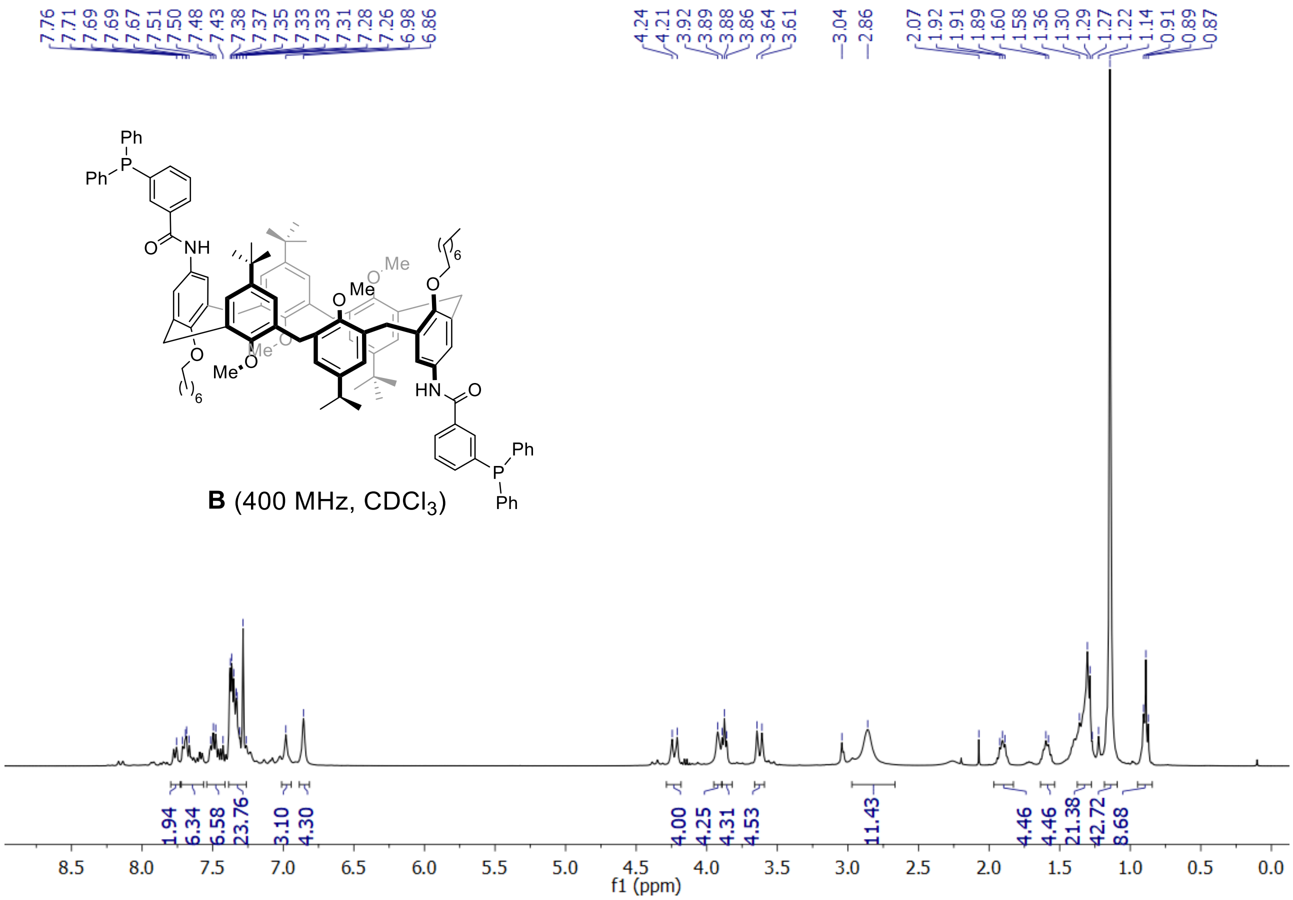
7.76
7.71
7.69
7.69
7.67
7.51
7.50
7.48
7.43
7.38
7.37
7.35
7.33
7.33
7.31
7.28
7.26
6.98
6.86
4.24
4.21
3.92
3.89
3.88
3.86
3.64
3.61
3.04
2.86
2.07
1.92
1.91
1.89
1.60
1.58
1.36
1.30
1.29
1.27
1.22
1.14
0.91
0.89
0.87
Ph
P
Ph
NH
O
Me
O
Me
O
6
Me
O
Me
O
6
HN
O
Ph
P
Ph
B (400 MHz, CDCl3)
1.94
6.34
6.58
23.76
3.10
4.30
4.00
4.25
4.31
4.53
11.43
4.46
4.46
21.38
42.72
8.68
8.5
8.0
7.5
7.0
6.5
6.0
5.5
5.0
4.5
4.0
3.5
3.0
2.5
2.0
1.5
1.0
0.5
0.0
f1 (ppm)

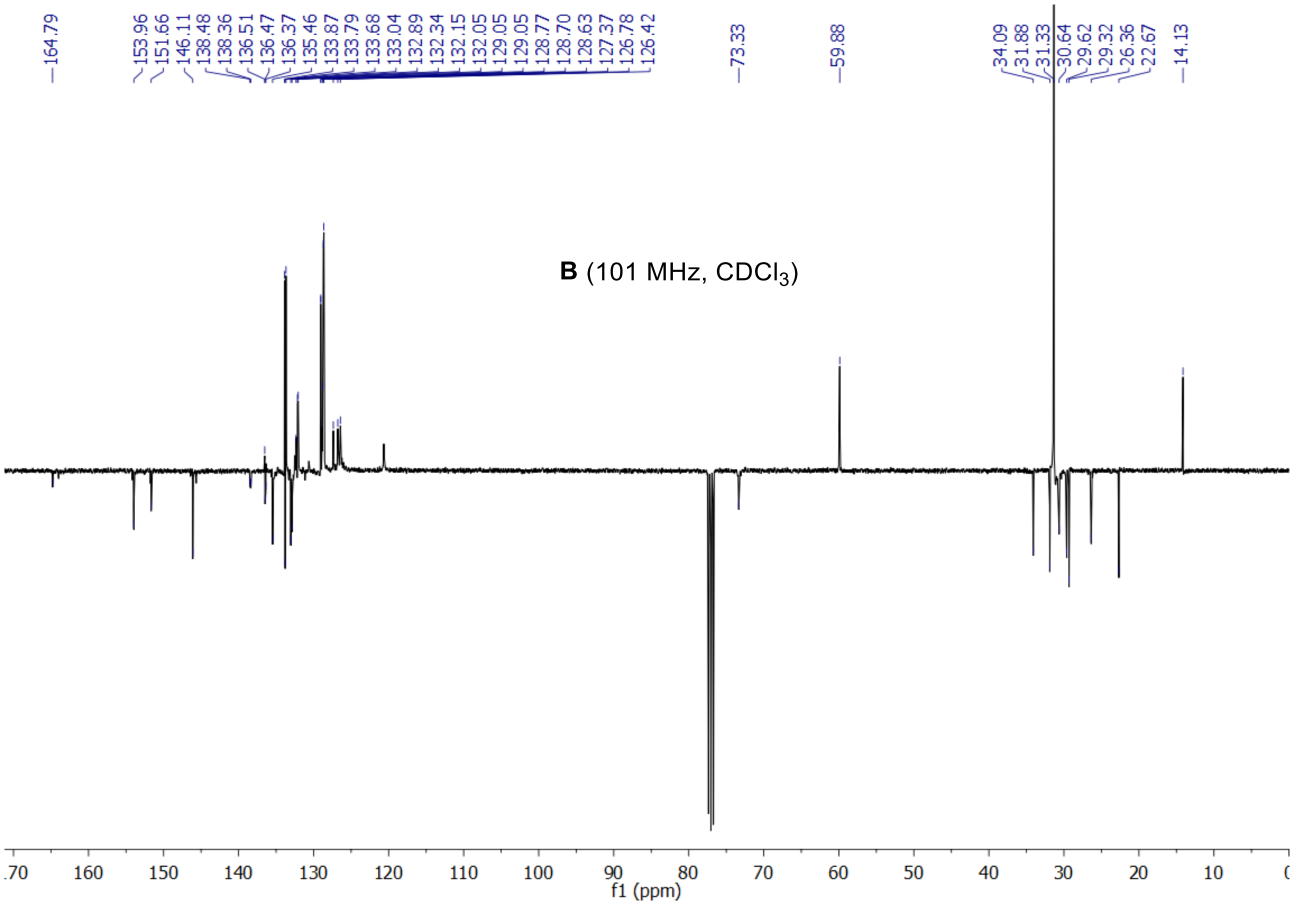
164.79
153.96
151.66
146.11
138.48
138.36
136.51
136.47
136.37
135.46
133.87
133.79
133.68
133.04
132.89
132.34
132.15
132.05
129.05
129.05
128.77
128.70
128.63
127.37
126.78
126.42
73.33
59.88
34.09
31.88
31.33
30.64
29.62
29.32
26.36
22.67
14.13
B (101 MHz, CDCl3)
f1 (ppm)

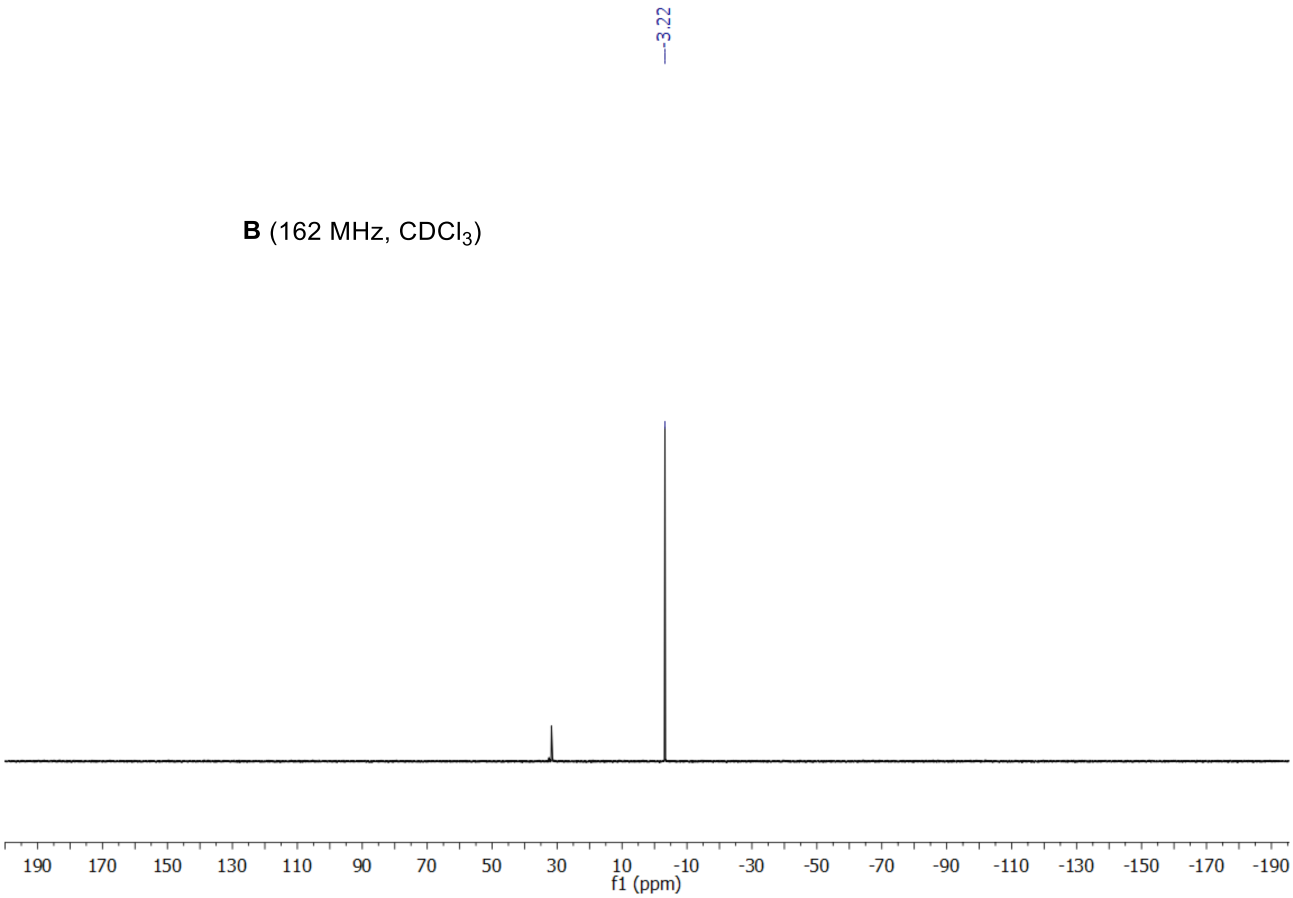
3.22
B (162 MHz, CDCl3)
f1 (ppm)

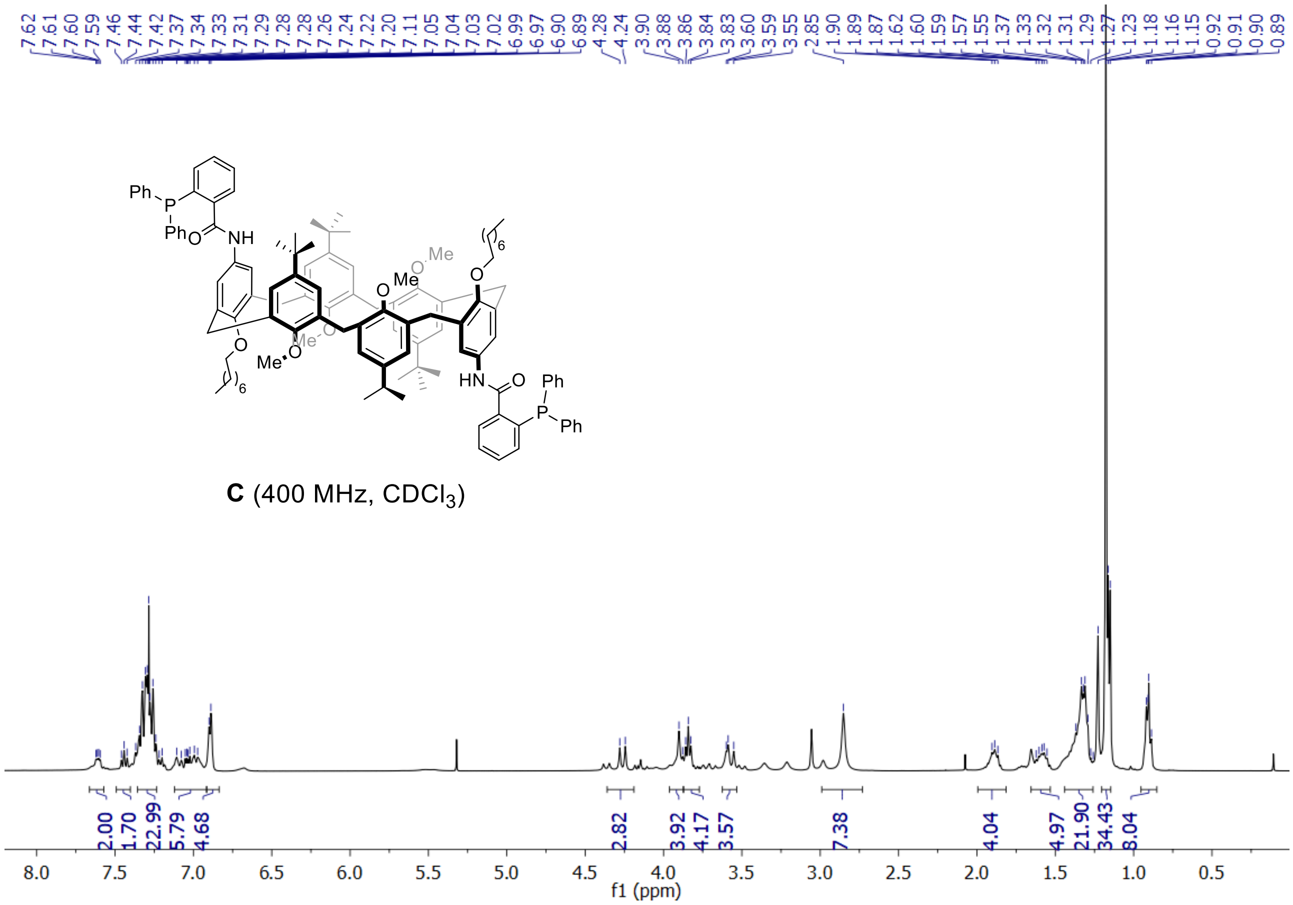
7.62 7.61 7.60 7.59 7.46 7.44 7.42 7.37 7.34 7.33 7.31 7.29 7.28 7.26 7.24 7.22 7.20 7.11 7.05 7.04 7.03 7.02 6.99 6.97 6.90 6.89 4.28 4.24 3.90 3.88 3.86 3.84 3.83 3.60 3.59 3.55 2.85 1.90 1.89 1.87 1.62 1.60 1.59 1.57 1.55 1.37 1.33 1.32 1.31 1.29 1.27 1.23 1.18 1.16 1.15 0.92 0.91 0.90 0.89
Ph
P
Ph
O
NH
O
Me
Me
O
O
Me
O
6
O
Me
O
Me
O
6
HN
O
Ph
P
Ph
C (400 MHz, $CDCl_3$)
2.00 1.70 22.99 5.79 4.68 2.82 3.92 4.17 3.57 7.38 4.04 4.97 21.90 34.43 8.04
8.0 7.5 7.0 6.5 6.0 5.5 5.0 4.5 4.0 3.5 3.0 2.5 2.0 1.5 1.0 0.5
f1 (ppm)

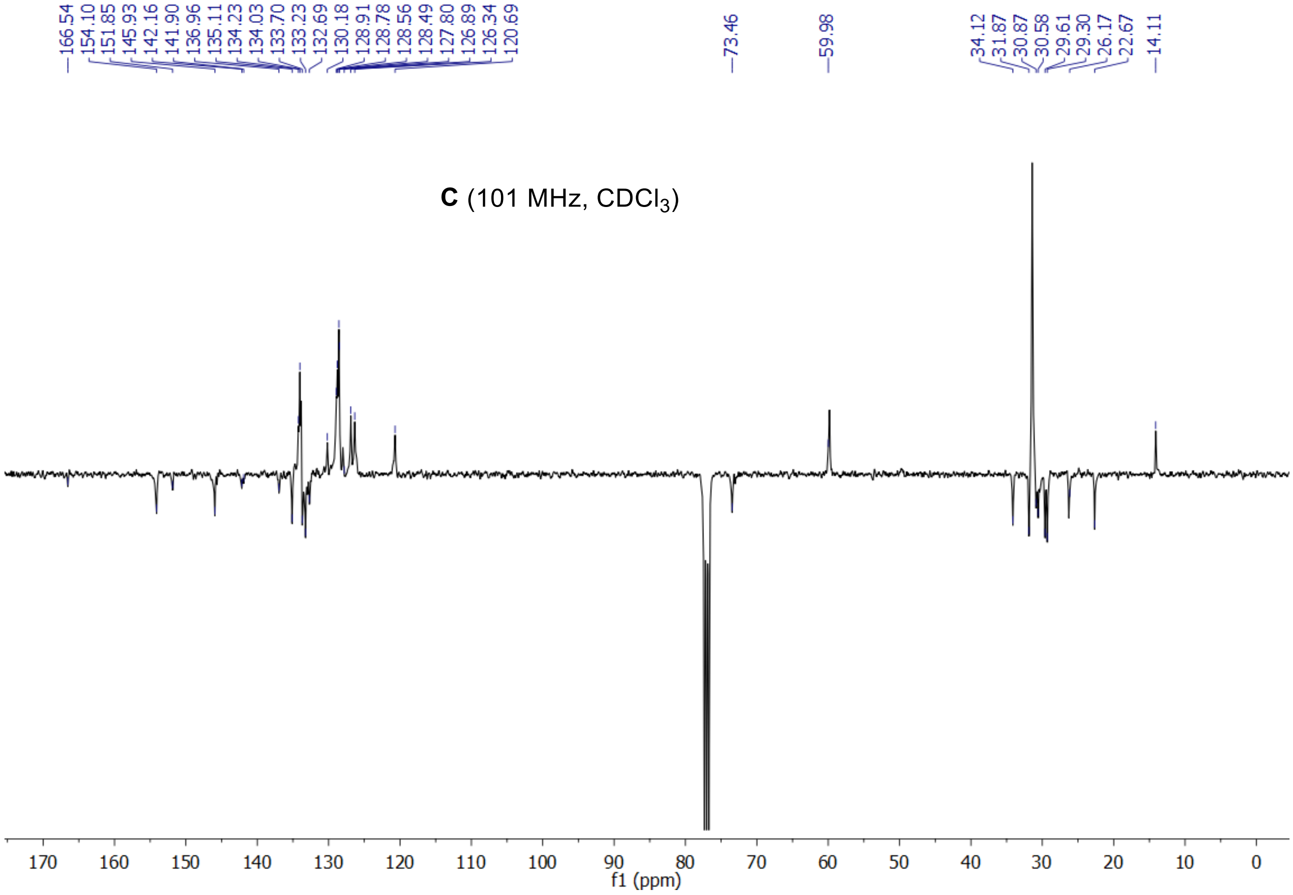
166.54 154.10 151.85 145.93 142.16 141.90 136.96 135.11 134.23 134.03 133.70 133.23 132.69 130.18 128.91 128.78 128.56 128.49 127.80 126.89 126.34 120.69
73.46
59.98
34.12 31.87 30.87 30.58 29.61 29.30 26.17 22.67
14.11
C (101 MHz, $CDCl_3$)
170 160 150 140 130 120 110 100 90 80 70 60 50 40 30 20 10 0
f1 (ppm)

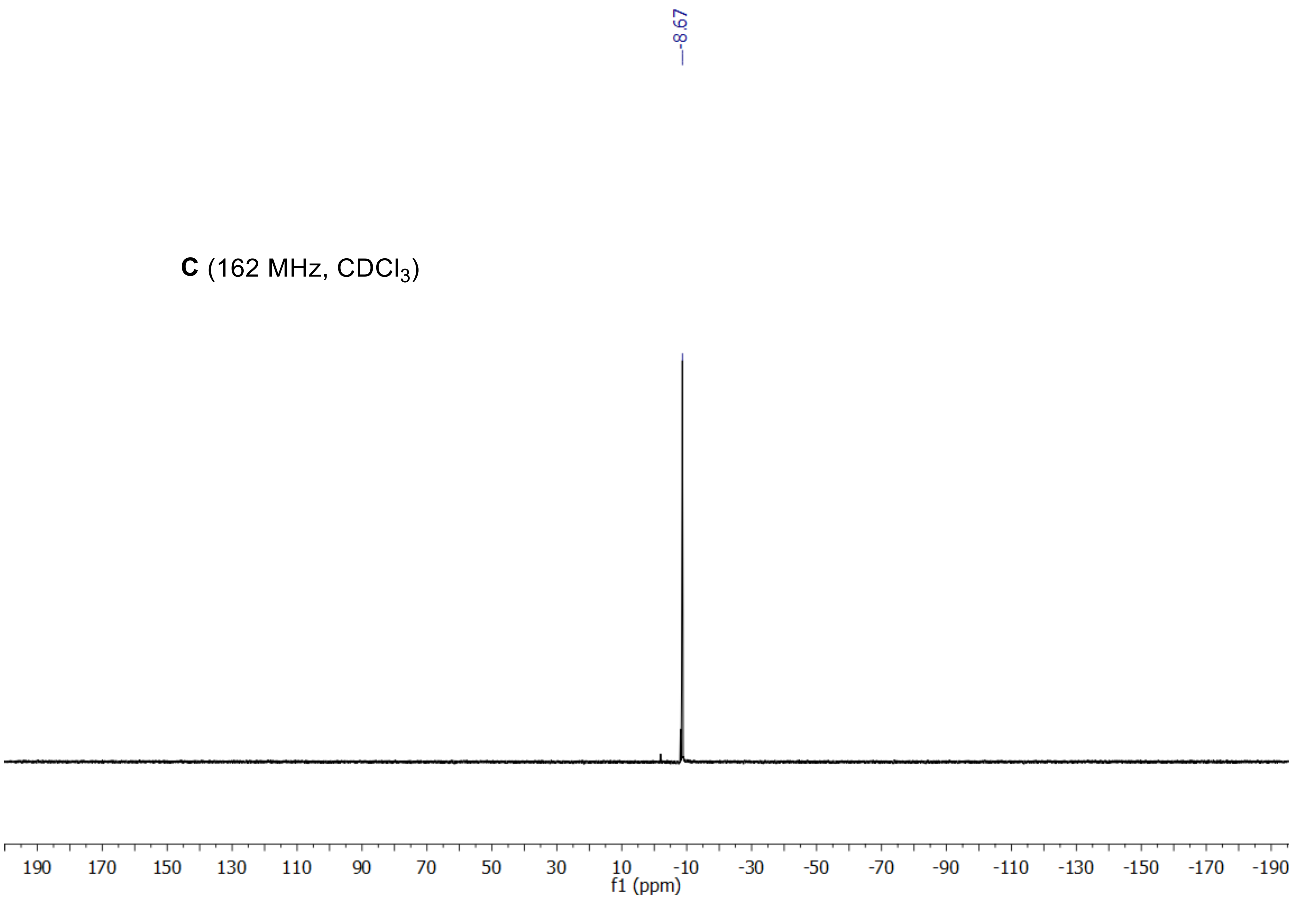
-8.67
C (162 MHz, CDCl3)
190 170 150 130 110 90 70 50 30 10 -10 -30 -50 -70 -90 -110 -130 -150 -170 -190
f1 (ppm)

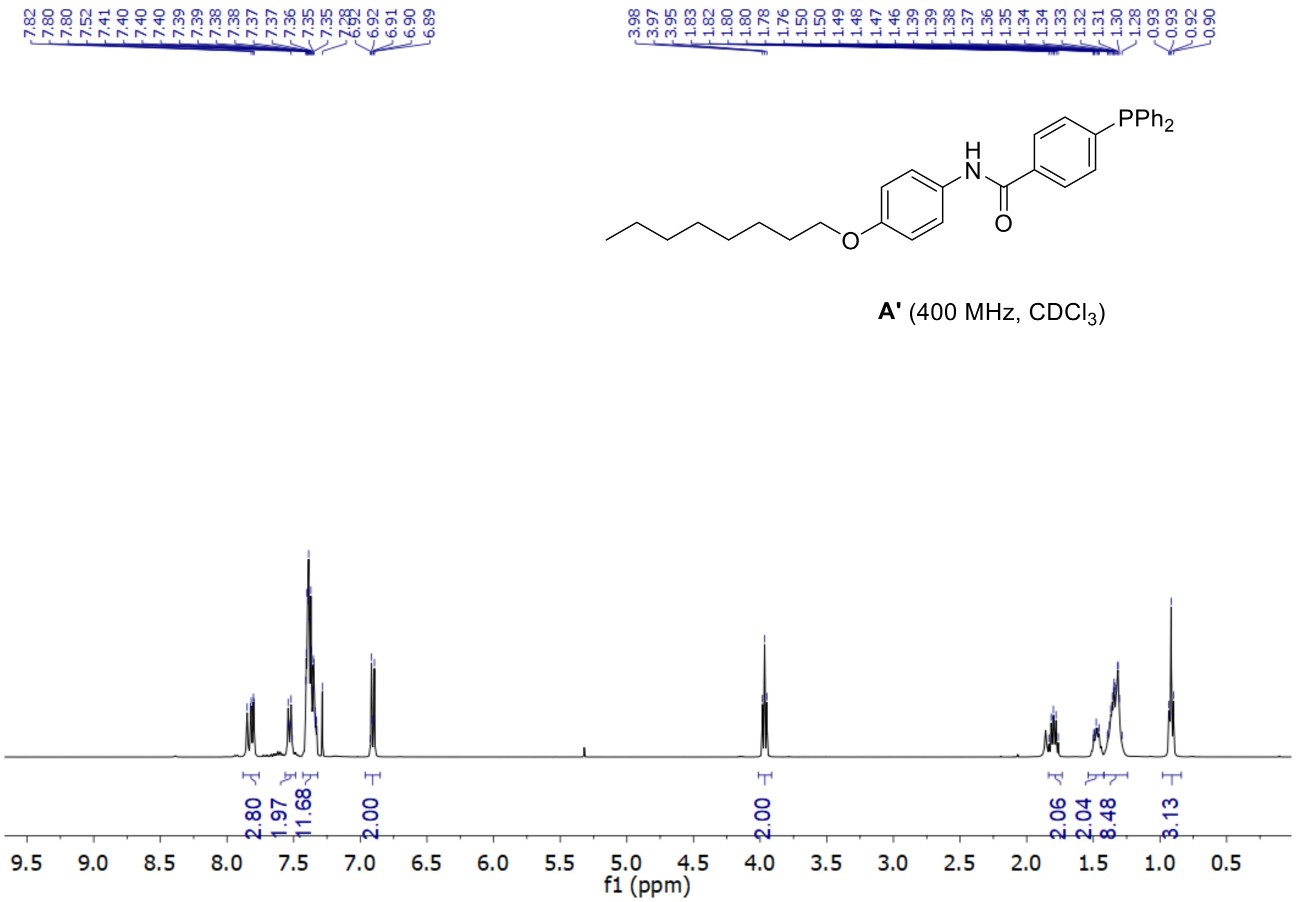

A' (400 MHz, $CDCl_3$)

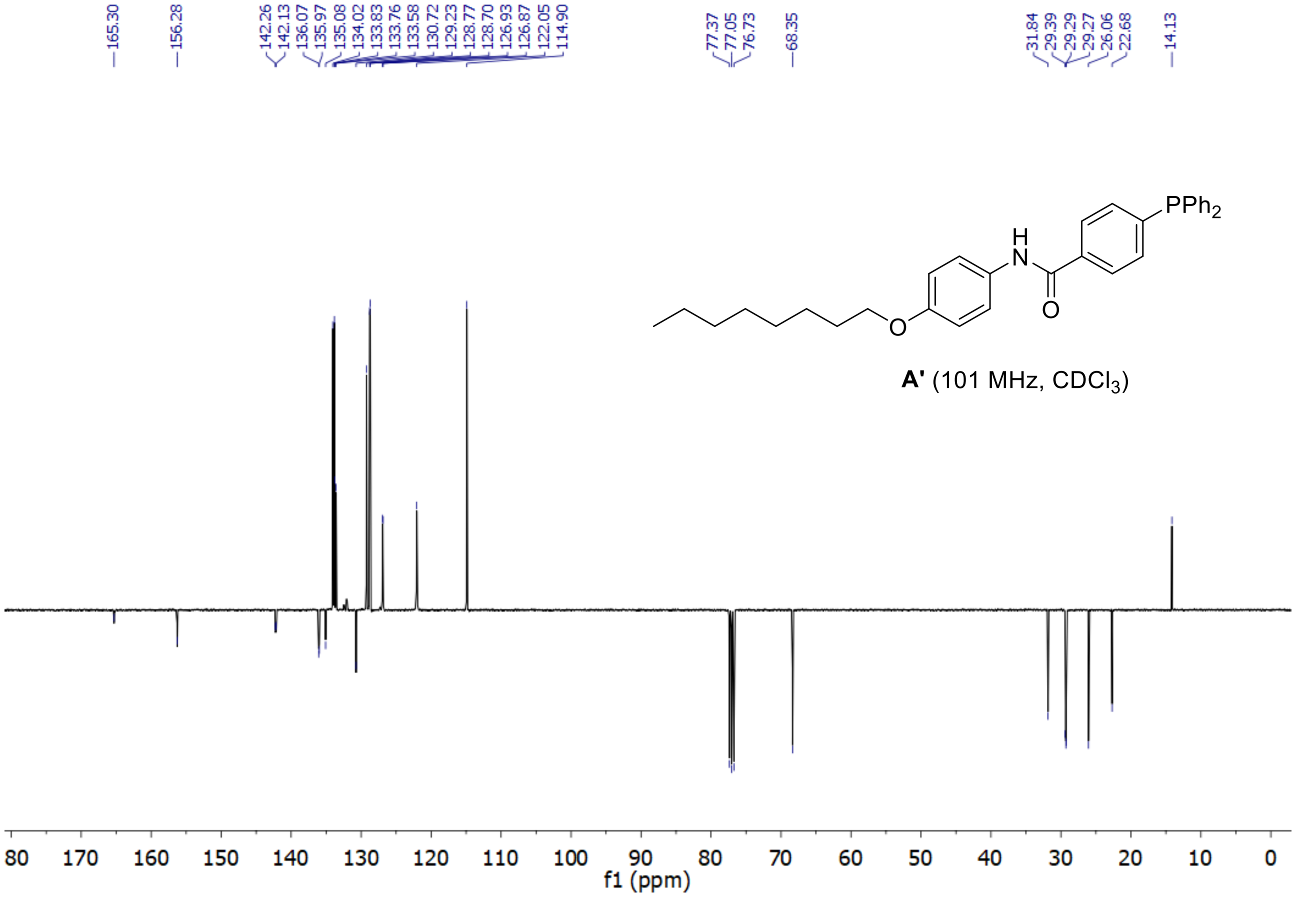

A' (101 MHz, $CDCl_3$)

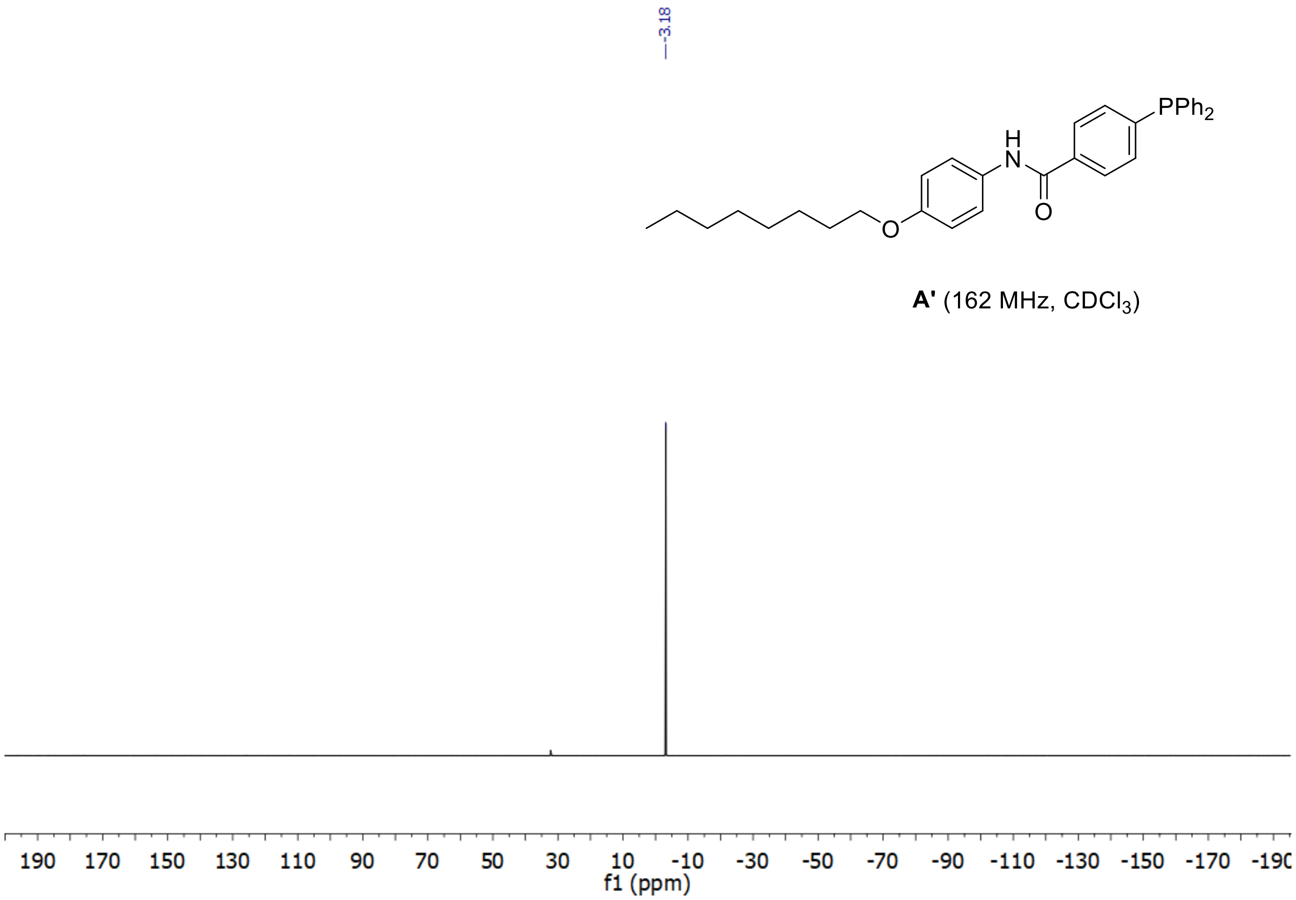

A' (162 MHz, $CDCl_3$)

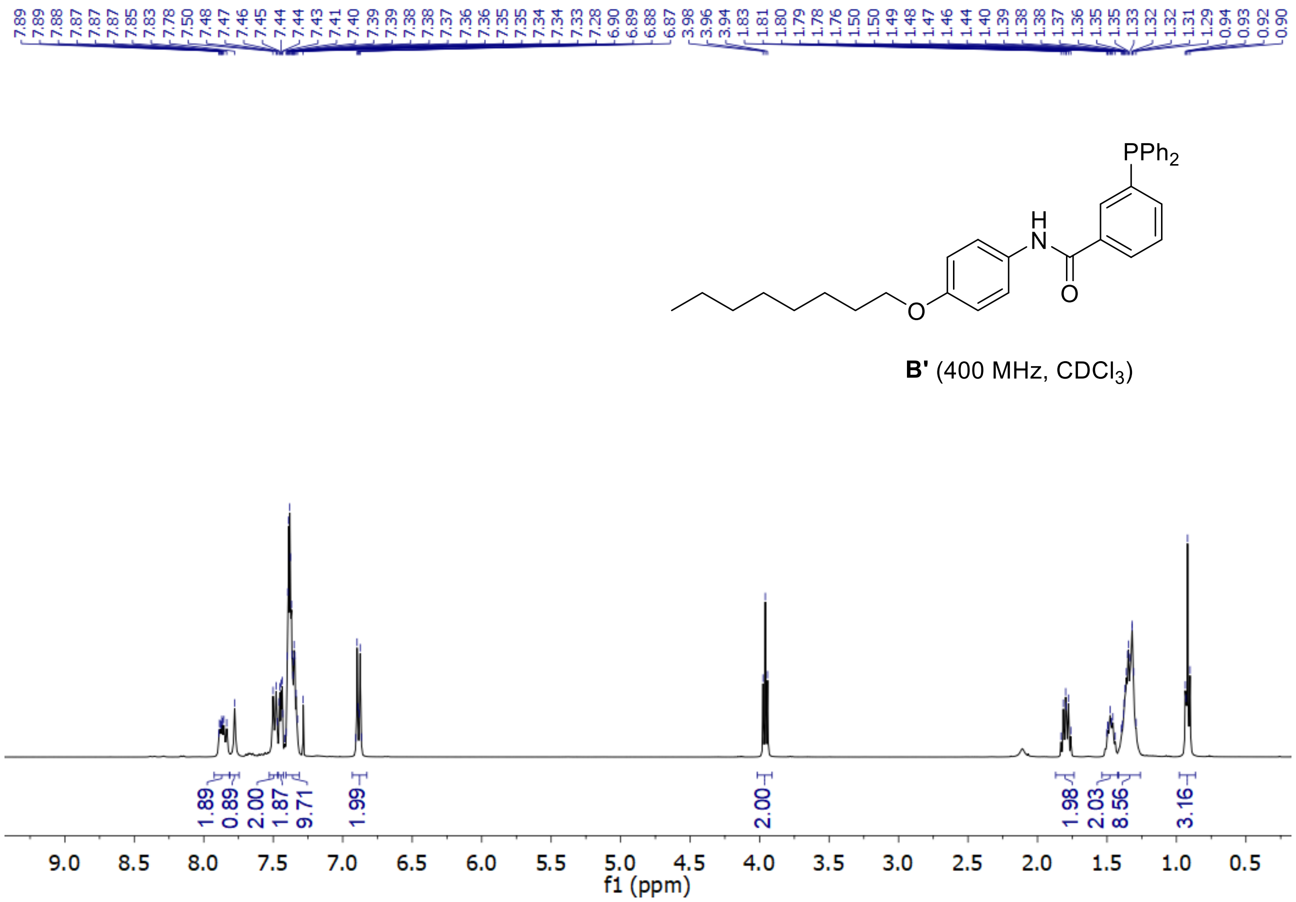

B' (400 MHz, $CDCl_3$)

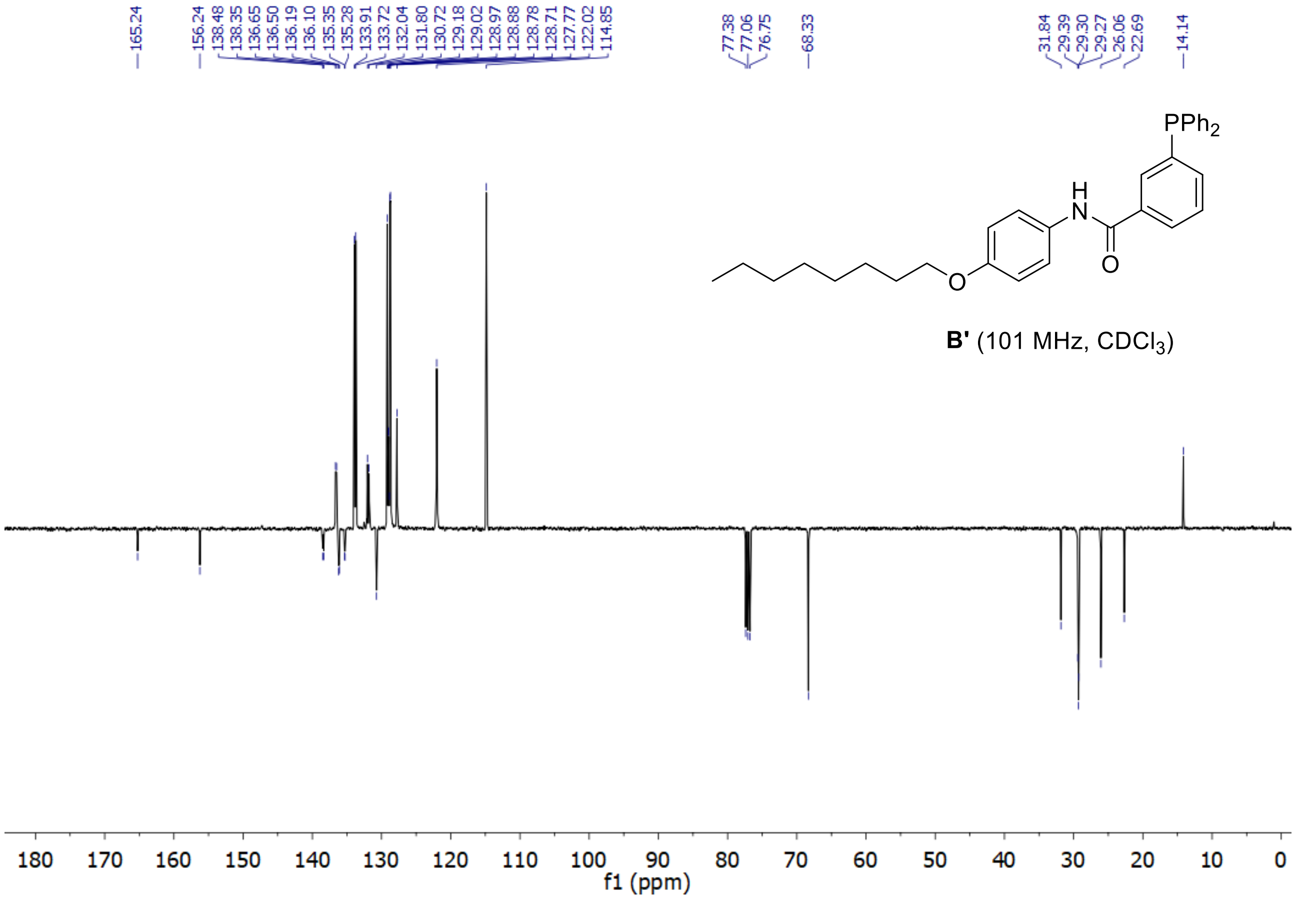

B' (101 MHz, $CDCl_3$)

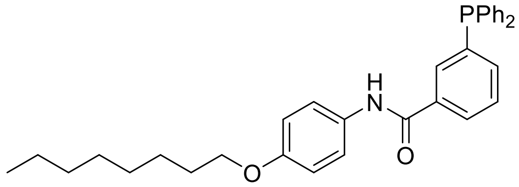

B' (162 MHz, $CDCl_3$)

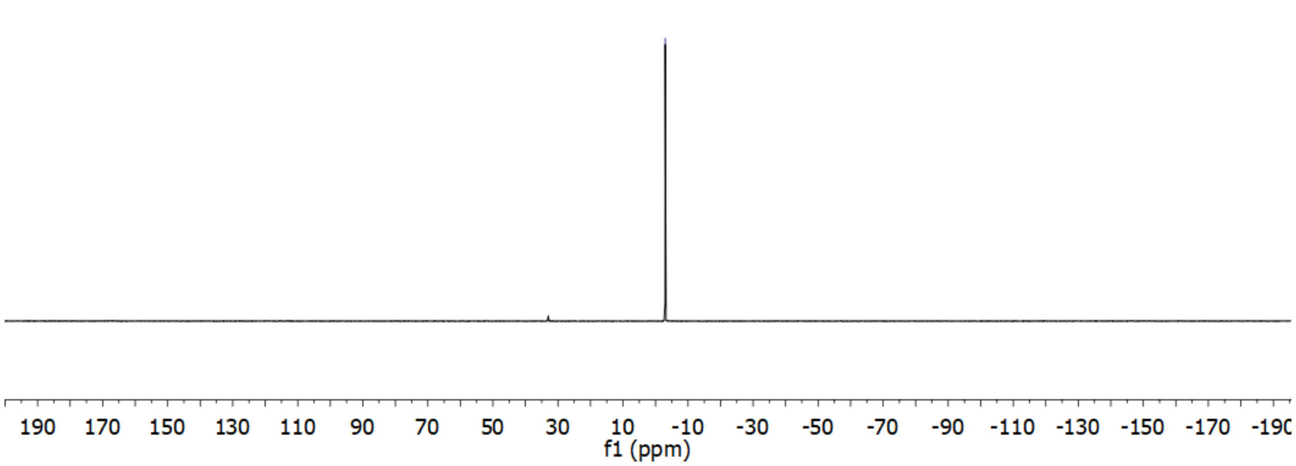

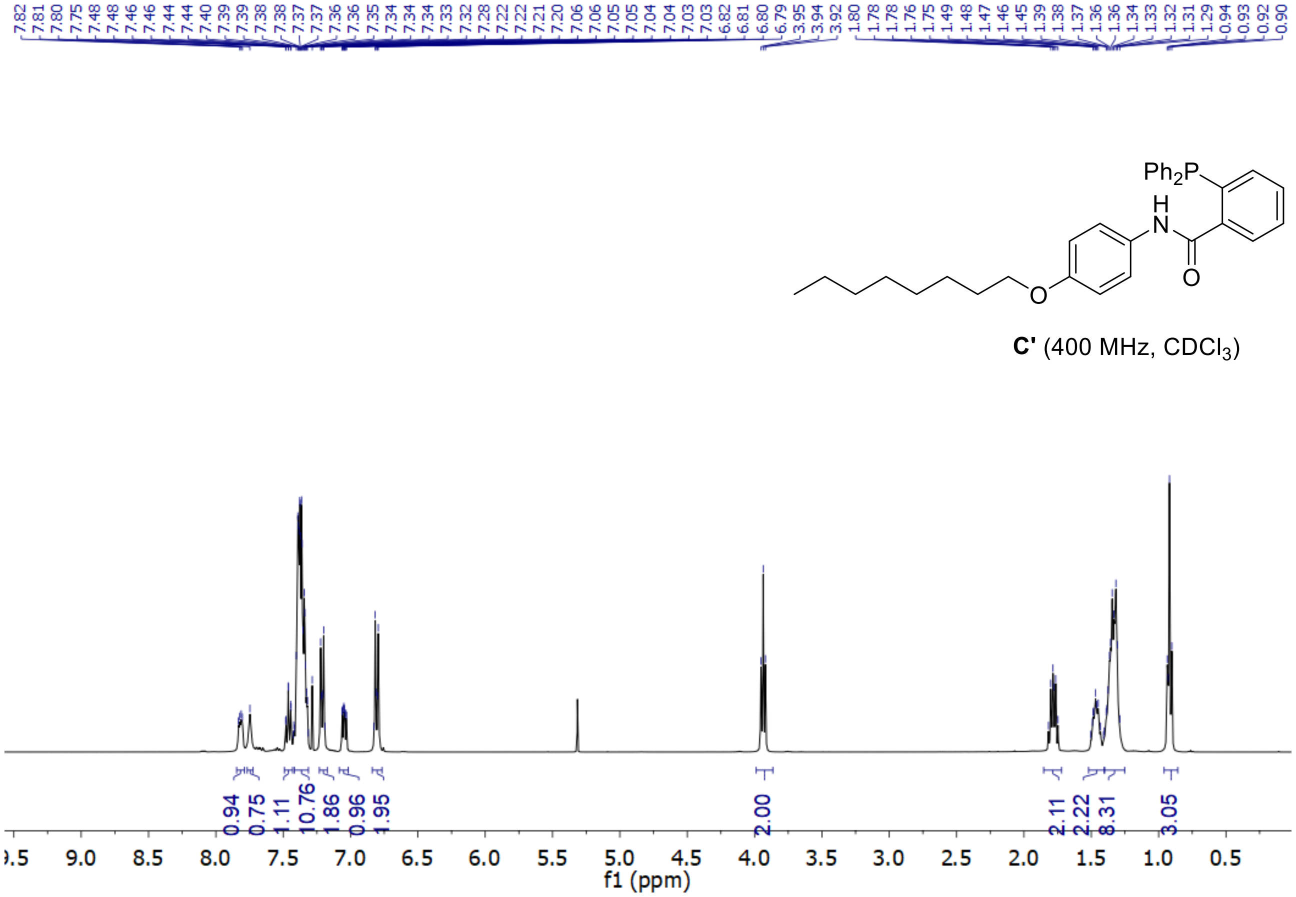

C' (400 MHz, $CDCl_3$)

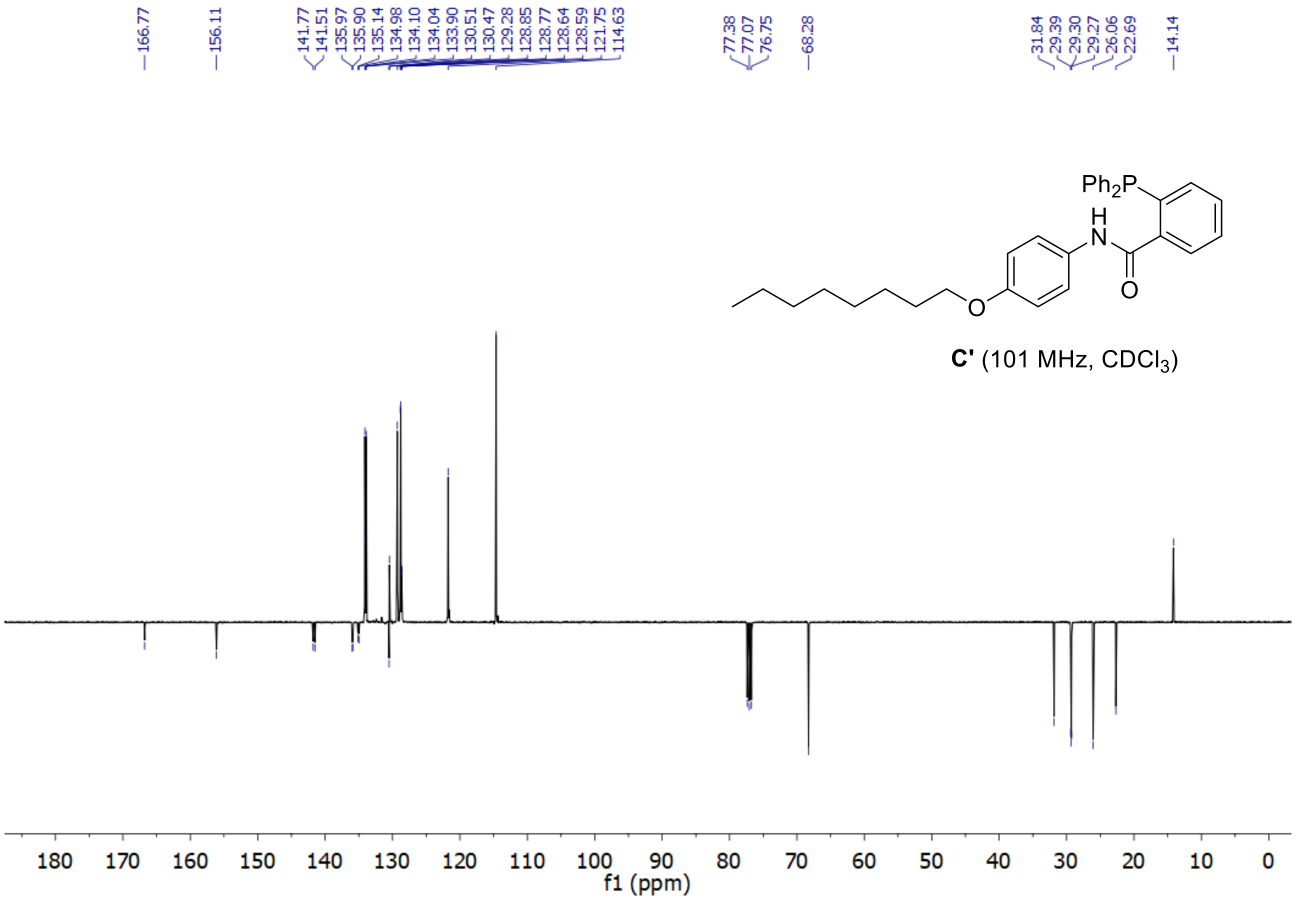

C' (101 MHz, $CDCl_3$)

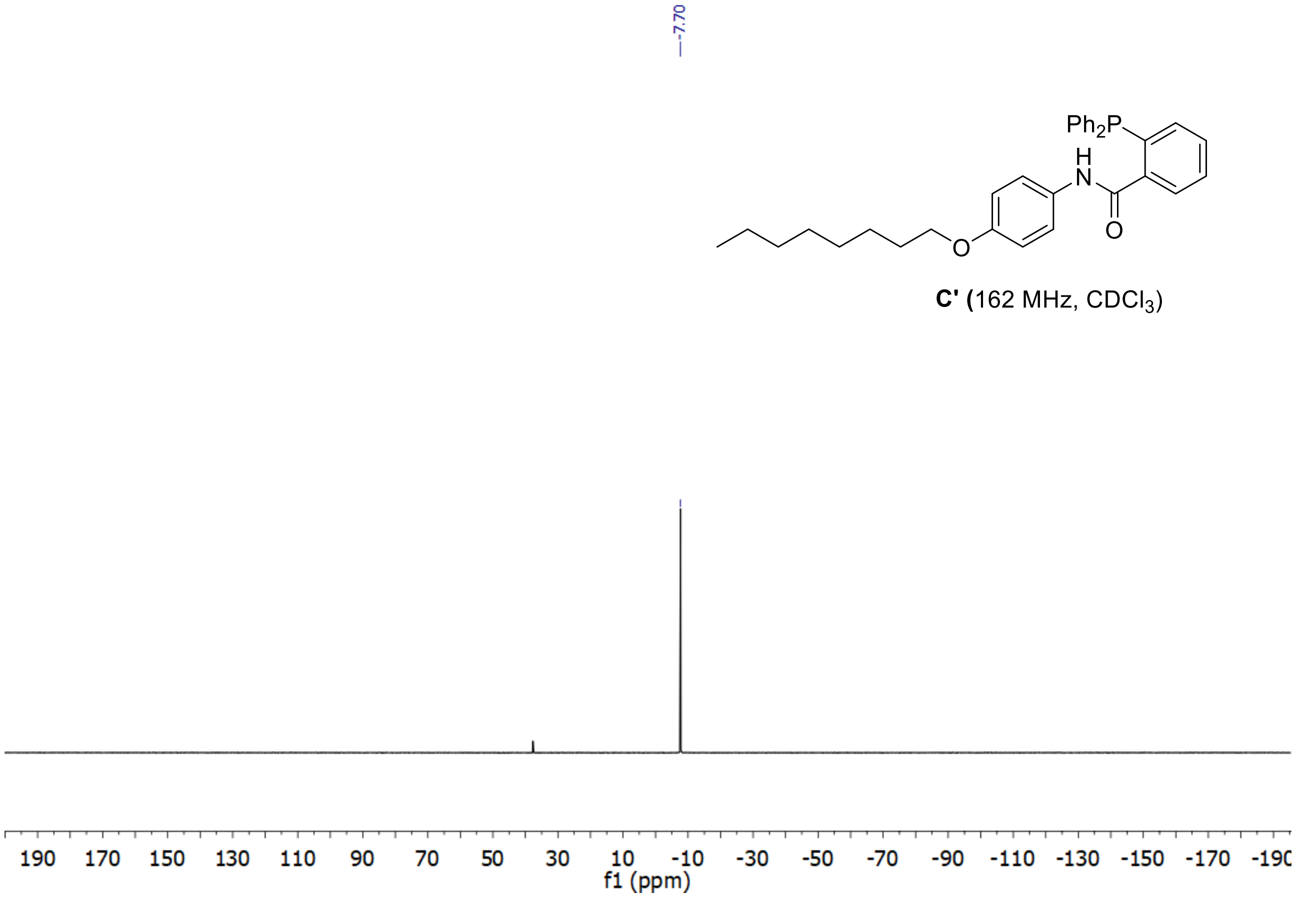

C' (162 MHz, $CDCl_3$)

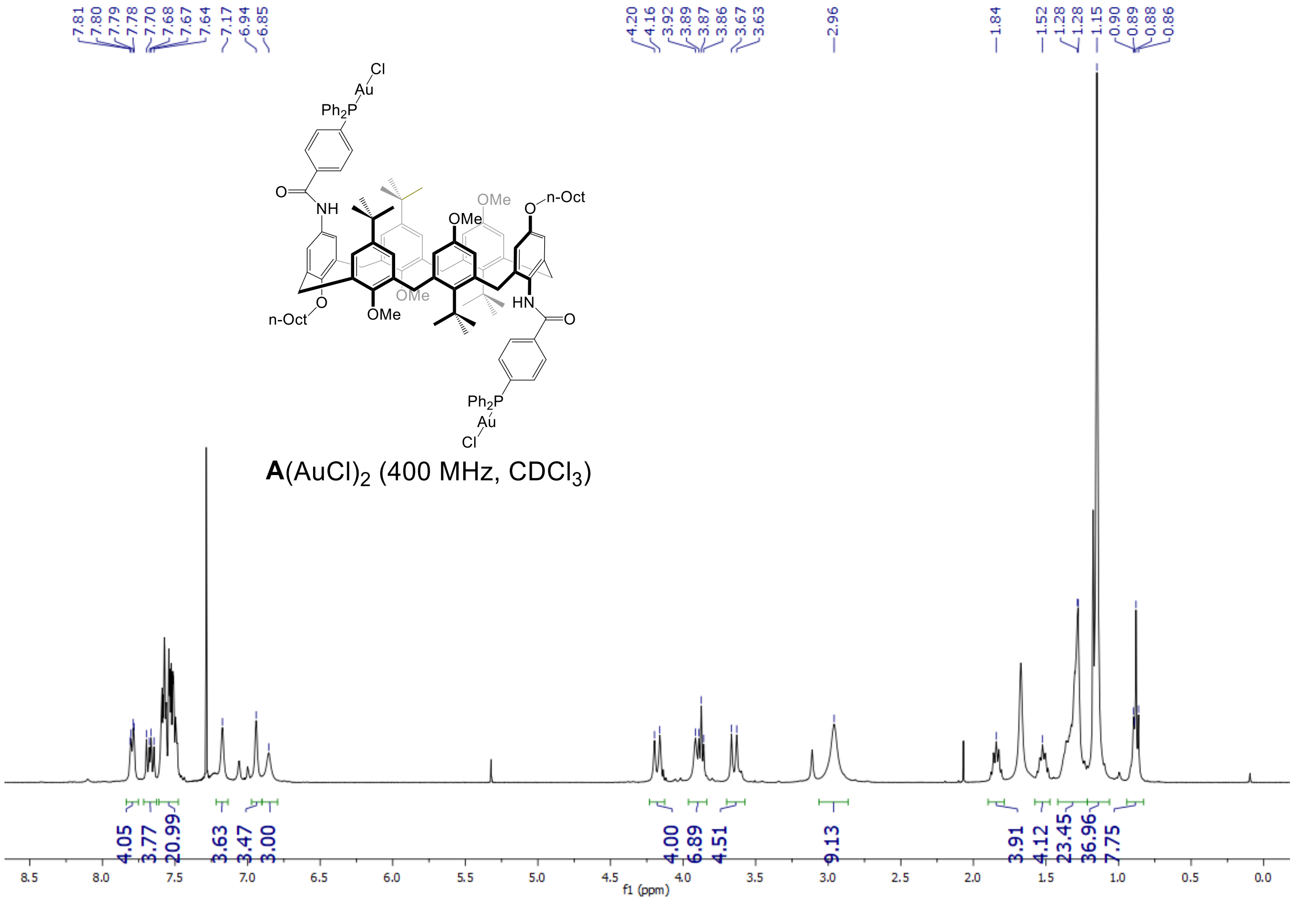

A$(AuCl)_2$ (400 MHz, $CDCl_3$)

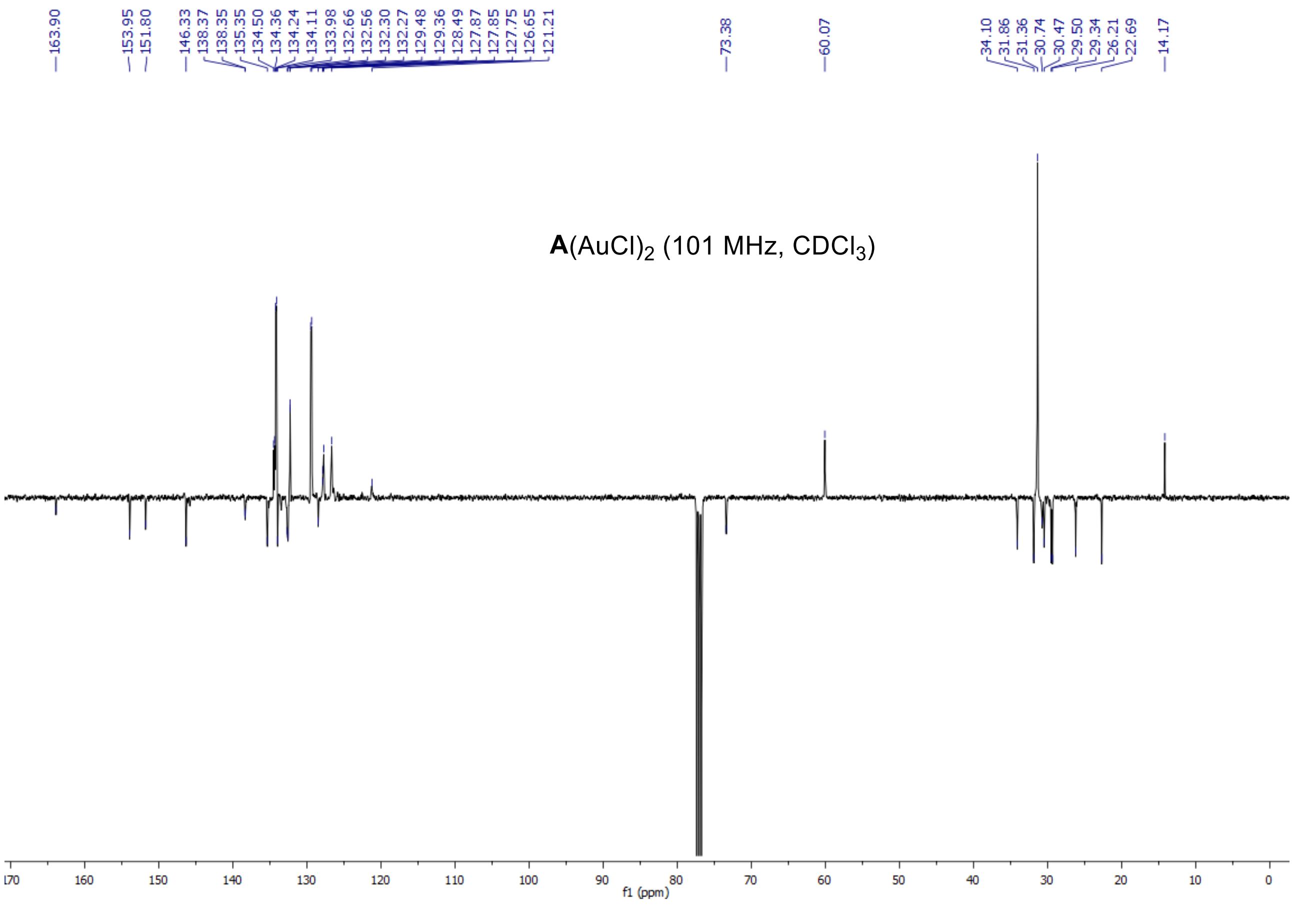

A$(AuCl)_2$ (101 MHz, $CDCl_3$)

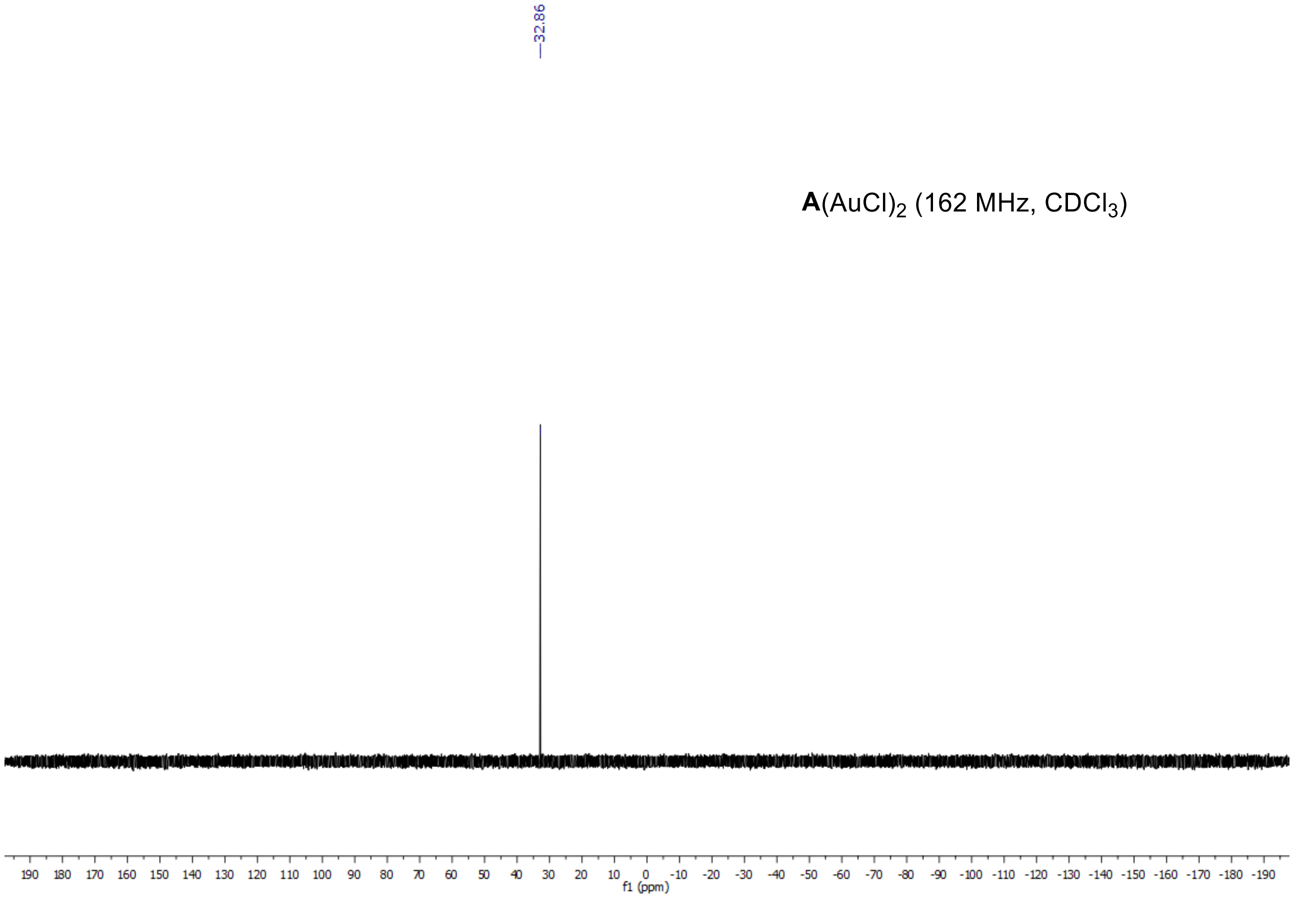

A$(AuCl)_2$ (162 MHz, $CDCl_3$)

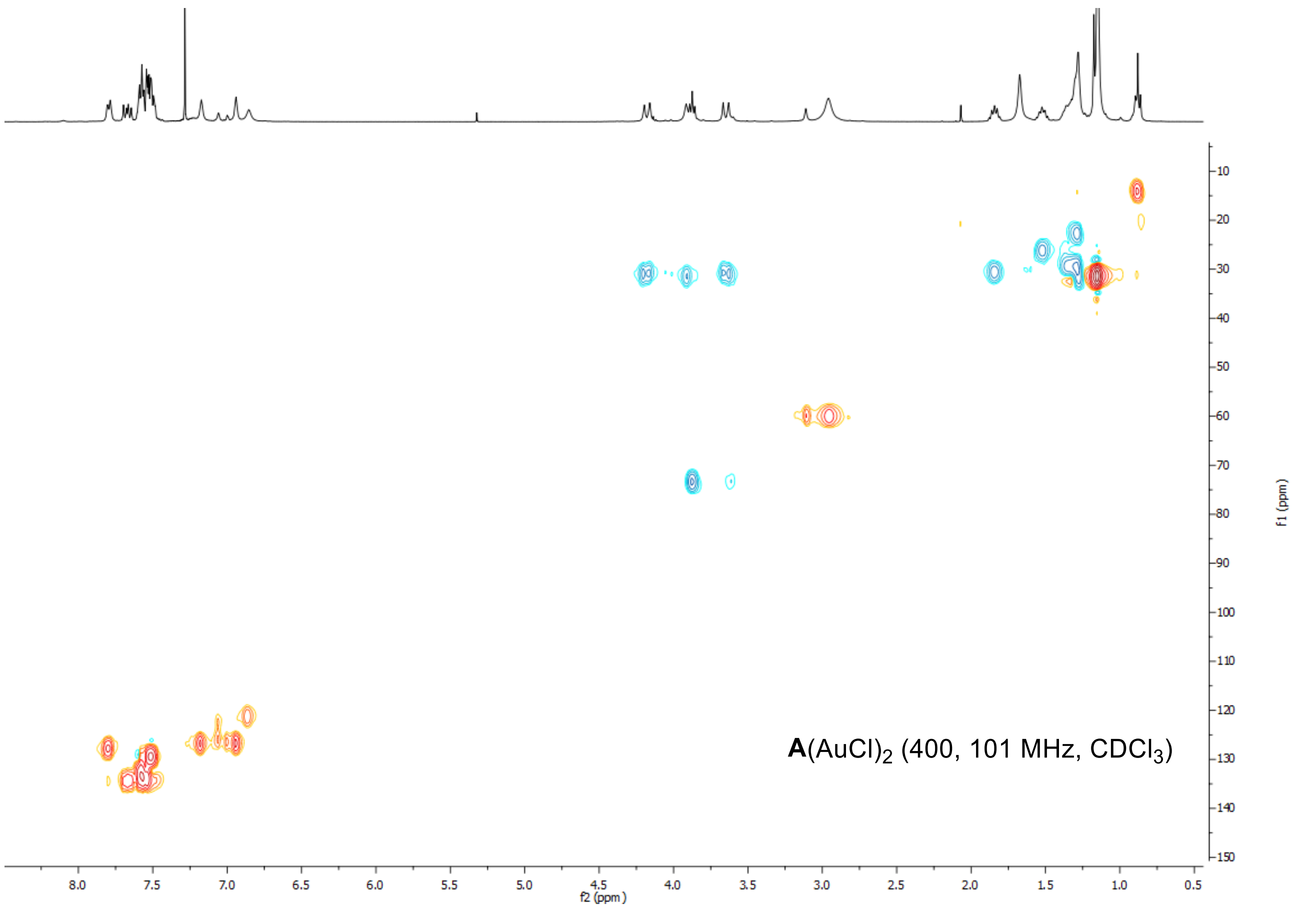
A(AuCl)2 (400, 101 MHz, CDCl3)
f2 (ppm)
f1 (ppm)

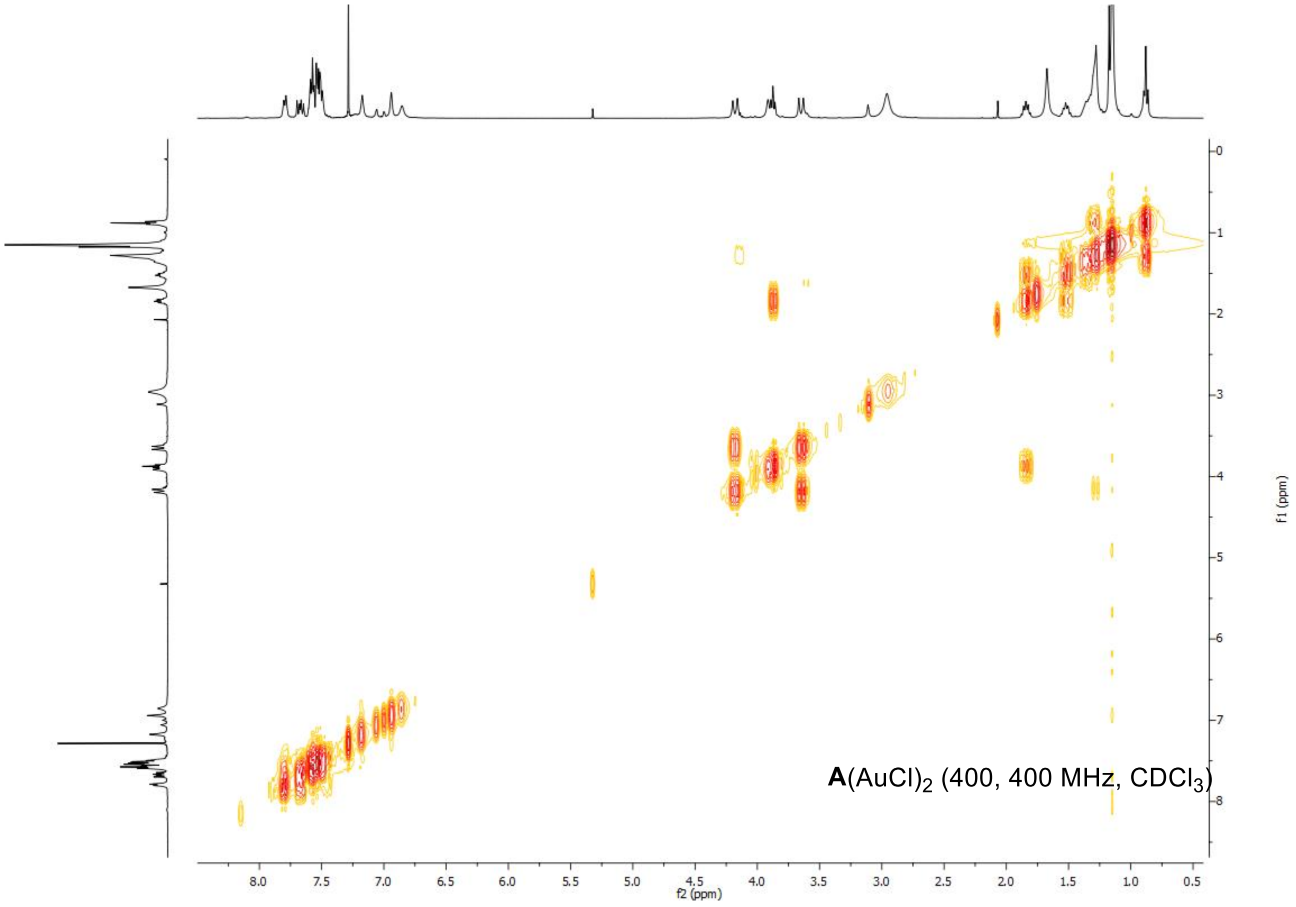
A(AuCl)2 (400, 400 MHz, CDCl3)
f2 (ppm)
f1 (ppm)

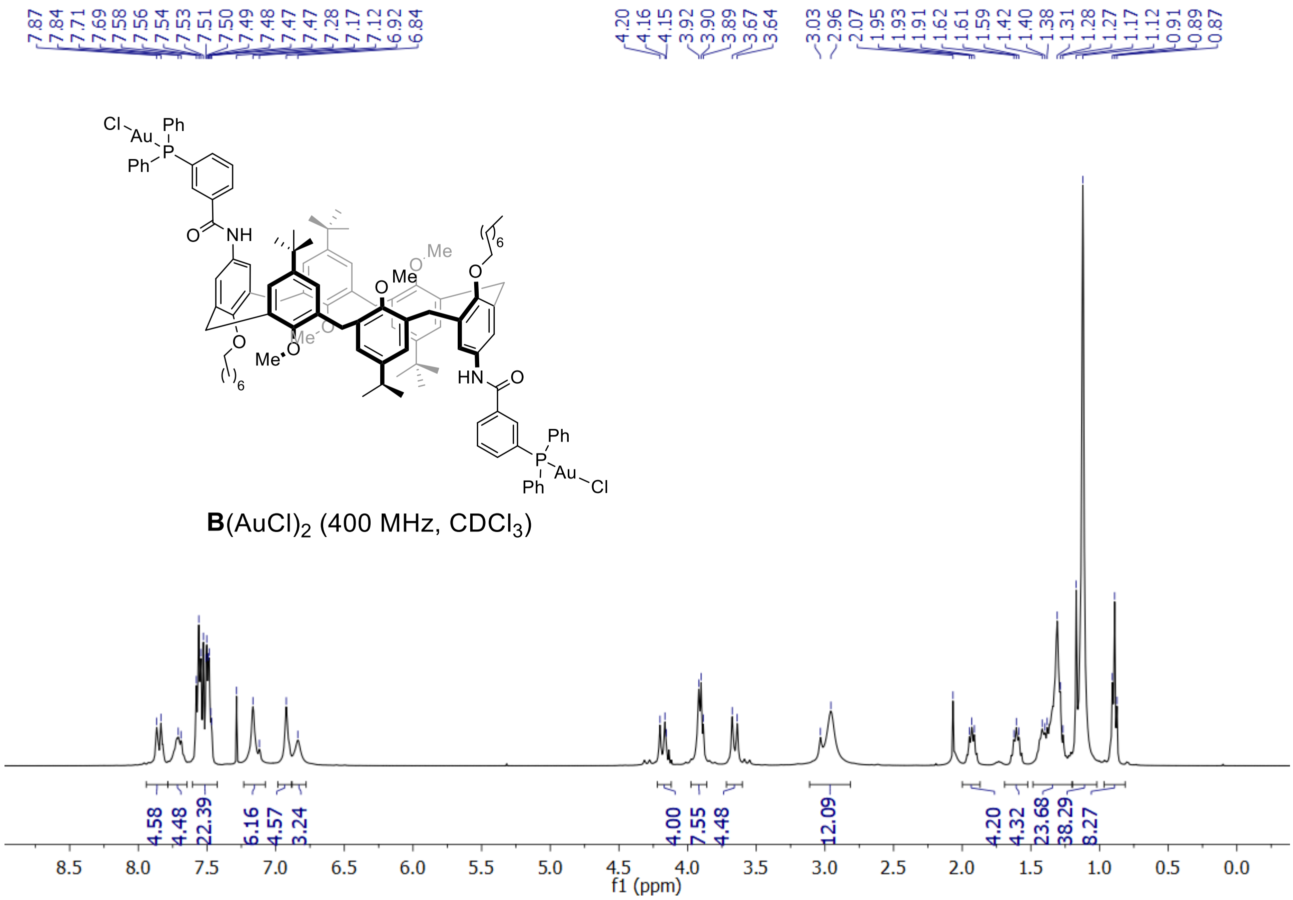

B$(AuCl)_2$ (400 MHz, $CDCl_3$)

B$(AuCl)_2$ (101 MHz, $CDCl_3$)

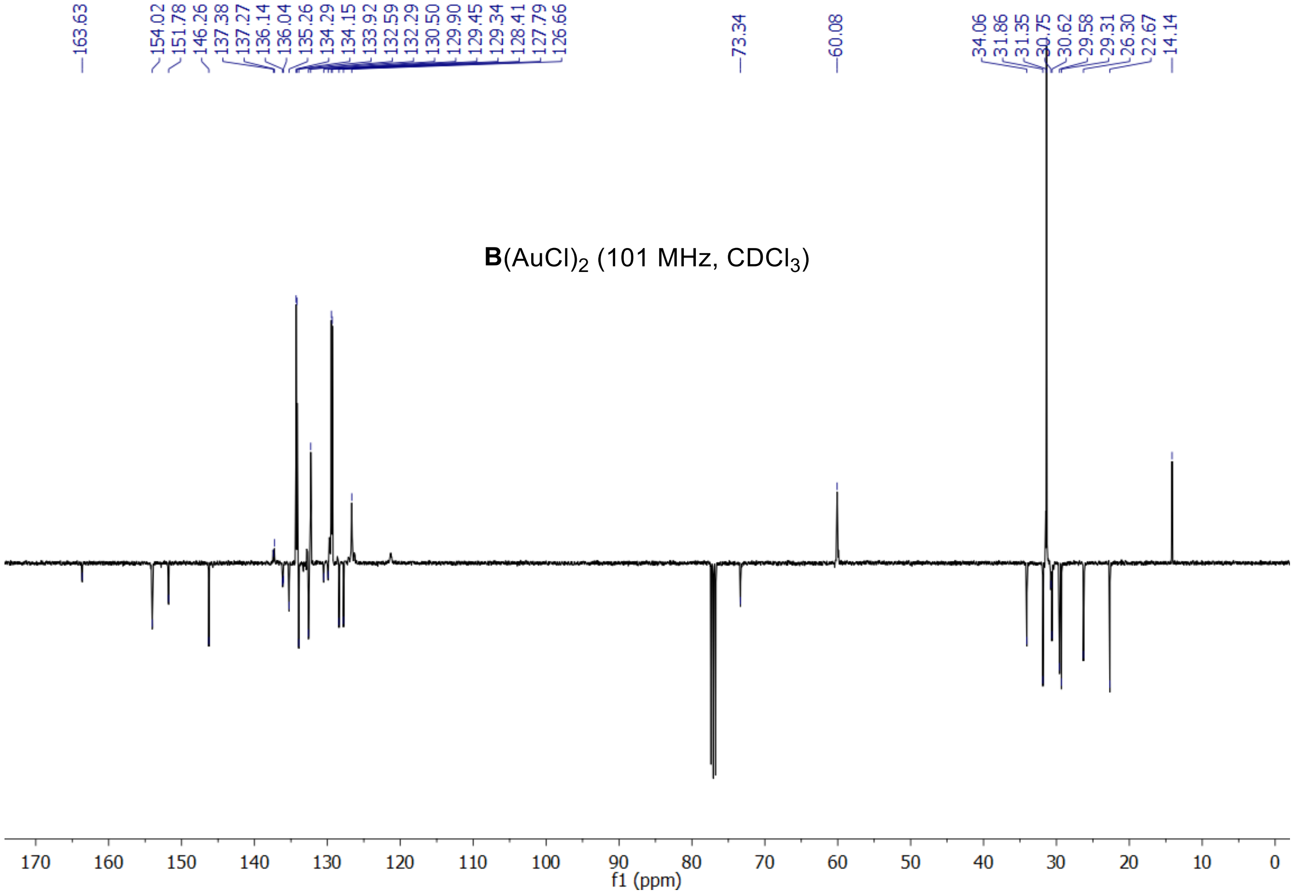

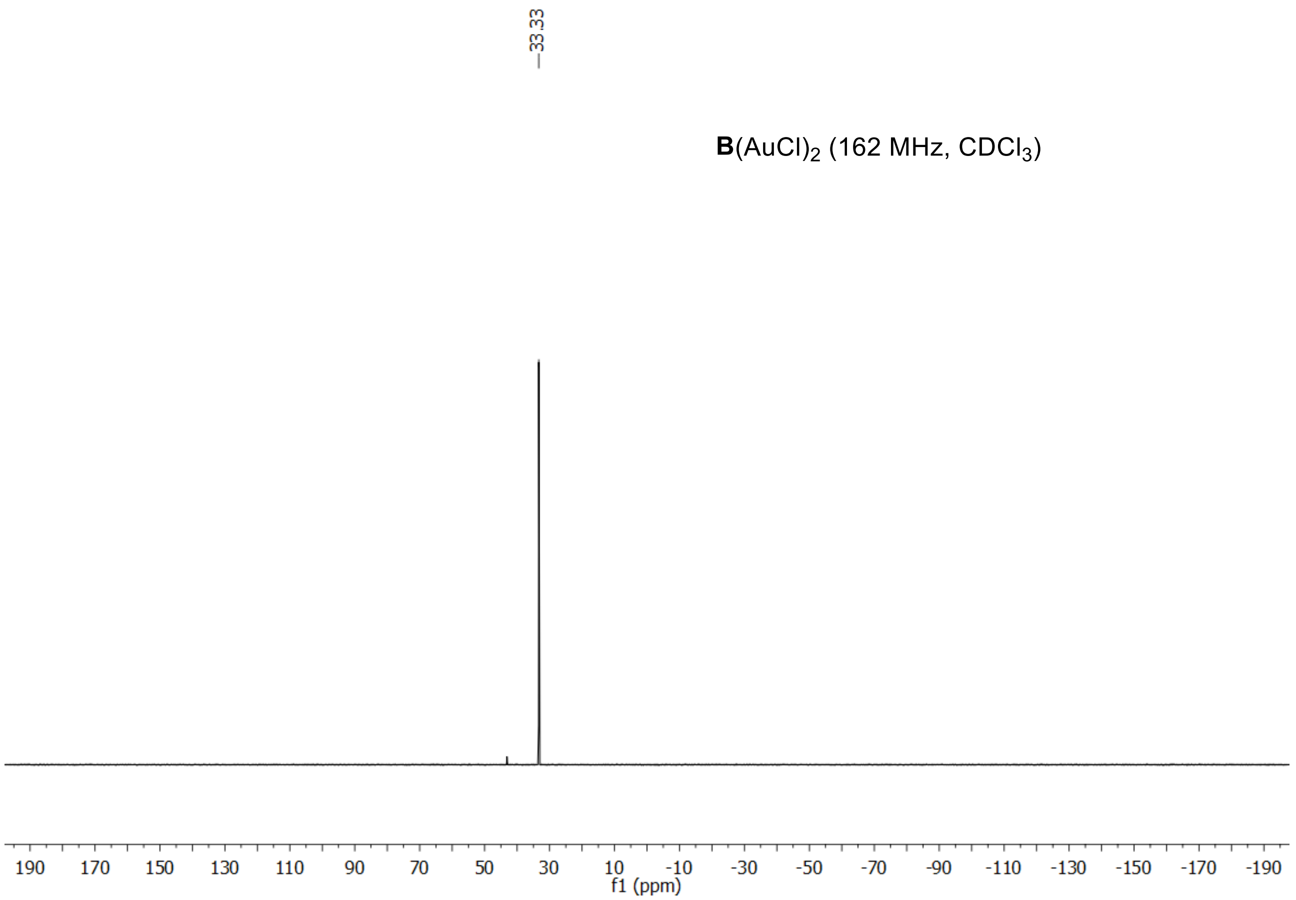
33.33
B(AuCl)2 (162 MHz, CDCl3)
f1 (ppm)

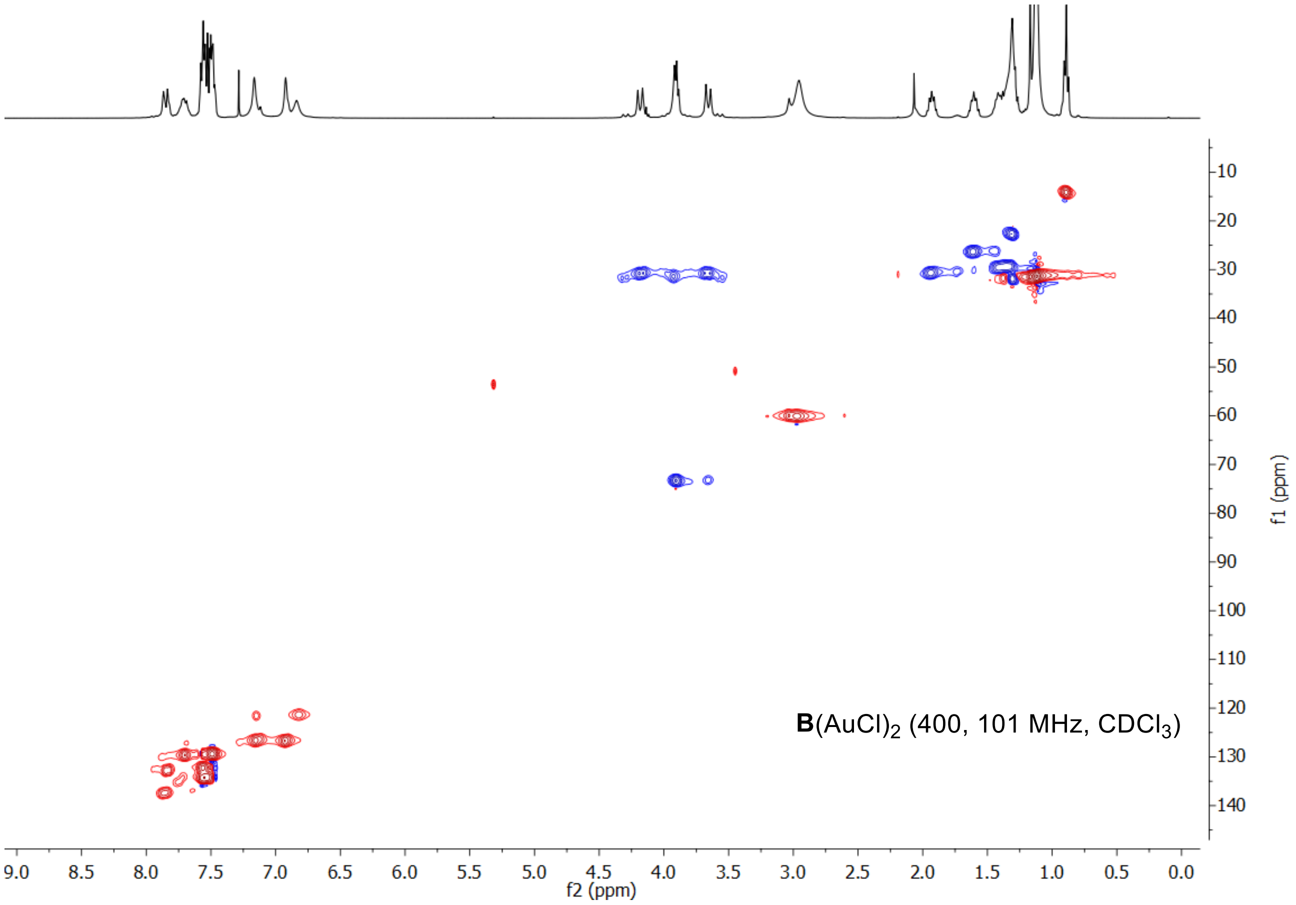
B(AuCl)2 (400, 101 MHz, CDCl3)
f2 (ppm)
f1 (ppm)

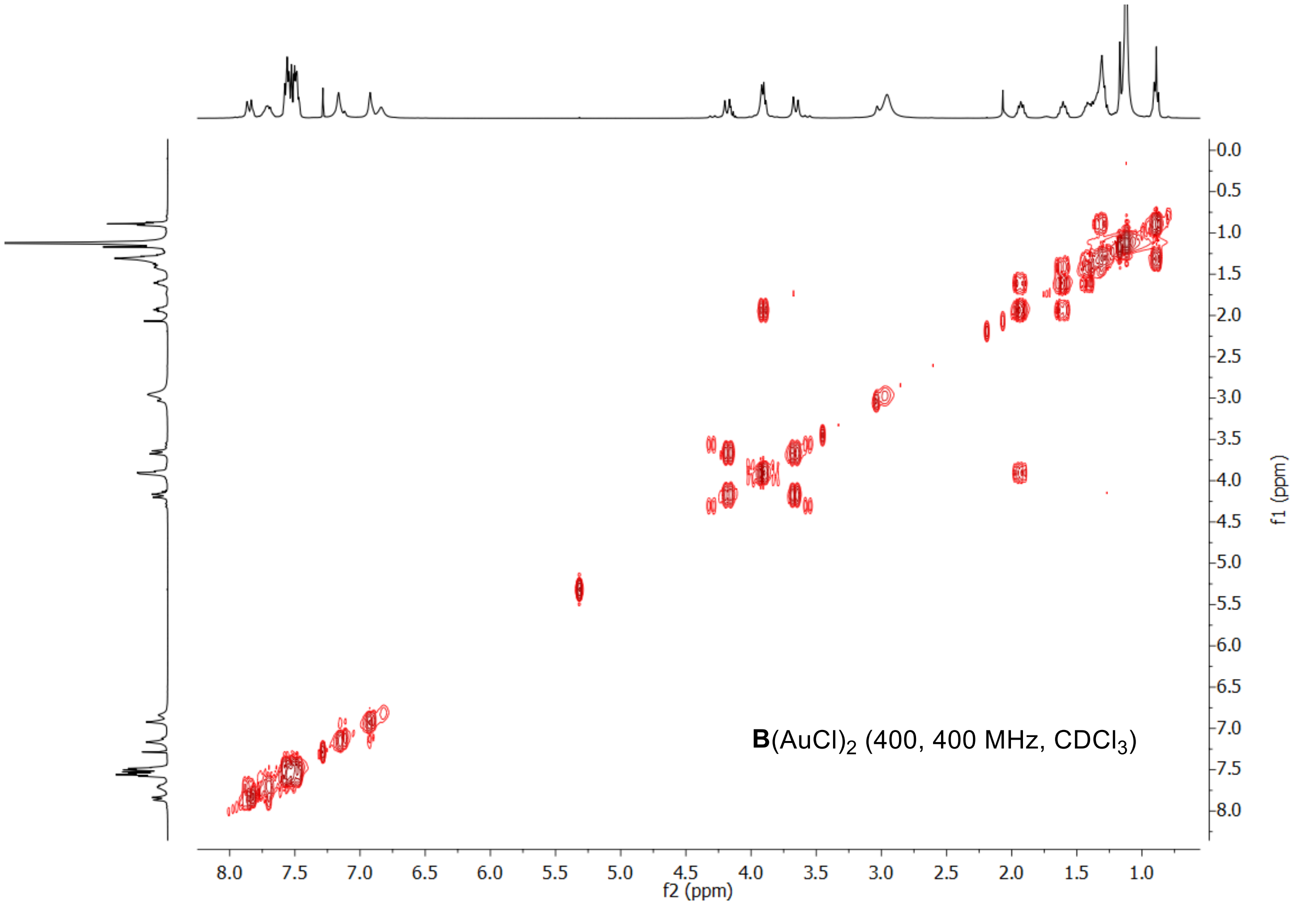
B(AuCl)$_2$ (400, 400 MHz, CDCl$_3$)
f2 (ppm)
f1 (ppm)

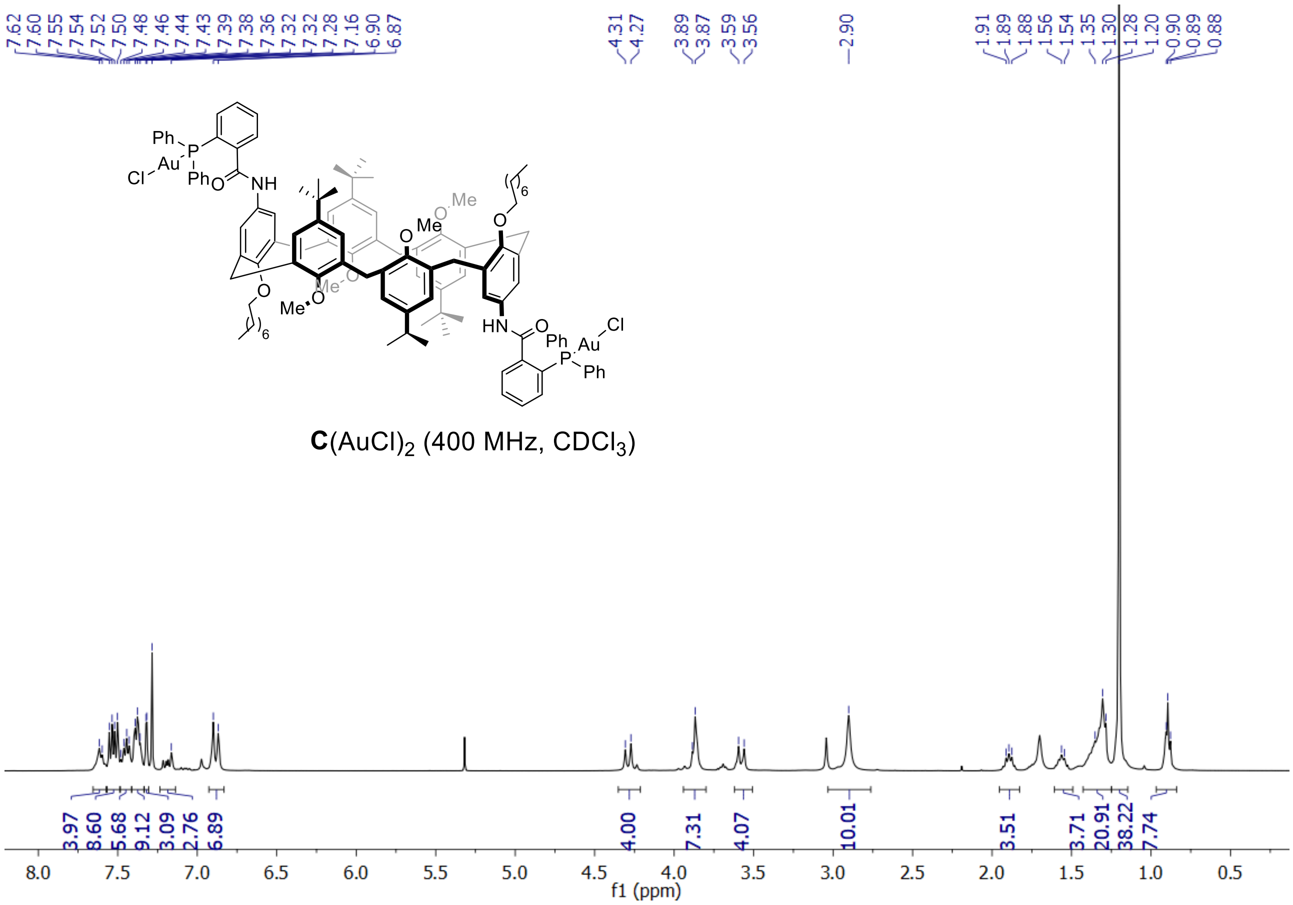

C$(AuCl)_2$ (400 MHz, $CDCl_3$)

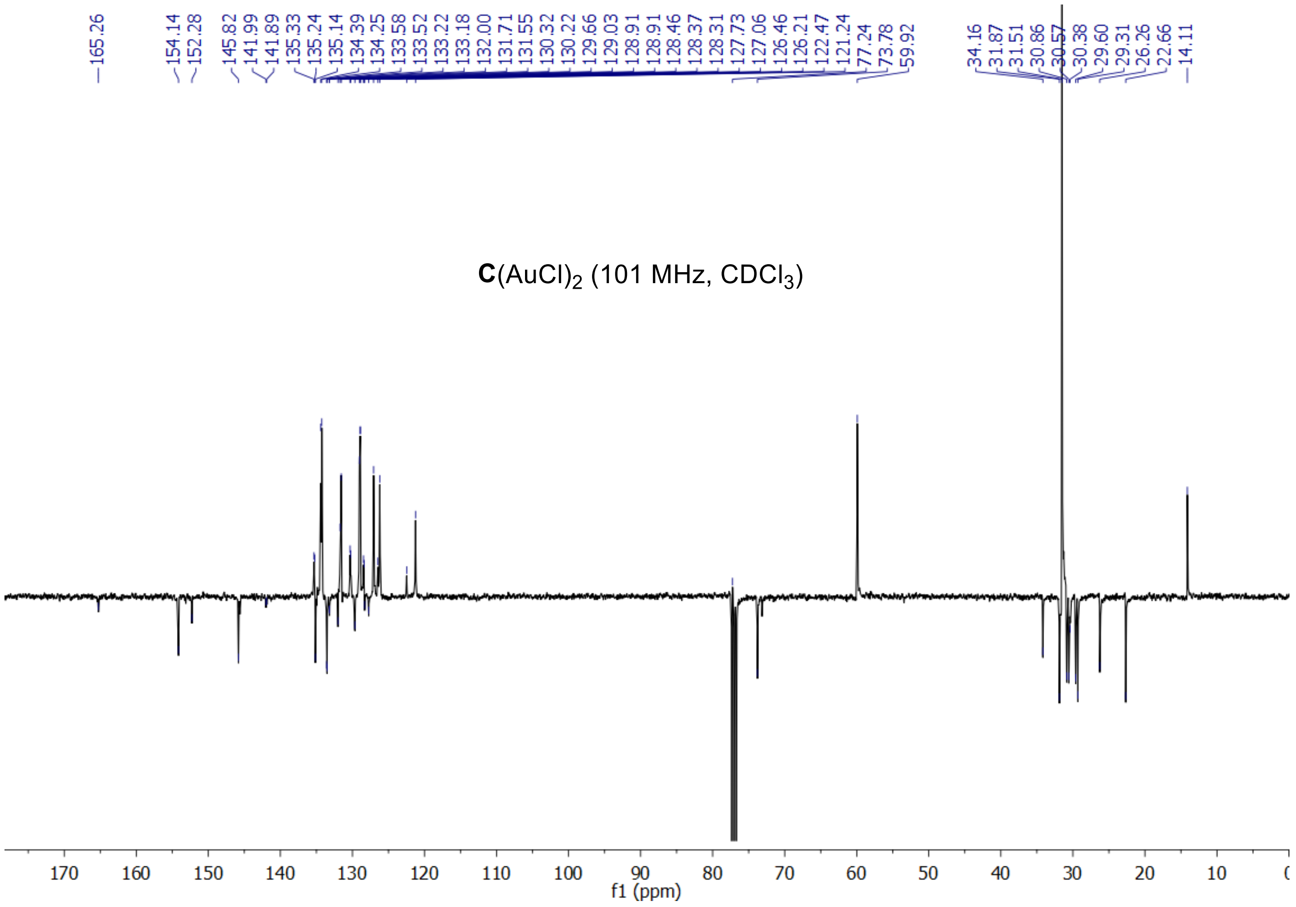

C(AuCl)2 (101 MHz, CDCl3)
165.26
154.14
152.28
145.82
141.99
141.89
135.33
135.24
135.14
134.39
134.25
133.58
133.52
133.22
133.18
132.00
131.71
131.55
130.32
130.22
129.66
129.03
128.91
128.46
128.37
128.31
127.73
127.06
126.46
126.21
122.47
121.24
77.24
73.78
59.92
34.16
31.87
31.51
30.86
30.57
30.38
29.60
29.31
26.26
22.66
14.11
f1 (ppm)

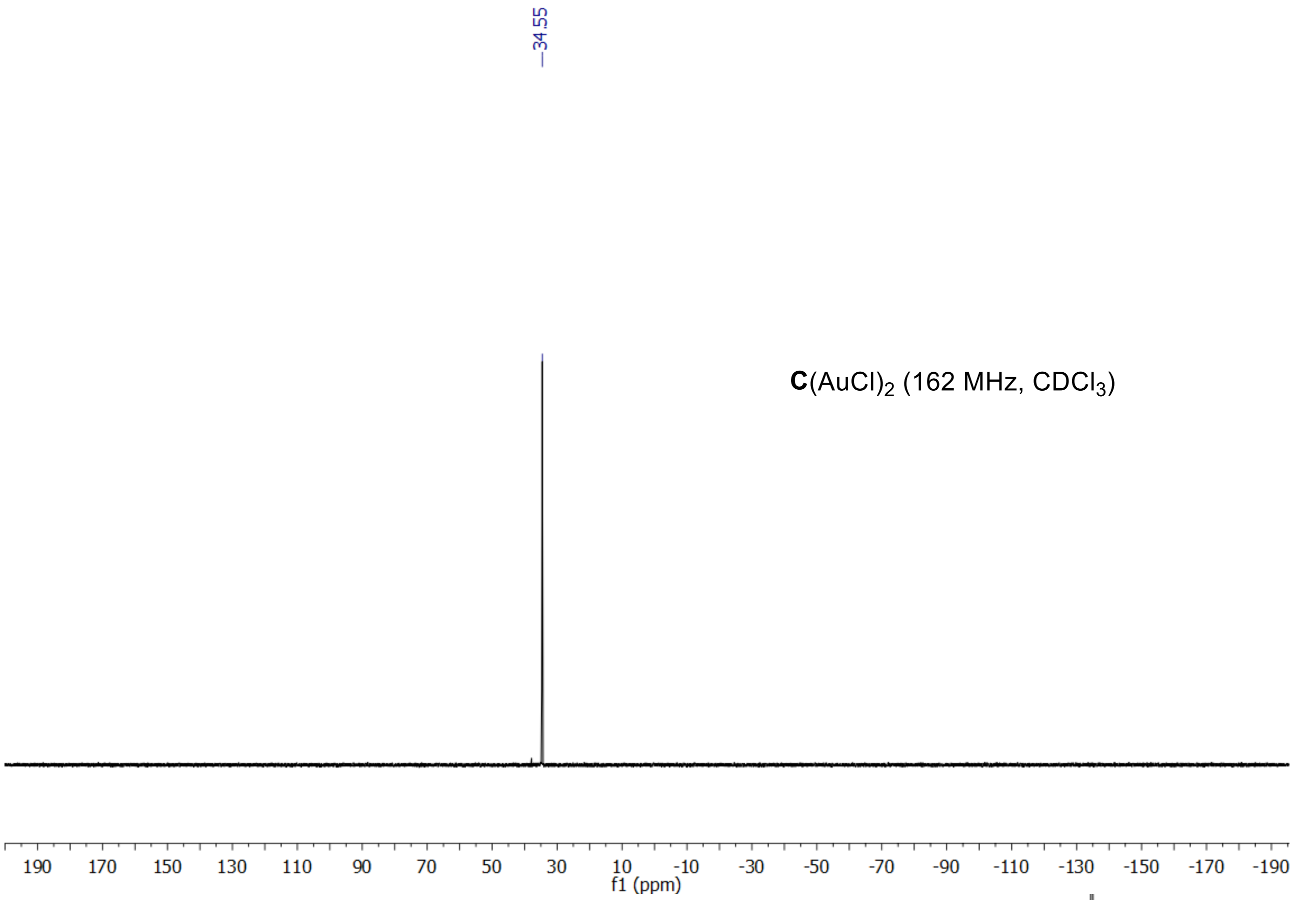
34.55
C(AuCl)2 (162 MHz, CDCl3)
190 170 150 130 110 90 70 50 30 10 -10 -30 -50 -70 -90 -110 -130 -150 -170 -190
f1 (ppm)

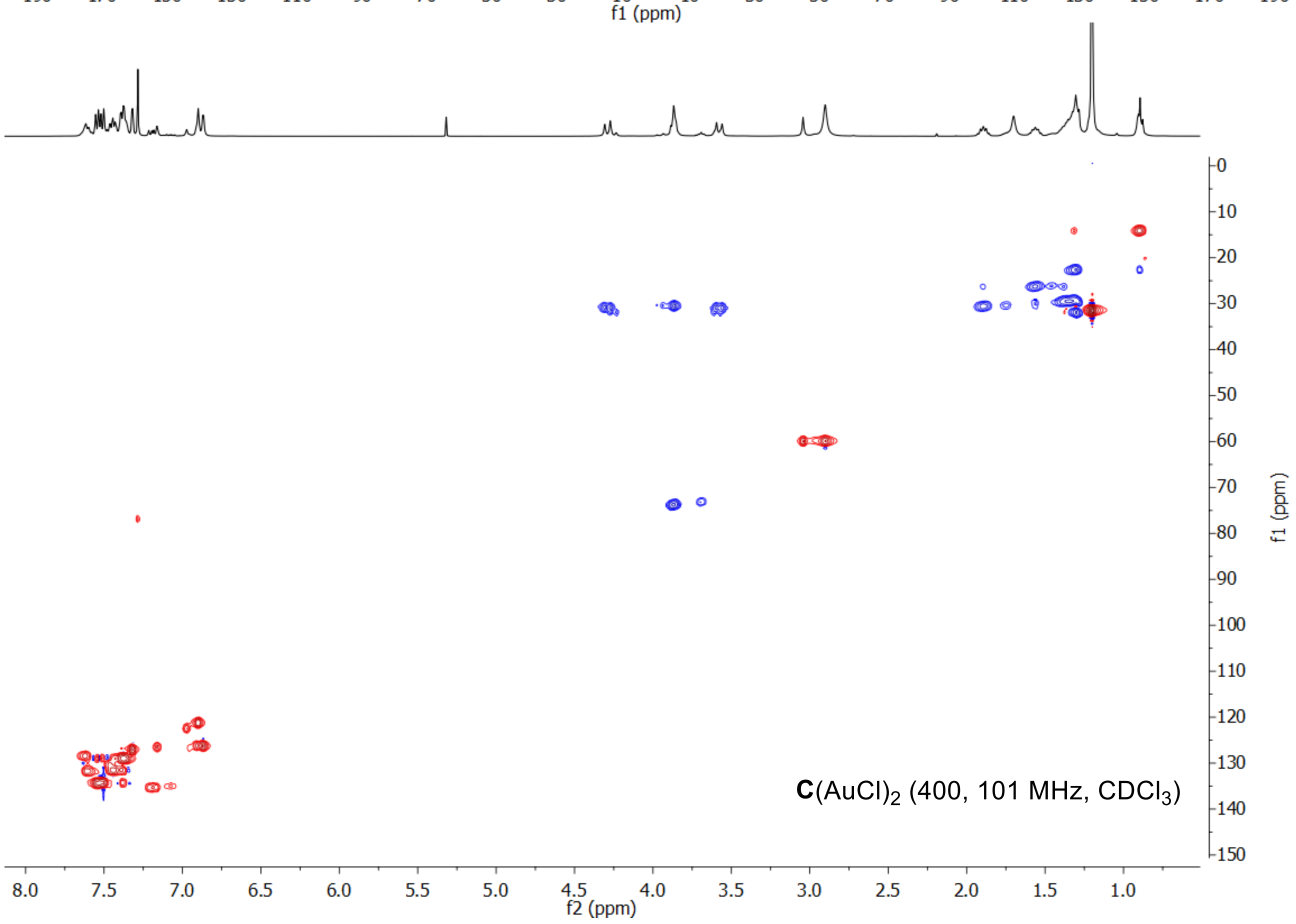
C(AuCl)2 (400, 101 MHz, CDCl3)
8.0 7.5 7.0 6.5 6.0 5.5 5.0 4.5 4.0 3.5 3.0 2.5 2.0 1.5 1.0
f2 (ppm)
0 10 20 30 40 50 60 70 80 90 100 110 120 130 140 150
f1 (ppm)

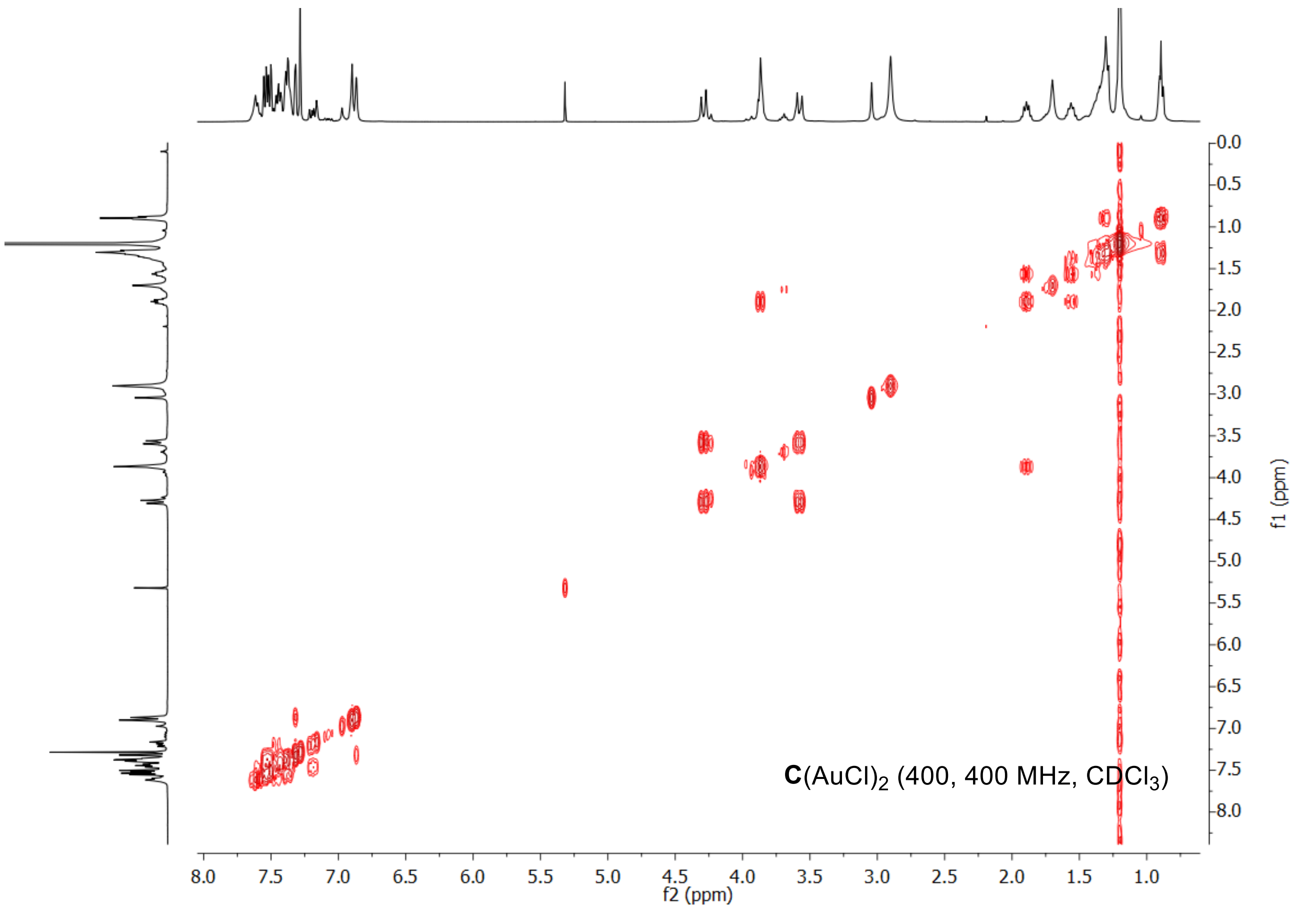

C(AuCl)2 (400, 400 MHz, CDCl3)
f2 (ppm)
f1 (ppm)

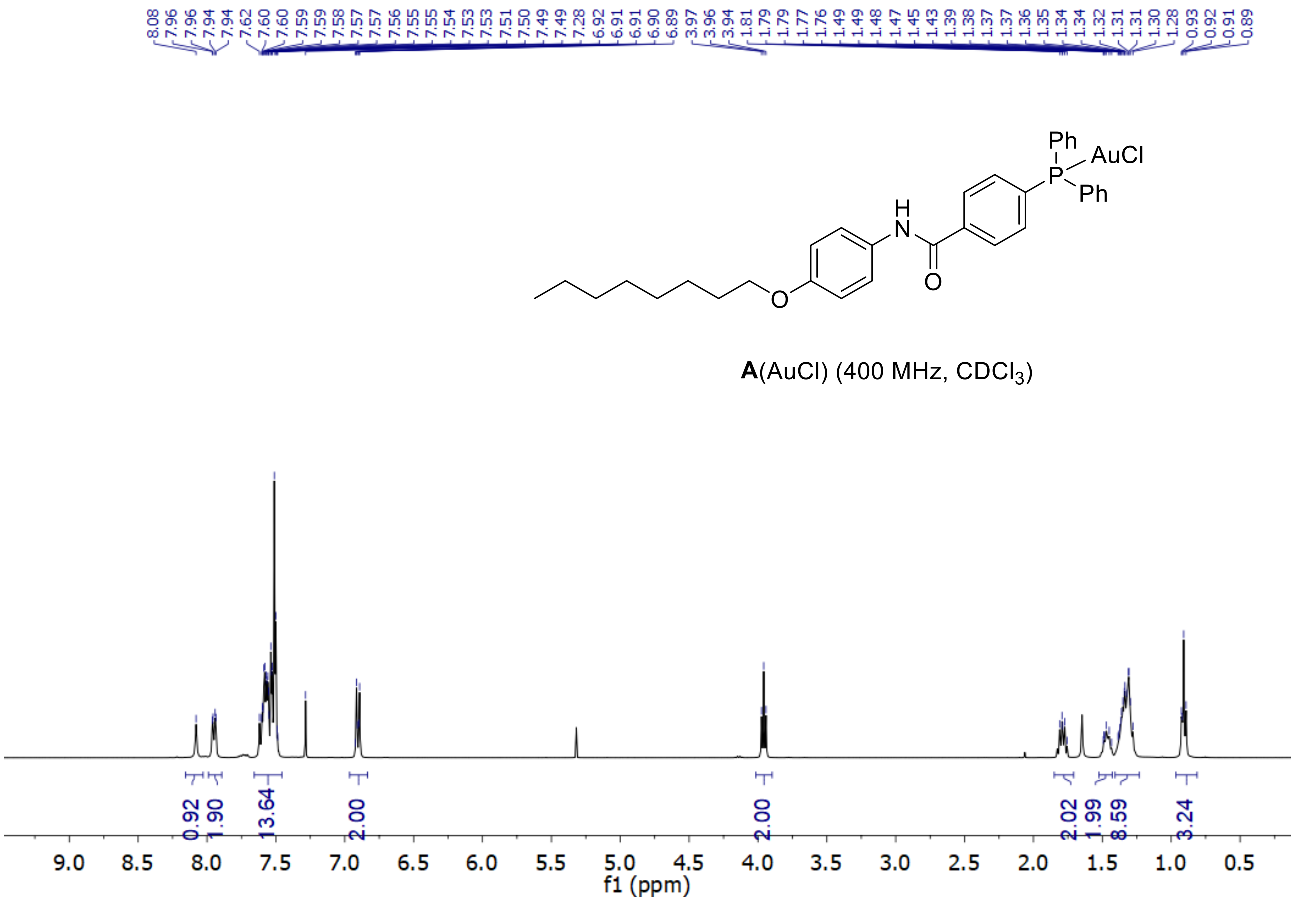
Ph
AuCl
P
Ph
H
N
O
O
A(AuCl) (400 MHz, CDCl3)
0.92
1.90
13.64
2.00
2.00
2.02
1.99
8.59
3.24
f1 (ppm)

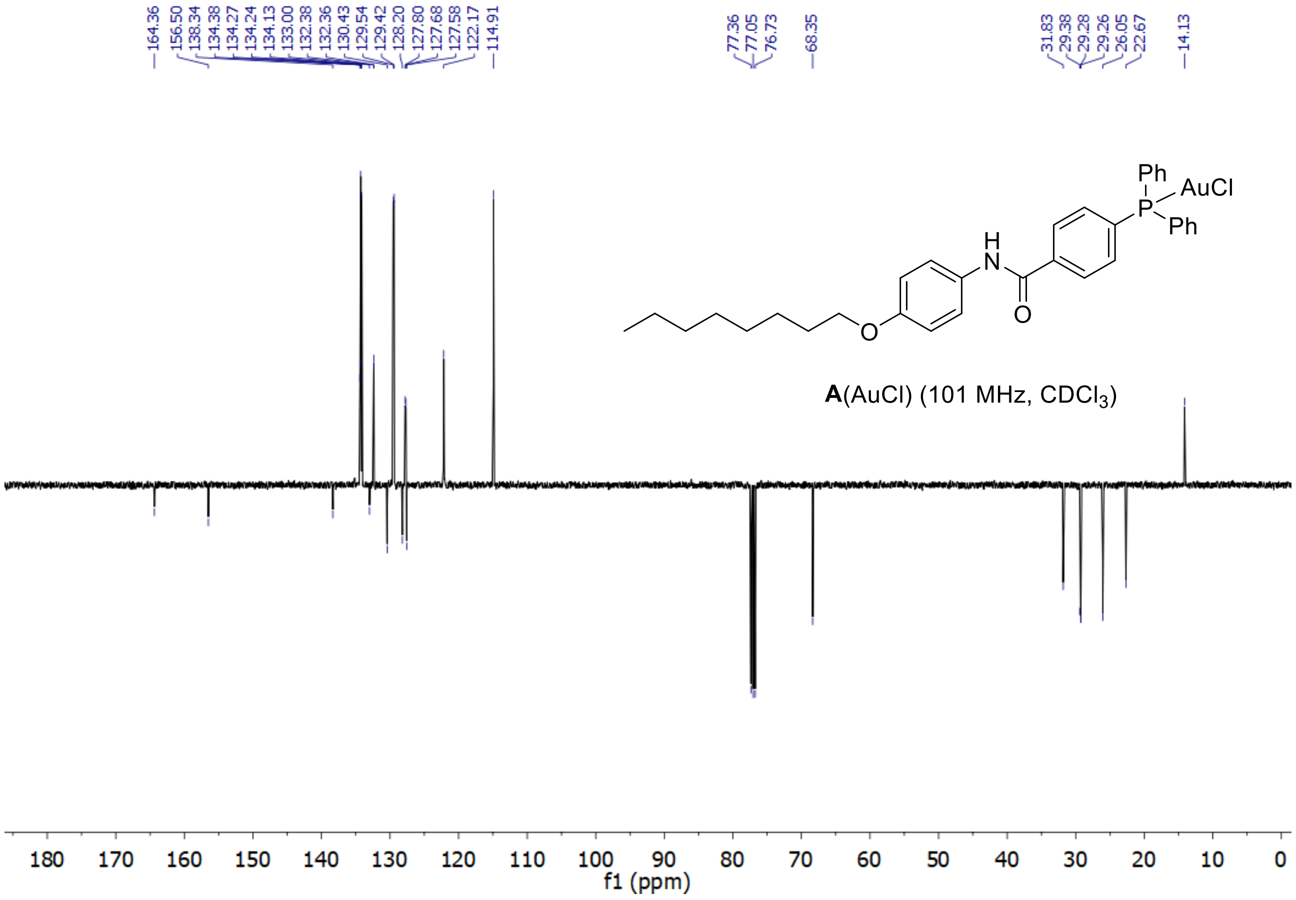
Ph
AuCl
P
Ph
H
N
O
O
A(AuCl) (101 MHz, CDCl3)
f1 (ppm)

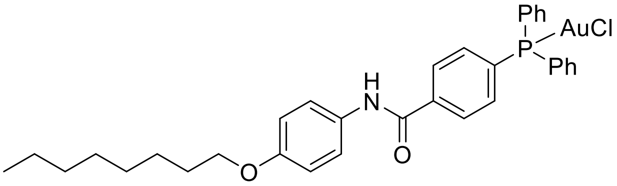

A(AuCl) (162 MHz, $CDCl_3$)

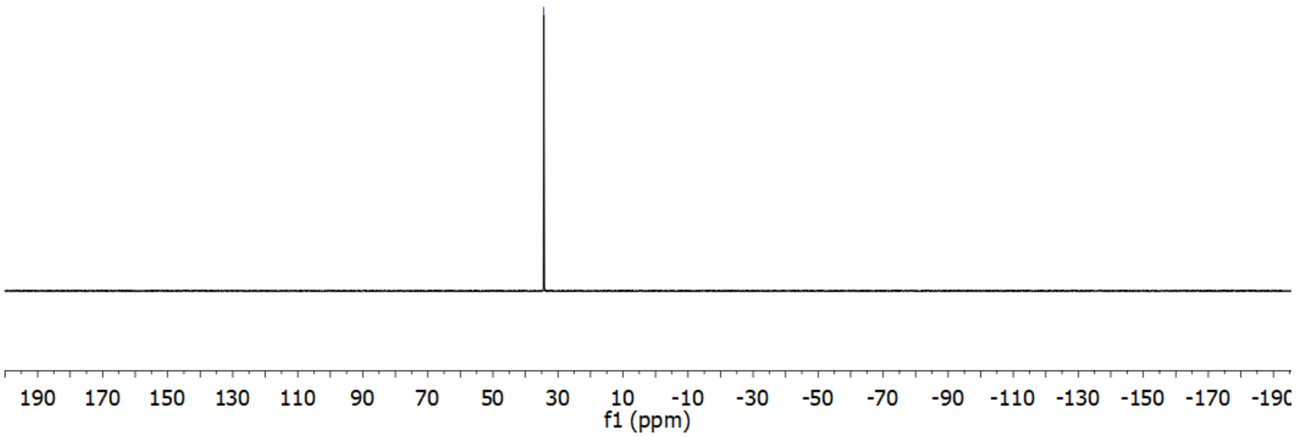

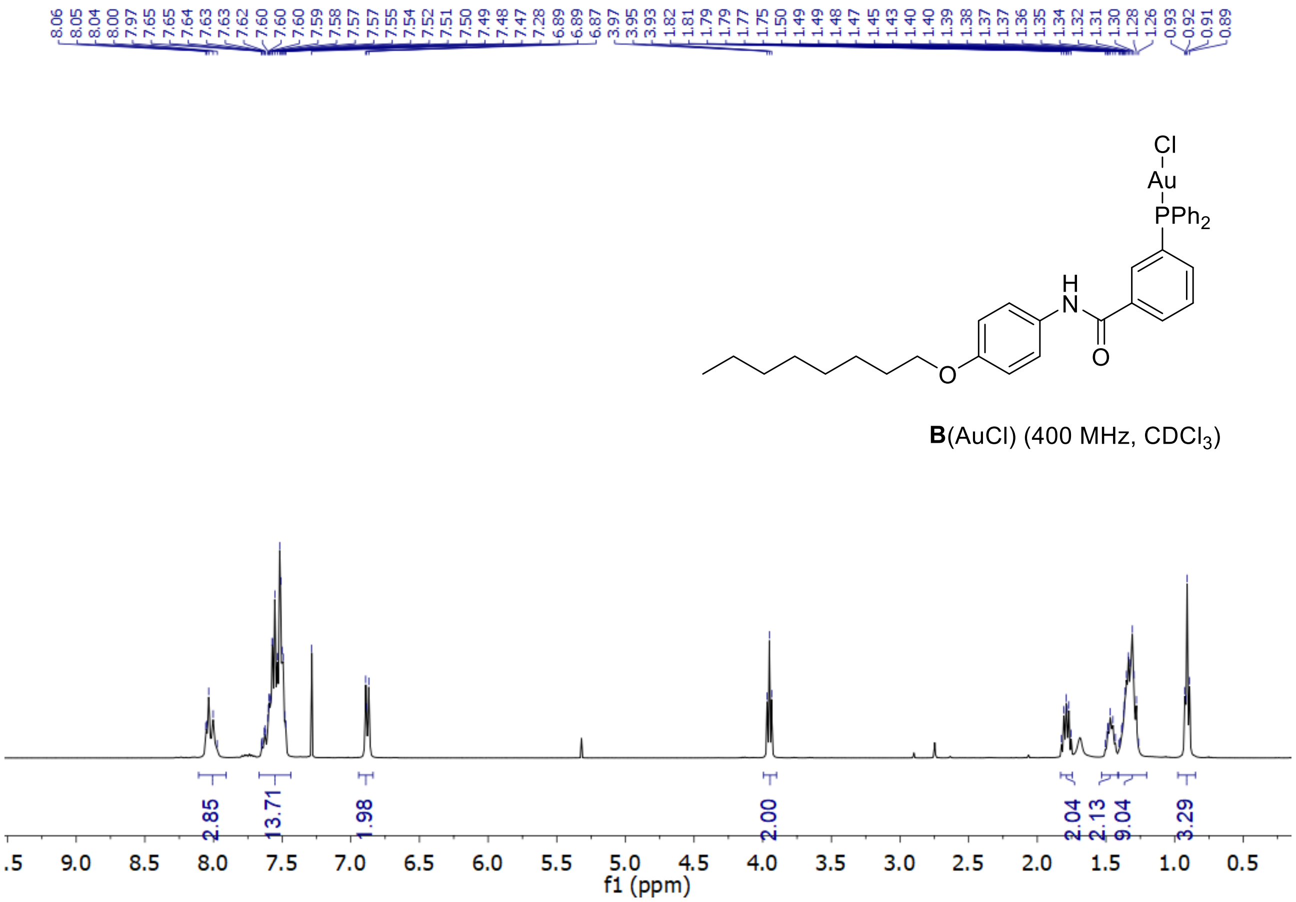
Cl
Au
PPh$_2$
H
N
O
O
B(AuCl) (400 MHz, $CDCl_3$)
f1 (ppm)

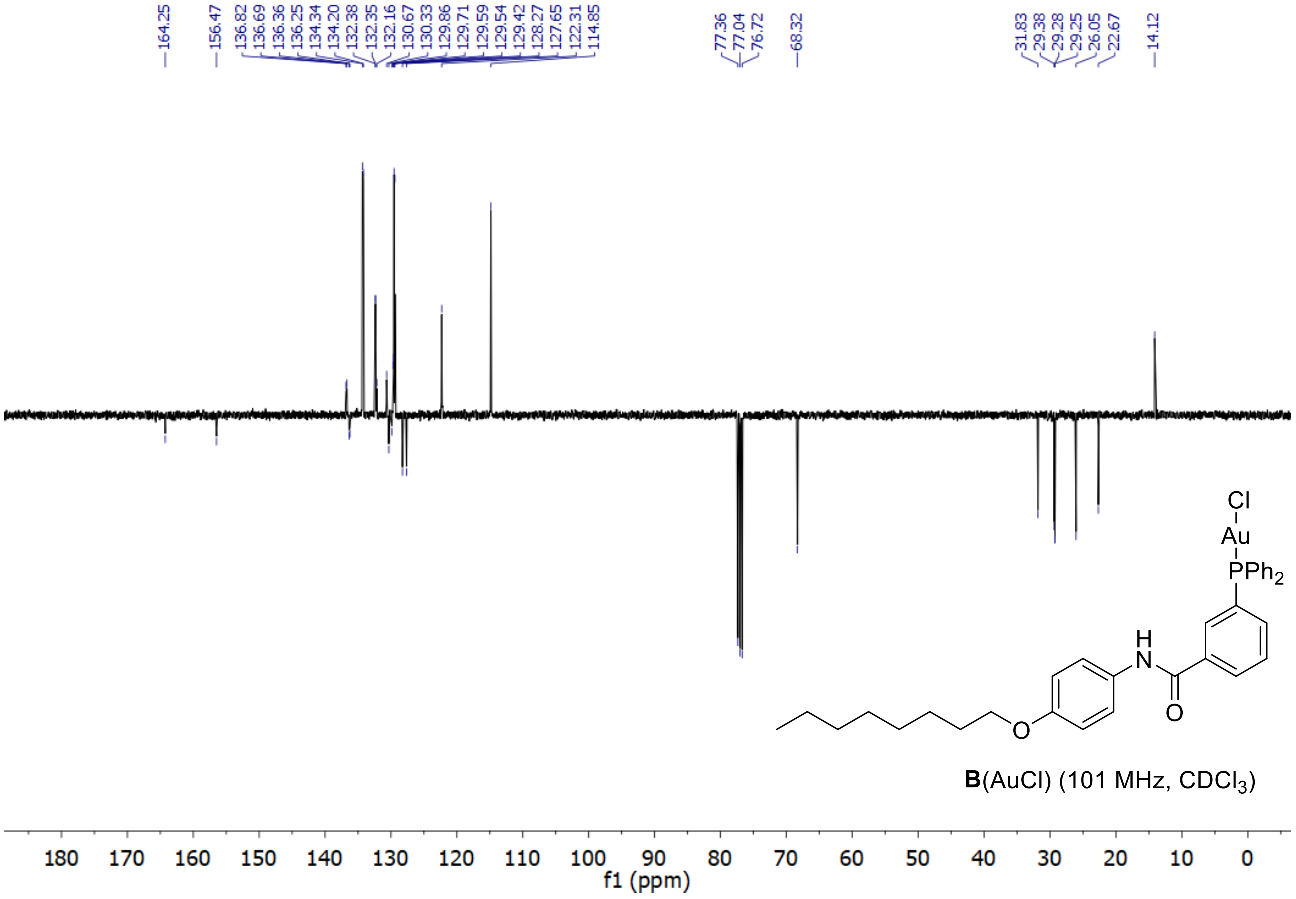
Cl
Au
PPh$_2$
H
N
O
O
B(AuCl) (101 MHz, $CDCl_3$)
f1 (ppm)

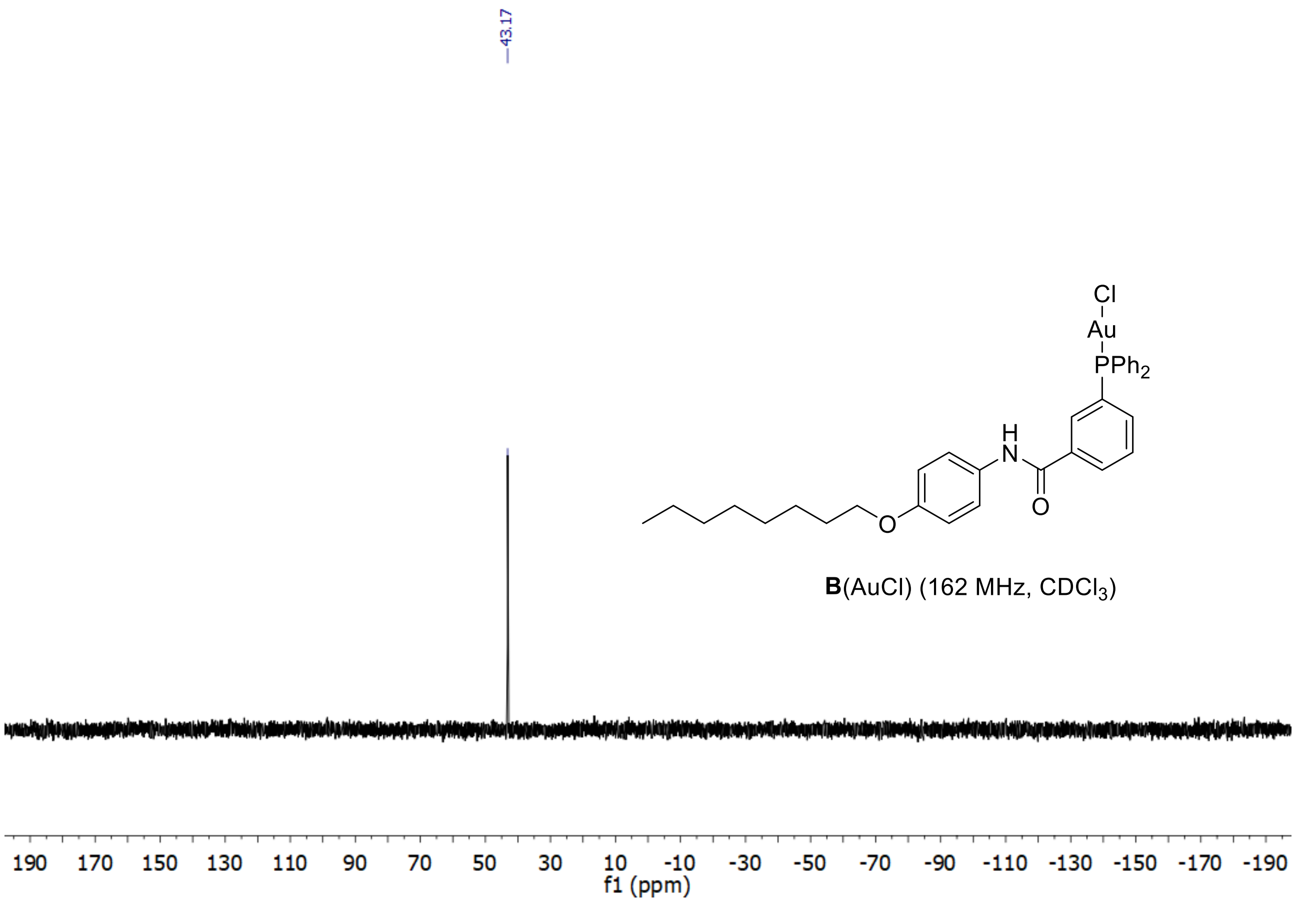

B(AuCl) (162 MHz, $CDCl_3$)

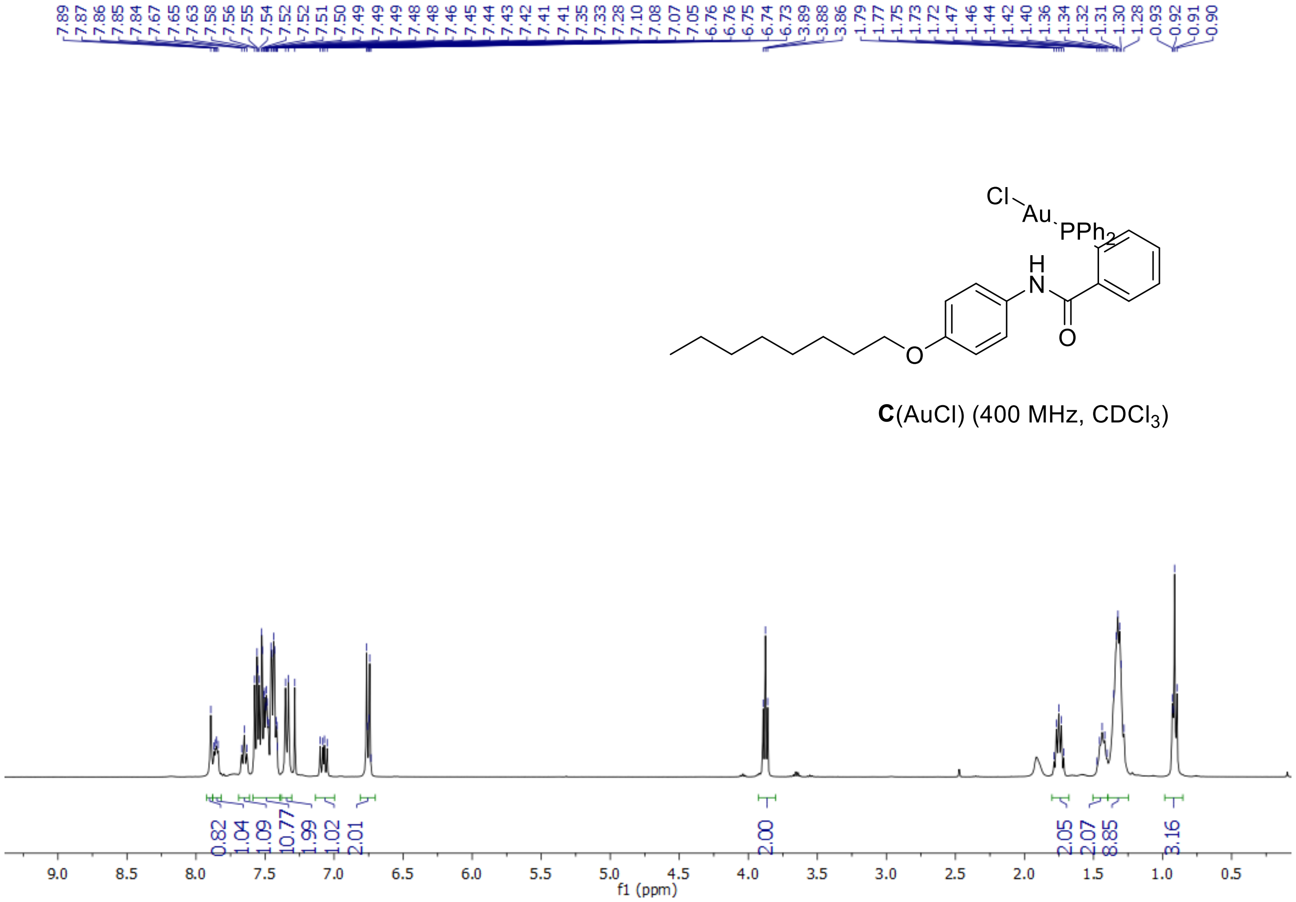

C(AuCl) (400 MHz, $CDCl_3$)

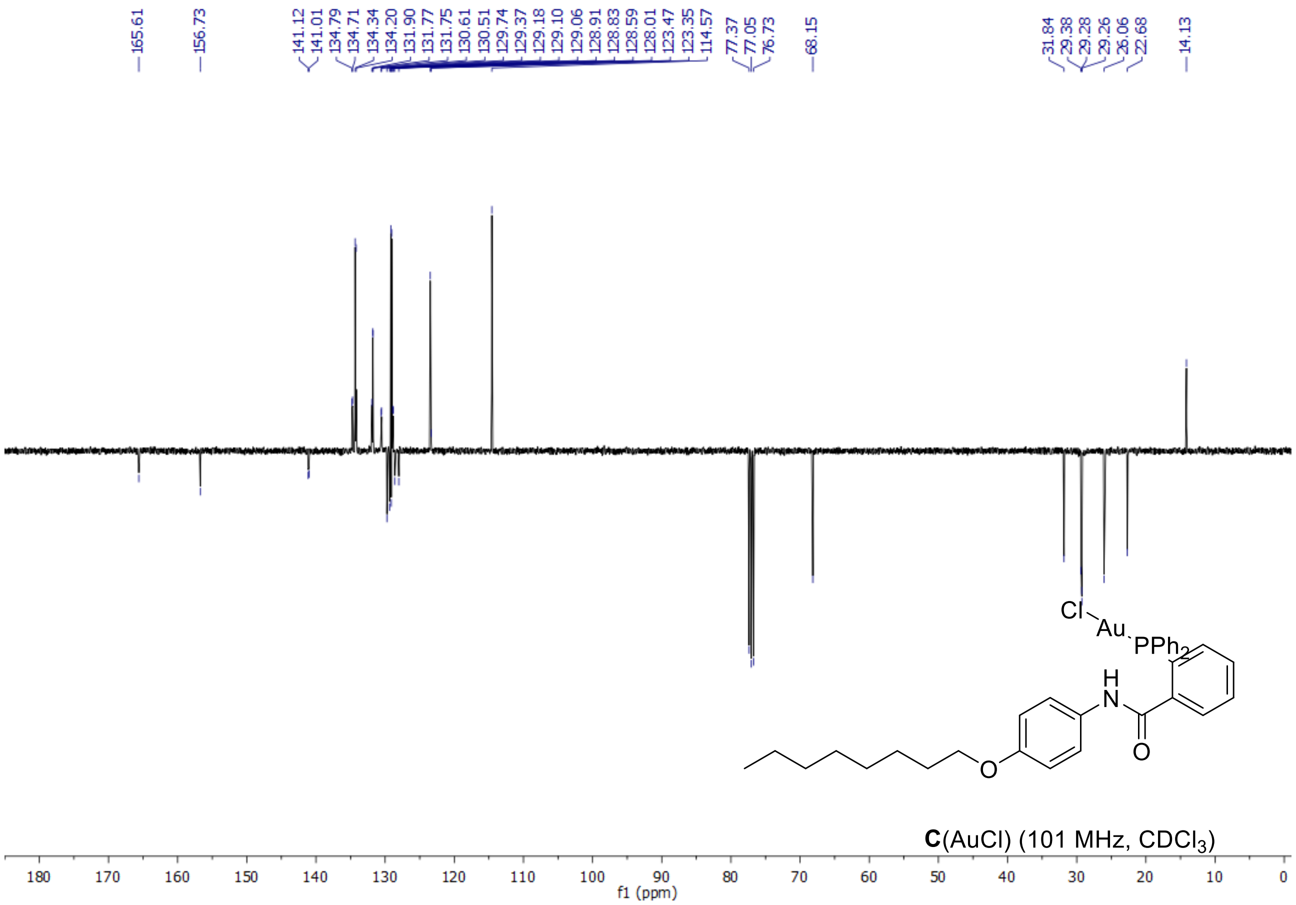
165.61
156.73
141.12
141.01
134.79
134.71
134.34
134.20
131.90
131.77
131.75
130.61
130.51
129.74
129.37
129.18
129.10
129.06
128.91
128.83
128.59
128.01
123.47
123.35
114.57
77.37
77.05
76.73
68.15
31.84
29.38
29.28
29.26
26.06
22.68
14.13
Cl
Au
PPh2
H
N
O
O
C(AuCl) (101 MHz, CDCl3)
f1 (ppm)

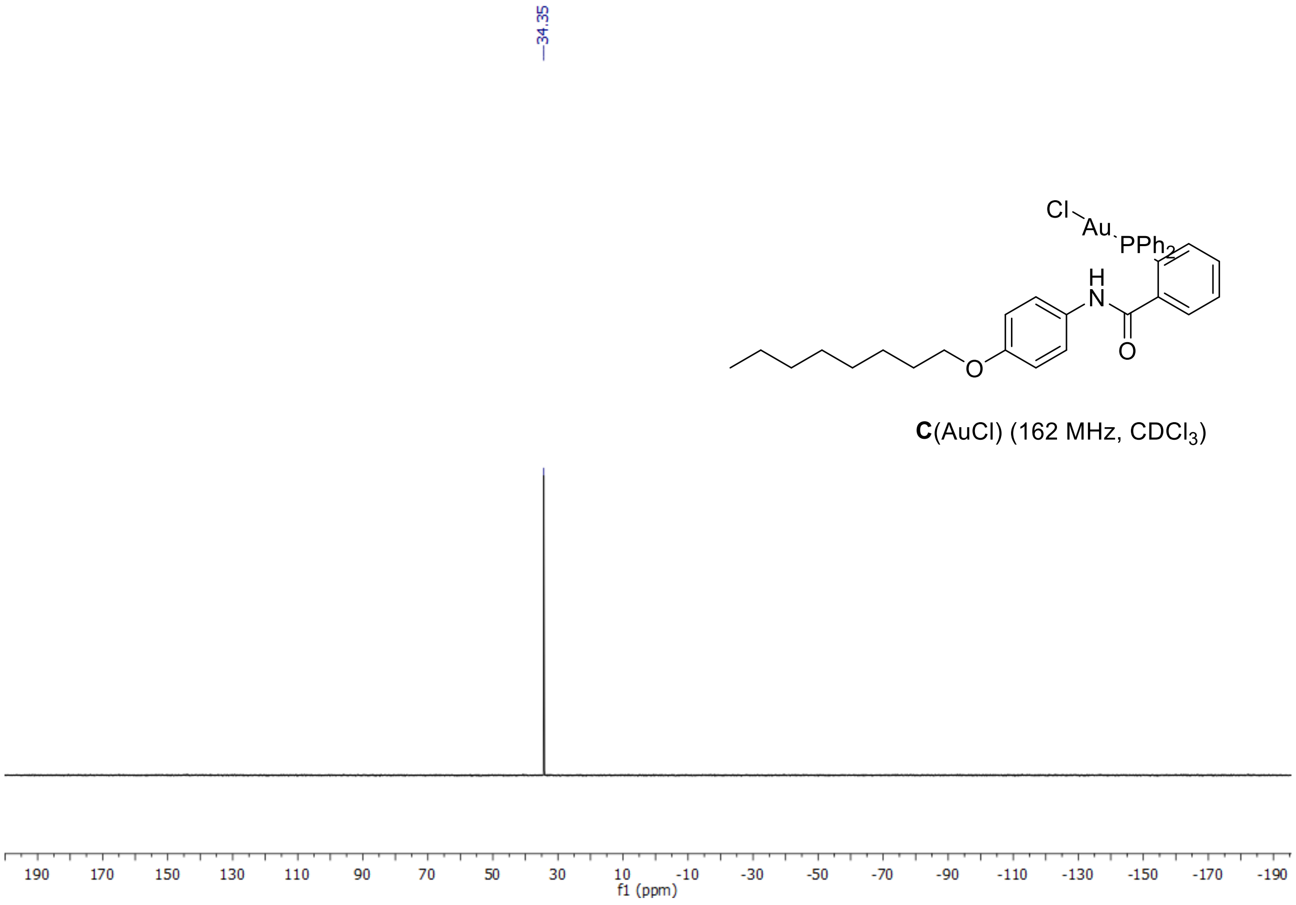
34.35
Cl
Au
PPh2
H
N
O
O
C(AuCl) (162 MHz, CDCl3)
f1 (ppm)

- **References**

[1] M. Bazzoni, V. Zanichelli, L. Casimiro, C. Massera, A. Credi, A. Secchi, S. Silvi, A. Arduini, *Eur. J. Org. Chem.* **2019**, *2019*, 3513-3524.

[2] a) P. Dydio, R. J. Detz, B. de Bruin, J. N. H. Reek, *J. Am. Chem. Soc.* **2014**, *136*, 8418-8429; b) S. Tasan, O. Zava, B. Bertrand, C. Bernhard, C. Goze, M. Picquet, P. Le Gendre, P. Harvey, F. Denat, A. Casini, E. Bodio, *Dalton Trans.* **2013**, *42*, 6102-6109; c) Y. K. Kim, S. J. Lee, K. H. Ahn, *J. Org. Chem.* **2000**, *65*, 7807-7813.

[3] M. E. Krafft, L. V. R. Bonaga, J. A. Wright, C. Hirosawa, *J. Org. Chem.* **2002**, *67*, 1233-1246.
